# An autoinhibitory mechanism controls RNA‐binding activity of the nitrate‐sensing protein NasR

**DOI:** 10.1111/mmi.14517

**Published:** 2020-05-13

**Authors:** Jonathan R. Goodson, Christopher Zhang, Daniel Trettel, Heather E. Ailinger, Priscilla E. Lee, Catherine M. Spirito, Wade C. Winkler

**Affiliations:** ^1^ Department of Cell Biology and Molecular Genetics The University of Maryland College Park MD USA; ^2^ Department of Chemistry and Biochemistry The University of Maryland College Park MD USA; ^3^ FIRE: The First‐Year Innovation & Research Experience Program The University of Maryland College Park MD USA

**Keywords:** antiterminator proteins, bacteria, gene expression, nucleic acid, regulatory sequences, RNA‐binding proteins, transcription attenuation

## Abstract

The ANTAR domain harnesses RNA‐binding activity to promote transcription attenuation. Although several ANTAR proteins have been analyzed by high‐resolution structural analyses, the residues involved in RNA‐recognition and transcription attenuation have not been identified. Nor is it clear how signal‐responsive domains are allosterically coupled with ANTAR domains for control of gene expression. Herein, we examined the sequence conservation of ANTAR domains to find residues that may associate with RNA. We subjected the corresponding positions of *Klebsiella oxytoca* NasR to site‐directed alanine substitutions and measured RNA‐binding activity. This revealed a functionally important patch of residues that forms amino acid pairing interactions with residues from NasR’s nitrate‐sensing NIT domain. We hypothesize these amino acid pairing interactions are part of an autoinhibitory mechanism that holds the structure in an “off” state in the absence of nitrate signal. Indeed, mutational disruption of these interactions resulted in constitutively active proteins, freed from autoinhibition and no longer influenced by nitrate. Moreover, sequence analyses suggested the autoinhibitory mechanism has been evolutionarily maintained by NasR proteins. These data reveal a molecular mechanism for how NasR couples its nitrate signal to RNA‐binding activity, and generally show how signal‐responsive domains of one‐component regulatory proteins have evolved to exert control over RNA‐binding ANTAR domains.

## Graphical abstract

Although ANTAR domains are widespread, it is not known how they recognize RNA or are controlled by signal‐responsive domains. We identify the most conserved ANTAR residues and examine whether they contribute to RNA‐binding activity of *Klebsiella oxytoca* NasR. When NasR is in its “off” state, residues from its signal‐responsive domain sequester key RNA‐binding residues as part of an autoinhibitory mechanism. These data provide mechanistic insight into how signaling domains can communicate with RNA‐binding output domains.

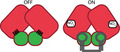

## 1 INTRODUCTION

Transcription attenuation is a process where regulatory factors couple input signals to the control of premature transcription termination (Merino and Yanofsky, 2005). These mechanisms incorporate a cis‐acting regulatory RNA that features both a termination site and a mutually exclusive antiterminator structural element; the decision between formation of the terminator or antiterminator is directed by the presence or absence of the appropriate signal. In some instances, this signal is perceived by an RNA‐binding protein, which acts to either stabilize or destabilize the terminator configuration. Indeed, multiple classes of RNA‐binding proteins control transcription attenuation (Stülke, 2002; Merino and Yanofsky, 2005; Naville and Gautheret, 2010). For example, the *trp* RNA‐binding attenuation protein (TRAP) associates with a tandem series of triplet sequences located within the 5ʹ leader region to block formation of an antiterminator element, thereby allowing an intrinsic terminator structure to form instead (Babitzke and Gollnick, 2001; van Tilbeurgh and Declerck, 2001; Stülke, 2002; McAdams and Gollnick, 2015). For members of the BglG/SacY family of proteins, phosphorylation of their PRD domains (phosphotransferase regulation domain) by the appropriate carbohydrate transport system triggers their RNA‐binding domain to bind to an antiterminator element (Aymerich and Steinmetz, 1992; Raveh *et al.*, 2009; Hübner *et al.*, 2011; Rothe *et al.*, 2012).

Another important family of RNA‐binding proteins contains the “AmiR and NasR Transcriptional Antiterminator Regulator” domain (ANTAR; Pfam: PF03861) (Shu and Zhulin, 2002). The ANTAR domain is a small ~ 12 kilodalton element composed of three helices (O’Hara *et al.*, 1999). ANTAR‐containing proteins typically occur as multi‐domain proteins and fall within several prominent subclasses. One of the most prevalent subclasses consists of ANTAR proteins that participate in two‐component regulatory systems. In these instances, the ANTAR protein is predicted to act as a response‐regulator protein; the *Enterococcus faecalis* EutV response regulator is the best characterized example for this subclass (Del Papa and Perego, 2008). Another prevalent subclass consists of ANTAR proteins that have an N‐terminal pseudo‐receiver domain, suggesting they evolved from two‐component regulatory systems but do not participate in classical sensor kinase‐response regulator interactions. This subclass is best represented by *Pseudomonas aeruginosa* AmiR (O’Hara *et al.*, 1999).

The *E. faecalis* EutV protein increases expression of ethanolamine utilization genes in response to the availability of ethanolamine (Del Papa and Perego, 2008; Fox *et al.*, 2009; Garsin, 2010). Ethanolamine is perceived by the sensor histidine kinase EutW, which then phosphorylates EutV. Phosphoryl transfer causes dimerization of EutV, which improves its overall RNA‐binding affinity. EutV binds at four sites within the *E. faecalis eut* gene cluster, actively preventing formation of intrinsic terminators at each site (Del Papa and Perego, 2008; Fox *et al.*, 2009; Garsin, 2010; Ramesh *et al.*, 2012). A common RNA motif was discovered at these four transcription attenuation sites, which included the full determinants for recognition by the EutV ANTAR domain (Ramesh *et al.*, 2012). This motif consists of a pair of small stem‐loops that display similar terminal loop sequences. In each instance the second hairpin overlaps a mutually exclusive terminator site, suggesting that binding of EutV prevents formation of the intrinsic terminator. After the two‐hairpin motif was discovered in the *eut* locus, it was also identified in the other known ANTAR‐based regulatory systems (e.g., AmiR, NasR and NasT), suggesting that this two‐hairpin motif is likely to constitute a general recognition element of many ANTAR‐based regulatory proteins (Ramesh *et al.*, 2012). However, there is a dearth of information on the determinants that are involved in recognition of RNA ligands by ANTAR proteins. New insight into the molecular basis of the ANTAR‐RNA complex will improve the approaches that are used to predict ANTAR‐based regulons.

A third subclass of ANTAR proteins consists of one‐component regulatory proteins, where ANTAR domains can be found alongside signal‐sensing domains, such as NIT, PAS and GAF domains. Of this large subclass, two representative proteins have been characterized. A light‐responsive regulatory protein from the bacterium *Nakamurella multipartita*, called PAL, contains an RNA‐binding ANTAR domain alongside PAS and LOV domains (Weber *et al.*, 2019). Biochemical analyses showed that RNA‐binding activity was controlled by the light‐sensing LOV domain; however, structural data have only been obtained for the RNA‐free “off” state. The *Klebsiella oxytoca* NasR protein, another ANTAR‐containing, one‐component regulatory protein, has also been investigated by some structural and bacteriological studies. In response to nitrate, NasR activates expression of the *nasFEDCBA* operon, which includes genes involved in nitrate assimilation (Chai and Stewart, 1998; 1999; Shu and Zhulin, 2002). Prior data also showed that the NasR NIT domain responds to binding of either nitrate or nitrite and that the ANTAR domain was required for binding RNA (Chai and Stewart, 1998; 1999; Shu and Zhulin, 2002). A high‐resolution structure of NasR was solved using X‐ray crystallography (Boudes *et al.*, 2012), which revealed a homodimeric complex with a near canonical ANTAR structural configuration (O’Hara *et al.*, 1999; Morth *et al.*, 2004). Given this structural information, we decided to investigate the elements of the NasR ANTAR domain that are generally required for binding RNA. To measure RNA‐binding activity, we developed a sensitive fluorescence anisotropy assay. We then analyzed sequences of ANTAR‐containing proteins to identify universally conserved residues. We targeted those sites for site‐directed alanine substitutions in *K. oxytoca* NasR. This revealed a spatially proximal cluster of residues that are critical for RNA‐binding activity. We propose that these residues are generally important for recognition of RNA by ANTAR proteins. In the structural model of NasR, some of these residues form amino acid pairing interactions with residues from the nitrate‐sensing NIT domain. Utilizing the fluorescence anisotropy assay as well as a transcription antitermination assay we find that the mutational disruptions of these interactions can decouple the NIT domain from the ANTAR domain, leading to nitrate‐independent RNA‐binding activity. From this, we conclude that these amino acid pairing interactions constitute an autoinhibitory mechanism that sequesters RNA‐binding residues in the absence of the nitrate signal. An analysis of conserved residues within NIT domains supports this prediction. Therefore, our data provide a mechanistic model for how one‐component regulatory proteins can couple signal‐responsive domains to ANTAR outputs through an allosteric interaction pathway.

## 2 RESULTS

### 2.1 Identification of NasR‐binding RNA motifs by SELEX

**FIGURE 1:**
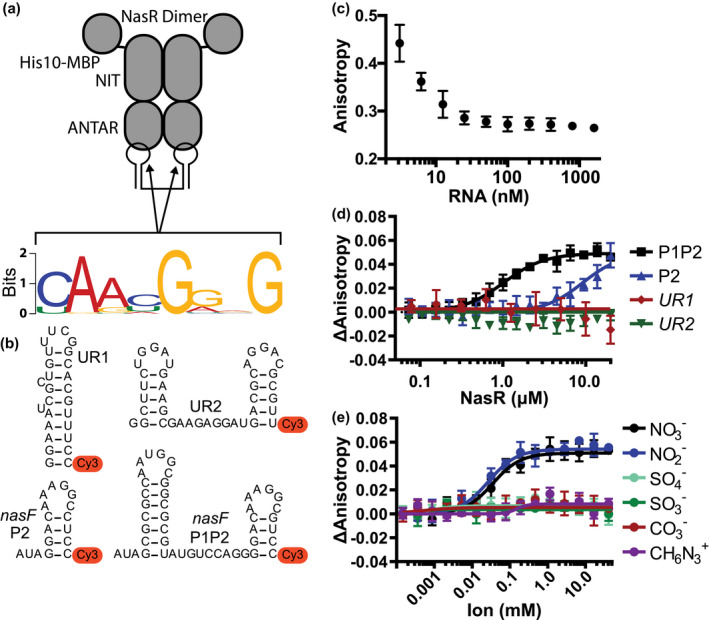
*Klebsiella oxytoca* NasR specifically binds a tandem stem‐loop RNA in the presence of nitrate or nitrite. (a) Purified NasR protein was subjected to systematic evolution of ligands by exponential enrichment (SELEX) and protein‐binding RNA aptamers were analyzed by high‐throughput sequencing. After four rounds of selection, a majority of protein‐associated RNAs included a common consensus motif (shown as a sequence logo) forming the closing base pair and terminal loops of two consecutive hairpins. (b) Cy3‐labeled RNA molecules were synthesized for the two‐hairpin portion of the *Klebsiella oxytoca nasF* leader (“P1P2”), along with two different unrelated RNAs (“UR1” and “UR2”). These RNA molecules were used for fluorescence anisotropy binding assays. (c) Quantification of fluorescence anisotropy of RNA in binding buffer at different concentrations. (d) Equilibrium saturation binding curves of RNA molecules from (b) bound to different concentrations of purified His6‐MBP‐NasR protein in the presence of 1 mM nitrate. (e) Equilibrium binding curves of P1P2 RNA bound to 8 μM His6‐MBP‐NasR protein in the presence of varying concentration of different ionic species. For all panels, error bars represent the standard deviation of the fluorescence anisotropy (b) or change in anisotropy (d‐e) of four replicate wells relative to the no‐protein (d) or no‐ion sample (e). All datasets were fit to a Hill equation model

It was previously shown that NasR can associate in vitro with the 5ʹ leader region of the *nasF* mRNA, when incubated with nitrate (Chai and Stewart, 1999). Yet, these experiments included a large portion of the 5ʹ leader region, more than the two‐hairpin motif that was identified to be the ligand of the *E. faecalis* and *Listeria monocytogenes* EutV proteins (Ramesh *et al.*, 2012; DebRoy *et al.*, 2014; Mellin *et al.*, 2014). To examine its RNA specificity, deca histidine‐tagged, maltose‐binding protein (MBP)‐fused, *K. oxytoca* NasR (His_10_‐MBP‐NasR) was subjected to systematic evolution of ligands by exponential enrichment (SELEX) (Tuerk and Gold, 1990; Bouvet, 2009). Specifically, a large, random library of 30‐nucleotide RNA sequences was incubated with purified protein; the subset of protein‐associated RNAs were retained on an affinity matrix and then subjected to RT‐PCR amplification. A total of four rounds of this selection were completed before subjecting the DNA template pools to high‐throughput sequencing. Analysis of this sequencing data showed a significant increase in abundance in the final round for sequences that agreed remarkably well with the two‐hairpin RNA motif previously shown to bind EutV proteins (Ramesh *et al.*, 2012; Mellin *et al.*, 2014) (Figure 1a). This included a substantial increase in sequences containing two similar stem‐loops in the final round. Specifically, at the top of each stem‐loop is a terminal C:G base pair. Within the terminal hexanucleotide loop there is a clear preference for an A and G at the first and fourth positions respectively. These data demonstrate that the ANTAR domains from EutV and NasR share similar RNA‐recognition properties. Inspection of the *nasF* leader region showed that it contains a two‐hairpin motif that matches the consensus pattern derived by in vitro selection (“P1P2” Figure 1b).

### 2.2 Quantification of RNA‐binding activity by fluorescence anisotropy

To test whether His_10_‐MBP‐NasR associates in vitro with the putative *nasF* two‐hairpin motif (“P1P2”; Figure 1b), we developed a fluorescence based binding assay. The P1P2 RNA was labeled at its 3ʹ terminus with a Cy3 fluorophore. Binding was then tested using fluorescence anisotropy by titrating increasing amounts of the His_10_‐MBP‐NasR dimer to nanomolar quantities of RNA. Although changes in fluorescence anisotropy were detectable with as little as 5 nM RNA, higher concentrations reduced assay noise (Figure 1c). In the presence of 1 mM nitrate, His_10_‐MBP‐NasR bound P1P2 RNA with an apparent equilibrium affinity of approximately 1 μM (Figure 1d). This compares well to a previously reported *K_d_* that was measured by electrophoretic mobility shift assays of NasR against the full *nasF* leader region (Chai and Stewart, 1999). Therefore, the two‐hairpin motif is sufficient for NasR to exhibit full RNA‐binding activity. Additionally, under the same conditions, His_10_‐MBP‐NasR displayed no detectable binding to two unrelated but similarly sized RNA hairpins, both labeled by 3ʹ Cy3 (Figure 1b,d). While both P1 and P2 hairpins are required for full RNA‐binding affinity, NasR binds to single hairpin RNA (Figure 1b, “P2”) with a poorer affinity of approximately 12 μM (Figure 1d). Also, RNA‐binding activity was only observed in conditions containing nitrate or nitrite, with maximum binding occurring above 1 mM KNO_3_ or 500 nM KNO_2_ (Figure 1e), whereas no detectable binding occurred upon addition of sulfate, sulfite, carbonate or guanidinium (Figure 1e).

### 2.3 Identification and site‐directed mutagenesis of conserved ANTAR residues

**FIGURE 2:**
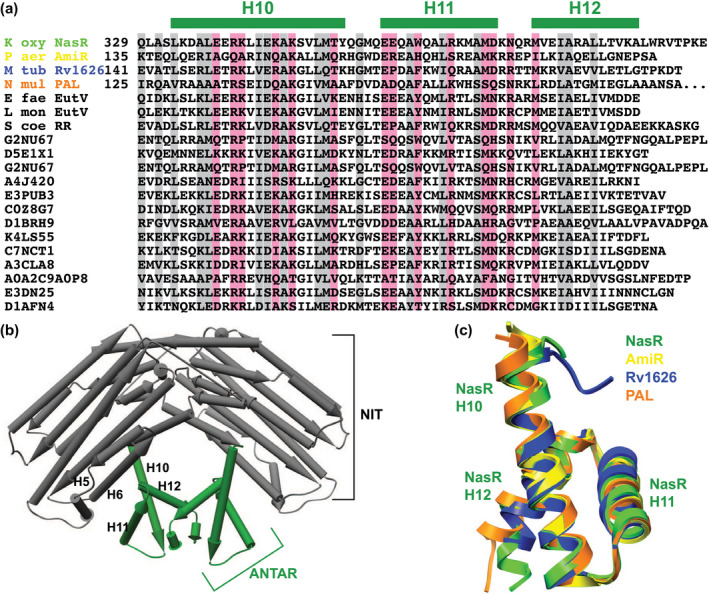
Alignment of representative ANTAR protein sequences. (a) Multiple sequence alignment of nineteen ANTAR domains from ANTAR regulators previously described in the literature (*Klebsiella oxytoca* NasR, *Pseudomonas aeruginosa* AmiR, *Mycobacterium tuberculosis* Rv1626, *Nakamurella multipartita* PAL, *Enterococcus faecalis* EutV, *Listeria monocytogenes* EutV, and a *Streptomyces coelicolor* response regulator protein) in addition to 12 additional representatives of the ANTAR domain selected from Pfam PF03861 listed by UniProt accession ID. Conserved residues selected for mutagenesis are highlighted with magenta. Highlighted in gray are residues that exhibited an increased level of conservation but were not chosen for mutagenesis due to their hydrophobicity and the strong likelihood they participate in interhelical structural interactions. (b) NasR is composed of eight alpha helices in the NIT domain, one connecting helix, and three alpha helices in the ANTAR domain. The location of the ANTAR helices (10‐12) in NasR (PDB: 4AKK) are shown in green. (b) A ribbon depiction of the ANTAR domain region of NasR (PDB: 4AKK) is shown in green, superimposed with structures of the ANTAR domain from *Pseudomonas aeruginosa* AmiR (PDB: 1QO0), *Mycobacterium tuberculosis* Rv1626 (PDB: 1S8N), and *Nakamurella multipartita* PAL (PDB: 6HMJ)

In previous work we demonstrated that the EutV ANTAR domain is sufficient for binding RNA, but inclusion of an adjacent, extended α‐helical region dramatically increased RNA‐binding affinity (Ramesh *et al.*, 2012). Outside of those data, little is known about which residues of ANTAR domains are required for binding RNA ligands and for antitermination activity. To begin investigating these requirements, we aligned sequences of ANTAR domains from proteins predicted to bind the canonical ANTAR RNA motif (AmiR, NasR and EutV) and expanded the alignment to include approximately 8,500 ANTAR domain containing proteins. This comparative sequence analysis revealed 37 positions with amino acids present in at least 40% of sequences, or with a specific chemical class of residues present in the majority of sequences (a representative alignment is shown in Figure 2a and summarized in Figure S1, Table S1 and S2). We located these residues in the high‐resolution structural model of NasR that was previously derived by X‐ray crystallography (Boudes *et al.*, 2012). High‐resolution structures of four ANTAR proteins (NasR, AmiR, Rv1626 and PAL) can be superimposed on their ANTAR portions (Figure 2c), indicating that the overall three‐helical structural architecture is likely to be strictly maintained for the ANTAR domain. Upon inspection of the NasR ANTAR structure, it appeared that 16 of the conserved residues consisted of hydrophobic amino acids that are likely to participate in intramolecular interactions within the protein core, such as the structural interactions formed between alpha helices (Figure 2a). The remaining residues were determined to be candidate sites for protein‐RNA recognition. To investigate their relative importance, we constructed and purified single alanine point mutants of the most conserved residues in the ANTAR domain of His_10_‐MBP‐NasR. Each mutant protein was successfully purified and appeared predominantly as a single consistently sized band when analyzed using SDS‐PAGE (data not shown).

**FIGURE 3:**
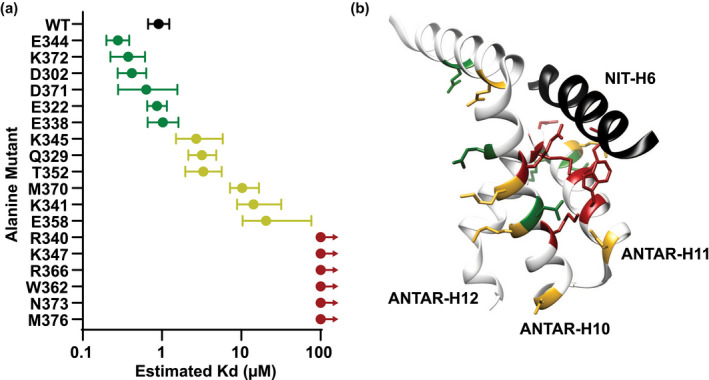
Site‐directed mutagenesis of conserved ANTAR residues affects RNA binding. (a) Equilibrium binding measurements for alanine substitution mutations for His10‐MBP‐NasR in the presence of 1 mM nitrate (full binding curves are shown in Figure S2). Binding measurements were colored according to those exhibiting an affinity better than or comparable to wild‐type NasR (green), those exhibiting greater than 2‐fold reduction in binding affinity (yellow), and those with negligible binding activity (red). (b) Ribbon depictions of the ANTAR domain (PDB: 4AKK) with residues at positions corresponding to the alanine substitutions highlighted in color

We then generated saturation binding curves by fluorescence anisotropy for each of the 18 ANTAR alanine substitutions (Figure S2). The proteins were incubated in the presence of 1 mM nitrate and 50 nM Cy3‐labeled P1P2 RNA. Of the 18 mutants, five bound with comparable or better affinity as compared to wild‐type NasR sequence (Figure 3a). Six mutant proteins exhibited lower affinity than wild‐type sequence, and six other mutant proteins demonstrated a complete loss of RNA‐binding activity (Figure 3a). An analysis of the site‐directed mutations in the context of the NasR three‐dimensional structural model revealed that the most severe mutations clustered at one region of the ANTAR domain (Figure 3b). Manual inspection of the NasR structure revealed two important features of this region: (a) it features several positively charged residues and (b) it is oriented toward a portion of the NIT domain.

### 2.4 Identification of compositional bias for ANTAR and NIT domains

NIT and ANTAR domains are more commonly found in alternative domain compositions. ANTAR domains are more commonly found in combination with response regulator or pseudo‐response regulator domains than with the NIT domain (Finn *et al.*, 2016). Similarly, NIT domains are more often found with histidine kinase domains instead of ANTAR domains (Finn *et al.*, 2016). Yet despite differences in their overall protein architectures, ANTAR domains from NasR, EutV and AmiR all appear to bind to very similar RNA ligands (Chai and Stewart, 1999; Ramesh *et al.*, 2012; Wang *et al.*, 2012). Therefore, we hypothesized that one‐component regulatory proteins like NasR must have evolved a specialized intramolecular signaling path to couple these two regulatory domains and allow for interconversion between “off” and “on” states.

**FIGURE 4:**
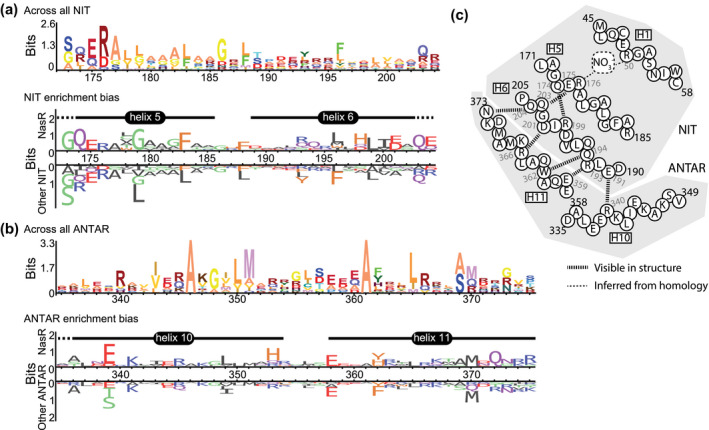
Compositional bias of NIT and ANTAR residues at an interdomain interface. (a) A sequence logo shows conservation of residues across all NIT domain‐containing proteins corresponding to residues in the helix 5‐6 region of *Klebsiella oxytoca* NasR. Letter height represents relative entropy above the background residue distribution frequencies. A modified sequence logo showing the differential information content of NasR proteins and non‐NasR NIT domain proteins identifies residues differentially conserved in the two groups. Letter height represents the relative entropy or KL divergence of the sequence distributions between each group. (b) A sequence logo shows conservation of residues across all ANTAR domain‐containing proteins corresponding to residues in the helix 10‐11 region of *Klebsiella oxytoca* NasR. Letter height represents relative entropy above the background residue distribution frequencies. A modified sequence logo showing the differential information content of NasR proteins and non‐NasR ANTAR domain proteins identifies residues differentially conserved in the two groups. Letter height represents the relative entropy or KL divergence of the sequence distributions between each group. (c) Residues showing conservation bias for NasR‐like proteins cluster at the interdomain interface, as shown in the schematic. Some of these residues appear to participate in amino acid pairing interactions between the NIT and ANTAR domains of NasR as well as part of the hydrogen bonding network connecting this interface with the proposed nitrate binding pocket. Individual residues in helices 1, 5 and 6 (NIT domain) and 10 and 11 (ANTAR domain) are shown in circles. Wide dashed lines represent likely hydrogen bonding interactions between associated residues and narrow dashed lines represent proposed interactions between arginine residues and nitrate

Given the location of the most severe ANTAR mutations (Figure 3), it seemed possible that some of those residues might form meaningful interactions with the NIT domain. We reasoned that if this prediction were true, it might become apparent through comparative sequence analyses. Specifically, we searched for residues that might have been adapted to control ANTAR RNA binding in response to NIT nitrate binding, as compared to other ANTAR architectures (e.g., response regulator proteins, pseudo‐response regulator proteins, PAS/GAF‐ANTAR proteins, etc.). We constructed multiple sequence alignments of approximately 8,500 ANTAR domains and 3,500 NIT domains. We then searched for alignment positions that exhibited unique compositional bias within protein sequences that contained both ANTAR and NIT domains, as compared to the total library of ANTAR and NIT sequences. For each alignment position corresponding to NasR amino acid residues, we calculated a difference between the representation of each amino acid in NIT‐ANTAR proteins (Table S1) and compared it to the representation in the entire alignment (Table S2). This analysis supported our earlier identification of residues that are highly conserved throughout ANTAR domains across all examples. However, a few residues appeared to exhibit measurable biases within NasR homologues (i.e., ANTAR proteins that also contain an NIT domain) (Figures 4a and S1). Only a few of these residues were located in the ANTAR domain, with greater overall compositional bias found within the NIT domain. The relatively small number of biased ANTAR residues may reflect the small size and high structural and functional conservation of the ANTAR domain, features that may restrict the amount of allowable variation. In contrast, the region of the NIT domain comprising helices 5 and 6 of NasR contains numerous residues that show substantially distinct residue compositions in ANTAR‐associated NIT domains (Figure 4a and S1, Tables S1 and S2). Together, the conservation bias of these residues appears to involve two types of contexts: (a) spatially proximal positively charged amino acids and (b) residues that appear to participate in ANTAR‐NIT interdomain interactions.

### 2.5 Site‐directed mutagenesis of positively charged residues

**FIGURE 5:**
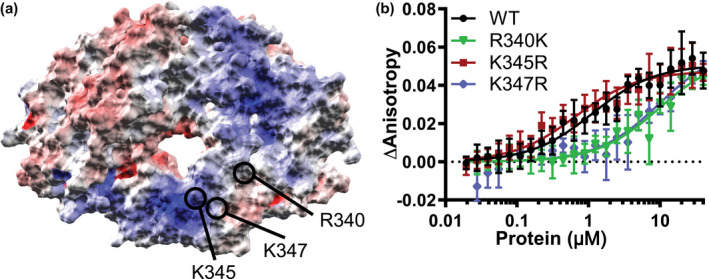
The identity of ANTAR residues in a surface‐associated positive patch can affect RNA binding. (a) A predicted electrostatic surface potential map of the NasR structure (PDB: 4AKK) shows a region of the ANTAR domain dimer with positive surface potential (bottom center). The positions of residues R340, K345 and K347 are demarcated with black circles. K345 is exposed near the center of the positive patch, while R340 and K347 are sequestered by nearby negatively charged residues. (b) Equilibrium saturation binding curves for three charge‐conservative substitution mutant proteins of His10‐MBP‐NasR in the presence of 1 mM nitrate. Error bars represent the standard deviation of the anisotropy change of four replicate wells relative to the no‐ligand sample. The fit lines represent a Hill equation model

It was previously noted that NasR might present a positively charged surface patch (Boudes *et al.*, 2012). The residues involved in that region are strongly conserved across all ANTAR proteins, including the positively charged K341 (Figure S1, Tables S1 and S2). However, some nearby residues are most strongly conserved in NIT‐containing proteins, including enrichment of positions 341 and 345 as lysine or arginine, while others are not strongly conserved in either NasR homologs or ANTAR domains in general, such as positions 372 and 387. To further investigate the importance of this putatively positive‐charged region, we generated three‐dimensional structural and electrostatic models of NasR homologs using the RaptorX and CHARMM‐GUI suites (Figure S3) (Jo *et al.*, 2008a; 2008b; Källberg *et al.*, 2012). These NasR models exhibited significant heterogeneity in locations of positively charged regions across their ANTAR surface, although a small region of positive potential might be consistently detected around the K341/K345 residues (Figure S3). Therefore, while we did not observe a strikingly unique patch of positive residues, as was suggested from the initial analysis of the NasR structure, it is still possible that a small surface‐associated electropositive region might have been enriched across NIT‐ANTAR proteins. In general, positively charged residues of RNA‐binding proteins are oftentimes involved in charge‐charge interactions with the polyanionic RNA backbone. The positive charge of a lysine or arginine side chain might be all that is required for RNA‐binding activity at those sites. To test this possibility for NasR, we mutated three charged residues that may cluster together (R340, K345 and K347), swapping lysine for arginine, or, instead, arginine for lysine (i.e., R340K, K345R and K347R) (Figure 5a,b). Measurements of RNA‐binding activity showed that K345R behaved similar to wild‐type NasR (Figure 5b) and was modestly improved compared to K345A (Figure 3a). However, K347R and R340K each showed a decrease in binding activity, with an apparent affinity around 10‐fold weaker than wild‐type NasR. In the crystallographic structure of NasR, both of these residues are located adjacent to the previously described surface‐accessible electropositive patch (Boudes *et al.*, 2012) but are partially buried by glutamate residues. Therefore, while all three residues appear important for efficient RNA‐binding activity, K347 and R340 residues are required for more than just their local charge contribution and may be directly involved in ribonucleotide interactions.

### 2.6 Identification of interdomain interactions between ANTAR and NIT

In the inactive form of NasR represented by the published structural model, the most substantial interaction between the ANTAR and NIT domains is a hydrophilic interface between parallel helices 6 and 11 in the NIT and ANTAR domains respectively. It is in this interface where the most detrimental alanine substitutions are located (Figure 3b). It is also in this vicinity where several residues exhibit compositional bias within NIT‐ANTAR proteins (Figures 4a and S1, Tables S1 and S2). For example, in NIT‐ANTAR proteins, the residue corresponding to W362 is never represented by a phenylalanine, which is commonly observed in other ANTAR proteins, but is instead represented by the other aromatic amino acid residues (Figures 4b and S1, Tables S1 and S2). In NasR, this residue appears to form a pairing interaction with an NIT domain residue, Q194 (Figure 4c). Mirroring W362, Q194 also exhibits compositional bias; this position is conserved most often as an arginine across all NIT‐containing proteins, yet, in ANTAR‐associated NIT proteins, it is modestly enriched as a glutamine. This paired bias suggests that a W362‐Q194 pairing interaction has been specifically selected by ANTAR‐NIT proteins. Importantly, W362A is one of the mutations that severely reduced RNA‐binding activity (Figure 3a). From these data, we speculate that W362 is likely to be involved in RNA recognition, perhaps through nucleobase stacking interactions, but can be sequestered into a pairing interaction with Q194 when the protein is held in the “off” state.

Similarly, ANTAR residues E359, R366 and N373 showed moderate enrichment in NIT‐associated ANTAR domains (Figures 4b and S1, Tables S1 and S2). All three of these residues also appear to participate in amino acid pairing residues with partner residues from the NIT domain (Figure 4c). Strikingly, their respective NIT partner residues R193, D201 and Q204 show greater evidence of enrichment in ANTAR‐containing proteins (Figures 4a and S1). Therefore, these four residue pairs (R193‐E359, Q194‐W362 D201‐R366 and Q204‐N373) appear to have been modestly evolutionarily enriched for interdomain interactions in NasR‐like proteins. Nearby, yet another amino acid pair is formed between E191 and helix 10 R340. R340 is near‐universally conserved in both NIT‐ and non‐NIT‐associated ANTAR domains (Figures 4b and S1) and is essential for RNA‐binding activity (Figure 3a). Given the importance of R340, we propose that the “sequestration” of R340 by E191 might also play a key role in regulating ANTAR RNA‐binding activity. Other residues also exhibit enrichment for NIT‐ANTAR proteins, perhaps acting as participants in the allosteric coupling of the nitrate sensing NIT domain with ANTAR; further functional analyses are required to elucidate the roles of these residues.

### 2.7 Mutagenesis of interdomain interactions between ANTAR and NIT support an autoinhibitory mechanism

**FIGURE 6:**
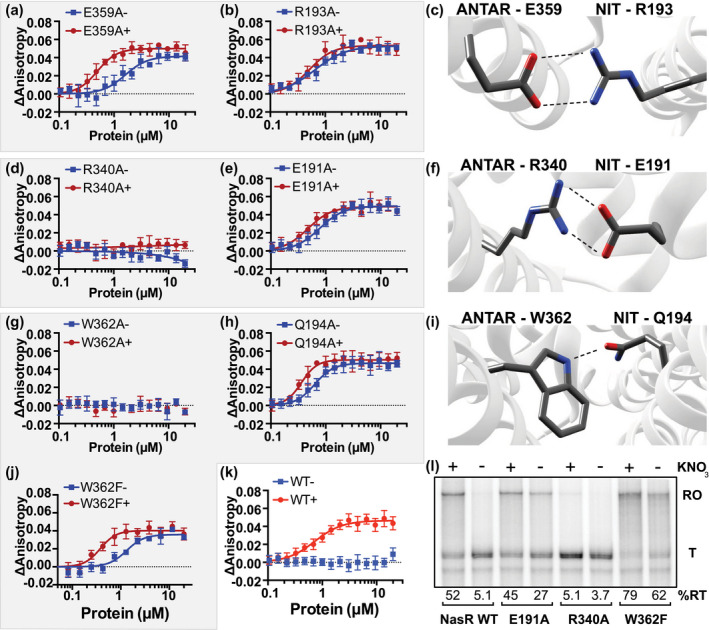
Amino acid interactions at the NIT‐ANTAR interface are required for control of RNA‐binding activity. (a‐k) Equilibrium saturation binding curves for alanine substitution mutants of residues near the NIT‐ANTAR interdomain interface. Blue data points represent binding curves performed in the absence of nitrate, while red data points represent binding curves with 1 mM nitrate. Error bars represent the standard deviation of the anisotropy change of four experiments relative to the no‐ligand sample. The fit lines represent a Hill equation model. (l) Site‐directed mutations that perturb interdomain interactions affect transcription attenuation by NasR. DNA templates encompassing the *K. oxytoca nasF* leader region were amplified by PCR and incubated with *E. coli* RNA polymerase (New England Biolabs) and P^32^‐radiolabeled UTP. These reactions also contained 8 μM purified His10‐MBP‐NasR proteins and were incubated in the presence or absence of 1 mM nitrate. The products of these transcription reactions were resolved by 8% urea‐denaturing polyacrylamide gel electrophoresis (PAGE). This resulted in two primary transcription products, one that corresponds to premature transcription termination (“T”) and a longer transcript corresponding to run‐off transcription (“RO”). The fraction of RO was determined for wild‐type protein and each of the mutant proteins

Our bioinformatic analysis suggested a modest evolutionary enrichment for residues participating in five interdomain pairing interactions between ANTAR helices 10‐11 and NIT helix 6 (Q204‐N373, D201‐R366, R193‐E359, E191‐R340 and Q194‐W362). Correspondingly, we introduced site‐directed alanine substitutions to test the significance of these interactions on RNA‐binding activity (Figure 6). Both ANTAR N373A and R366A mutant proteins lacked RNA‐binding activity (Figure 3a); however, mutagenesis of their NIT pairing partners (Q204 and D201, respectively) had little effect (Figure S3).

Alanine substitution at ANTAR E359 showed near wild‐type binding with nitrate and moderately reduced binding in the absence of nitrate (Figure 6a); however, alanine substitution of its pairing partner R193 (Figure 6b) resulted in a protein that lacked nitrate‐responsiveness (i.e., exhibited constitutive RNA‐binding activity). Similarly, alteration of R340 eliminated RNA‐binding activity, yet mutagenesis of its pairing partner E191 resulted in constitutive RNA‐binding activity (Figure 6d,e). The W362A mutation also resulted in loss of RNA‐binding activity (Figure 6g), and mutagenesis of its partner Q914 once again resulted in constitutive RNA‐binding activity (Figure 6h). In non‐NIT proteins, position 362 is usually represented by a phenylalanine, whereas, in NIT proteins, it is usually represented by one of the other aromatic residues (e.g., tyrosine; Figure 4a). From this observation, we hypothesize that RNA‐binding activity requires an aromatic residue at this position but that NasR proteins specifically utilize a functionally important amino acid pairing interaction between the NIT and ANTAR domains. To test this hypothesis we altered 362 from tryptophan to a phenylalanine and measured RNA‐binding activity. The phenylalanine substitution preserves or even increases RNA‐binding activity in the presence of nitrate (Figure 6j). And, indeed, although RNA‐binding activity is modestly reduced, W362F retains substantial RNA‐binding activity in the absence of nitrate, suggesting it has been partially decoupled from the NIT domain, while retaining RNA‐binding activity. Together, these observations support our hypothesis that domain–domain interactions (Figure 6c,f,i) have been evolutionarily programmed to restrict function of key RNA‐binding ANTAR residues. More specifically, our data suggest that E191‐R340, R193‐E359 and Q194‐W362 help create an autoinhibited off‐state in the absence of nitrate, which is likely to be released upon binding of nitrate by the NIT domain. We propose that the release of residues R340 and W362 are particularly essential for ANTAR‐RNA interactions.

### 2.8 Mutagenesis of interdomain interactions affects NasR regulation

To test whether our site‐directed mutagenesis data are functionally relevant for NasR regulation, we tested some of the mutant proteins for transcription attenuation activity. Specifically, bacterial RNA polymerase was incubated with a DNA template that encompasses the *K. oxytoca nasF* leader region, both in the presence and absence of nitrate. This resulted in two transcription products, a shorter transcript for premature transcription termination and a longer transcript from readthrough of the intrinsic terminator. In the presence of nitrate, 52% of the transcripts bypassed the terminator site, in contrast to only 5% in the absence of nitrate. Mutation of R340A resulted in a loss of antitermination activity. In contrast, mutation of E191, which is likely to form a pairing interaction with R340, resulted in an increase in readthrough activity irrespective of nitrate. Similarly, the W362F mutation resulted in elevated antitermination activity both in the presence and absence of nitrate. These data agree remarkably well with measurements of NasR’s RNA‐binding activity and indicate that the autoinhibitory amino acid pairing interactions identified in this study are functionally relevant for genetic regulation by NasR.

## 3 DISCUSSION

The ANTAR domain is widespread and can be found in diverse protein architectures (Shu and Zhulin, 2002; Ramesh *et al.*, 2012). The ANTAR proteins that have been investigated have been shown to couple cellular signals to the regulation of transcription attenuation. Although the activity and biological roles of the ANTAR domain have been studied, its mechanism of RNA recognition remains relatively unexplored. The three most common architectures of ANTAR‐containing proteins are those that: (a) contain response regulator receiver domains and are presumably paired with a cognate sensor histidine kinase, (b) contain a pseudo‐receiver domain that is regulated through protein‐protein interactions by a signal‐responsive protein or (c) are one‐component regulatory proteins that include signal‐responsive domains, such as NIT, GAF and PAS domains (Shu and Zhulin, 2002; Finn *et al.*, 2016). The ANTAR domains are conserved between these various proteins; however, it has been unclear whether the ANTAR domains have become specialized within these different signaling contexts. From our study herein, we propose that ANTAR domains are not specialized but rather their intrinsic RNA‐binding activity is simply employed in different ways, depending on the overall protein architecture.

ANTAR domains are small, at only around 12 kilodaltons in size (Shu and Zhulin, 2002). In this study, we identified the amino acid residues that exhibited the highest degree of conservation across all ANTAR proteins. The relative importance of these positions was probed via RNA‐binding assays using proteins that contained site‐directed alanine substitutions. This revealed that the residues most important for binding RNA are localized to one region of the ANTAR domain. Given the conservation of these residues, we speculate they are broadly important for RNA recognition by ANTAR proteins, not just for NasR. However, in the published structural model of *K. oxytoca* NasR this portion of ANTAR happens to face inward toward the nitrate‐sensing NIT domain. This would suggest these residues associate with the NIT domain and would, therefore, be unavailable for binding RNA. However, all structural information on ANTAR proteins has been acquired only for unbound proteins. Therefore, we reasoned that the “off” state of NasR comprises an autoinhibited conformation that may require significant changes involving these portions of the molecule in order to shift to an “on” state. To provide the experimental support for this hypothesis we aligned and analyzed sequences of many ANTAR containing proteins, as well as many NIT‐containing proteins, and searched for residues that were specifically enriched in NIT‐ANTAR protein architectures, as compared to the other protein domain arrangements. This uncovered compositional bias for residues at the NIT‐ANTAR interface. Furthermore, some of these residues appear to be involved in amino acid pairing interactions between the NIT and ANTAR domains and involve residues essential for RNA binding. Mutational disruption of these amino acid pairs resulted in proteins that were either inactive for binding RNA, presumably because the mutated ANTAR residue was required for direct RNA interactions, or proteins that were constitutively active, presumably because the mutated NIT residue had “broken” the autoinhibitory mechanism, thus freeing the ANTAR domain from its interdomain association with NIT. However, it is also worth noting that, while our data have demonstrated the presence of key autoinhibitory NIT‐ANTAR interactions, they have not revealed how these amino acid pairing interactions are specifically coupled with the nitrate binding pocket. Helix 5 contains residues (E175 and R176) that were previously predicted to participate in the nitrate binding pocket (Boudes *et al.*, 2012). Our data suggest that there are multiple other residues along helix 5 that are more prevalent in NIT‐ANTAR proteins than in other classes of NIT proteins (Figure 4a, Tables S1, and S2). This includes but is not limited to amino acid pairing interactions (e.g., E175‐Q203 and Q174‐R199) that appear to bridge helices 5 and 6 (Figure 4a,c). While a Q203A mutant showed no loss of activity, a E175A mutant was no longer capable of binding RNA, suggesting that residues in this vicinity are, indeed, involved in the regulatory pathway (Figure S2). Given that an assortment of helix 5 residues are enriched in NasR proteins, it is likely that the interactions between helices 5 and 6 are key to the allosteric pathway between the nitrate pocket and the autoinhibitory NIT‐ANTAR interactions. Further structural analyses, perhaps with some of the mutant proteins characterized herein, will be required to test this hypothesis.

If our model for autoinhibition of NasR is correct, it would suggest that the NasR ANTAR domain is constitutively active, and that it does not require global structural changes to become active in response to the nitrate signal. Instead, residues that are key to RNA recognition (across all ANTAR proteins) are simply released from inhibitory pairing interactions in a signal‐dependent manner. In support of this hypothesis, a circular dichroism analysis of the NasR protein in the presence and absence of nitrate revealed no fundamental differences in global secondary structure (Figure S5). By extension, this would suggest that the NIT domains of NasR homologues were evolutionarily adapted to exert regulatory control over ANTAR domains. This is modestly supported by the patterns of conservation biases; more residues were strongly enriched in NIT domains of NIT‐ANTAR proteins than in the ANTAR domains of the same protein sequences.

**FIGURE 7:**
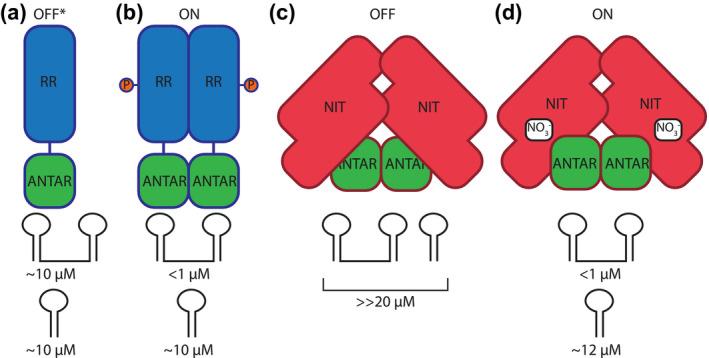
Summary model for one‐ and two‐component ANTAR regulatory proteins. (a) The default state of response regulator ANTAR proteins is presumed to be that of a monomer. These monomers bind either one‐ or two‐hairpin RNA with a similar low affinity. (b) Upon phosphorylation by the cognate histidine kinase, the protein dimerizes—binding both hairpins of a two‐hairpin RNA with an increased apparent affinity—but retains low‐affinity for single‐hairpin RNA. (c) NasR‐like proteins (containing NIT and ANTAR domains) form a constitutive dimer incapable of binding RNA. (d) Upon ligand binding, these one‐component regulators adopt an alternate configuration. Autoinhibitory interactions are disrupted, thereby releasing the RNA‐binding face of the ANTAR domain to bind RNA with apparent affinity comparable to dimerized response regulator ANTAR proteins

In contrast to NasR, previous data showed that RNA‐binding activity of the response regulator EutV protein was improved by EutV dimerization and not specifically through structural interference of ANTAR residues (Figure 7). Essentially, the response regulator ANTAR protein is always active for binding RNA, but the equilibrium affinity for binding the two‐hairpin RNA motif is simply lower with a protein monomer (~10 μM) than it is for a dimer (~1 μM). Yet, for one‐component regulatory proteins like NasR, which are always dimeric proteins, a similar outcome can be accomplished via an autoinhibitory mechanism outlined herein, despite using essentially the same ANTAR domain sequences. Together, these data provide a simple molecular mechanism to explain how the same basic ANTAR sequences can be used in different signaling contexts. We anticipate a majority of other ANTAR proteins to fall within these two regulatory archetypes—signal‐induced dimerization of constitutively active ANTAR domains and signal‐induced release of autoinhibitory interactions for constitutively dimeric ANTAR proteins. Therefore, investigations on NasR are likely to provide a preview of the autoinhibitory mechanisms used by other one‐component ANTAR proteins. In general, elucidating how ANTAR domains recognize RNA substrate(s) will provide key information that can be used to predict ANTAR‐based regulatory networks in diverse organisms.

## 4 MATERIALS AND METHODS

### 4.1 Chemicals and oligonucleotides

All chemicals and enzymes, unless otherwise noted, were purchased from Sigma‐Aldrich and New England Biolabs respectively. DNA oligonucleotides were purchased either from Integrated DNA Technologies, Inc. or from Sigma‐Aldrich. Exact nucleotide sequences and brief description of DNA oligonucleotides used in these studies can be found in Table S3.

### 4.2 Growth conditions

All *E. coli* strains were cultured at 37°C in Luria Bertani (LB, Difco) broth or agar (10 g tryptone, 5 g yeast extract, 5 g NaCl per liter). When appropriate, antibiotics were used at the following concentrations for *E. coli*: ampicillin, 50 μg/ml; carbenicillin, 100 μg/ml and gentamicin, 15 μg/ml.

### 4.3 Strain construction

Codon optimized NasR (Integrated DNA Technologies) was amplified and Gibson assembled (Gibson *et al.*, 2009) in‐frame with a His_10_‐MBP tag using NdeI‐ and BamHI‐digested pVL847 (Lee *et al.*, 2007). Point mutations of NasR coding sequence were constructed using the Q5 Site‐Directed Mutagenesis system (NEB) and transformed into *E. coli* as described below.

### 4.4 Genetic transformation of *E. coli*

All Gibson assembly, Q5 site‐directed mutagenesis and subcloning reactions were transformed as follows: 5 μl of the cloning reaction was transformed into 50 μl of chemically competent XL10‐Gold *E. coli*. The DNA was incubated with the cells for 30 min on ice. The mixture was heat‐treated at 42°C for 30 s followed by recovery on ice for 5 min. 250 μl of rich media was added to the cells for an hour outgrowth at 37°C. 100 μl of the reaction was plated on LB agar with the appropriate antibiotic. Plasmids for the expression of proteins were additionally transformed into T7 Express LysY/I_q_ *E. coli* (NEB) following the same method.

### 4.5 In vitro selection for NasR‐binding RNAs

#### 4.5.1 Methods for SELEX, sequencing and data analysis are found in methods supplement

##### Protein expression and purification

Deca‐histidine‐tagged NasR was cultured in 2xYT and expression induced in cells at A_260_ = 0.5 with 1 mM IPTG at room temperature for 18 hr. The cell pellet was resuspended in resuspension buffer (50 mM HEPES pH 8.0, 150 mM NaCl, 1 mM MgCl_2_, 2 mM β‐ME, 1 mM PMSF, 2 U DNase and 0.5 mg/ml lysozyme). The cell suspension was then disrupted bead‐beating or sonication for intervals of 1 min on, 1 min off, on ice, for 5 total minutes of disruption. After cell disruption cell lysates were clarified by centrifugation at 12,000*g* for 30 min. The clarified supernatant was passed over Ni‐NTA resin columns (Thermo Scientific, GE Healthcare), followed by six column‐volumes (CV) wash buffer (50 mM HEPES pH 8.0, 150 mM NaCl, 1 mM MgCl_2_, 35 mM Imidazole). The protein was eluted in three fractions of one CV elution buffer (50 mM HEPES pH 8.0, 150 mM NaCl, 1 mM MgCl_2_ and 250 mM Imidazole). Eluted protein was dialyzed against dialysis buffer (50 mM HEPES pH 8.0, 150 mM NaCl and 1 mM MgCl_2_) for three sequential 2‐hour buffer exchanges, or alternately buffer exchanged using HiPrep Sephadex G‐25 Desalting columns (GE Healthcare). All steps were performed either on ice or at 4°C. The purity of NasR was judged by 4%‐20% SDS/PAGE followed by Coomassie‐staining.

##### RNA‐binding assays using fluorescence anisotropy

Purified protein was diluted to the initial maximum concentration with Binding Buffer (50 mM HEPES pH 7.5, 100 mM KCl, 1 mM MgCl_2_ and with or without 1 mM KNO_3_). Serial dilutions were performed on the purified protein in binding buffer. Each of the diluted protein samples were added to 100 nM Cy3‐labeled RNA in a 1:1 (v:v) ratio and mixed. Each mixture aliquoted into four wells of a 384‐well black‐bottomed plate and spun at 2,000 rpm for 1 min. The plates were then incubated in the dark for 30 min to reach equilibrium. The fluorescence polarization was then read in a Molecular Devices Spectramax M5 plate reader at 535 nM excitation and 580 nM emission. Data were fit using GraphPad Prism 6 using the single‐site specific binding Hill equation model.

##### RNA transcription antitermination assay

Each 10 μl of reaction mixture included 1× *E. coli* RNA Polymerase Reaction Buffer (New England Biolabs), 500 μM ATP, 500 μM CTP, 500 μM GTP, 500 μM UTP, 250 μM DNA template (JRG105/MG327 PCR product from pMG1127), 8 μM purified NasR, 0.5 units of *E. coli* RNAP, ~3 pmol of α‐P^32^‐UTP (Perkin Elmer). The 10 μl of reaction mixture was then incubated at 37°C for 1 hr and then one volume of 2× denaturing gel loading buffer (0.09 M tris, 0.09 M borate, 10 mM EDTA (pH 8.0), 18 M urea, 20% sucrose, 0.1% SDS, 0.05% bromophenol blue, 0.05% xylene cyanol) was added. The resulting mixture was resolved by urea‐denaturing PAGE on a 8% gel. The resulting gel was then dried and exposed to a phosphor screen for analysis on a phosphor imager. Band intensities were quantified using the FIJI/ImageJ gel quantification plugin (Schindelin *et al.*, 2012; Schneider *et al.*, 2012).

## Supporting information

Supplementary Material
