# Supplementary material for: An autoinhibitory mechanism controls RNA‐binding activity of the nitrate‐sensing protein NasR
Source: Mol Microbiol. 2020 May 13;114(2):348–60. doi: 10.1111/mmi.14517 (PMC7496416; doi:10.1111/mmi.14517)
Supplement: Supplementary file 1 — Supplementary Material [file MMI-114-348-s001.pdf]

An Autoinhibitory Mechanism Controls RNA Binding Activity of the Nitrate-Sensing Protein NasR

Running Title: Analysis of RNA-binding by the NasR ANTAR domain

Jonathan R. Goodson[1,2], Christopher Zhang[1,2], Daniel Trettel[3], Heather E. Ailinger[4], Priscilla E. Lee[4], Catherine M. Spirito[4], Wade C. Winkler[1,3,4,5]

[1]The University of Maryland, Department of Cell Biology and Molecular Genetics, College Park, MD 20742

[2]Equal contribution

[3]The University of Maryland, Department of Chemistry and Biochemistry, College Park, MD 20742

[4]The University of Maryland, FIRE: The First-Year Innovation & Research Experience Program, College Park, MD 20742

[5]Corresponding author

Contact information:
Wade C. Winkler
The University of Maryland
Department of Cell Biology and Molecular Genetics
3112 Biosciences Research Building
College Park, MD 20742
Tel. 301-405-7780
e-mail: wwinkler@umd.edu

SUPPLEMENTARY INFORMATION:

Supplementary Methods. Methods descriptions for the steps involved in *in vitro* selection and sequences of RNA molecules binding to *K. oxytoca* NasR.

Figure S1: Compositional bias of NIT and ANTAR residues at an interdomain interface. (A) Representations of overall residue conservation and differential conservation between NasR homologs (~230 sequences) and other ANTAR domain-containing proteins (~8200 sequences) or other NIT domain-containing proteins (~3200 sequences). Each circle represents the presence of a particular amino acid at the alignment position represented by the NasR residue at each position along the left. The area of each circle is directly proportional to the fraction of all sequences at that position containing each amino acid, up to maximum size at 50% conservation among all sequences. The color of each circle represents the difference in proportion between NIT-ANTAR pair proteins (NasR homologs) against the overall alignment. Red represents amino acids that are less prevalent in NasR homologs compared to the overall alignment; blue represents those amino acids more prevalent in NasR homologs. Grey circles represent amino acids that are conserved at similar frequencies for NasR-like proteins and the overall alignment.

Figure S2: Site-directed mutagenesis of conserved ANTAR residues affects RNA binding. (A-C) Equilibrium saturation binding curves for nineteen alanine substitutions mutations for His10-MBP-NasR in the presence of 1 mM nitrate. Residues were grouped into (A) those with affinity better than or comparable to wild-type NasR, (B) those exhibiting greater than 2-fold reduction in binding affinity, and (C) those with negligible binding activity. For all panels, error bars represent the standard deviation of the anisotropy change of four replicate samples relative to a no-ligand sample. All datasets were fit to a Hill equation model. (D) Ribbon depictions of the ANTAR domain (PDB: 4AKK) with residues at positions corresponding to the alanine substitutions highlighted in color. Green residues correspond to mutations described in (A). Yellow residues correspond to mutations described in (B). Red residues correspond to mutations described in (C).

Figure S3: Electrostatic surface potential models of computationally predicted NasR homolog structures. Electrostatic surface potential for NasR (PDB: 4AKK) (A) or NasR homolog sequences (B-I) was predicted for structure models predicted by RaptorX using the CHARMM-GUI suite. The region corresponding to the positive patch of the ANTAR domain in *K. oxytoca* NasR is enclosed in a square for each structure. UniProt accession numbers for sequences used for structure prediction from each genome: (B) *Nitrincola nitratireducens* (W9V209) (C) *Acidovorax temperans* (A0A0D7KAF4) (D) *Halomonas sp. JB380* (A0A1R4HZH8) (E) *Erwinia tasmaniensis* (B2VKH7) (F) *Burkholderia cenocepacia* (B4E5J8) (G) *Pseudomonas stutzeri* (A4VRW3) (H) *Serratia ficaria* (A0A240C4M6) (I) *Klebsiella pneumoniae* (A6TAM9).

Figure S4: Assessment of helix 5 and helix residues for control of RNA binding activity. Equilibrium saturation binding curves for alanine substitution mutants of residues

near the NIT-ANTAR interdomain interface or within helix 5, as described in the text. Blue data points represent binding curves performed in the absence of nitrate, while red data points represent binding curves with 1 mM nitrate. Error bars represent the standard deviation of the anisotropy change of four experiments relative to the no-ligand sample. The fit lines represent a Hill equation model.

Figure S5: Circular dichroism spectroscopy of NasR proteins. NasR was purified as described and dialyzed into 10 mM sodium phosphate buffer (pH 7.5). Samples were then supplemented with either 100 µM KNO_3 or Na_2SO_3 at a final concentration of 10 µM and verified spectrophotometrically using extinction coefficients calculated in ProtParam. CD spectra were obtained on a Jasco J-1500 spectropolarimeter at 22˚C in a 1 mm quartz cuvette. Each spectra is an average of two scans taken at 50 nm/min from 260 to 190 nm in intervals of 1 nm. HT values never exceeded 600 V. Scans of samples containing NasR were buffer subtracted and converted to molar ellipticity before having their secondary structure elements analyzed in Spectra Manager's Multivariate SSE program.

Table S1: Relative representation of amino acids in alignments of NasR homologs. For each position corresponding to a residue in *K. oxytoca* NasR in the multiple sequence alignment of NIT domains (positions 41-278) or ANTAR domains (positions 331-384) for proteins containing both NIT and ANTAR domains, the percentage of sequences with each amino acid at that position is shown. These data were compared with that in Table S1 to generate the data for Figure 4. (Found in separate Excel spreadsheet)

Table S2: Relative representation of amino acids in alignments of NIT and ANTAR domains. For each position corresponding to a residue in *K. oxytoca* NasR in the multiple sequence alignment of NIT domains (positions 41-278) or ANTAR domains (positions 331-384), the percentage of sequences with each amino acid at that position is shown. These data were compared with that in Table S2 to generate the data for Figure 4. (Found in separate Excel spreadsheet)

Table S3: List of strains used. Each of the strains used in the paper is represented in table S3. From left to right the columns represent the name of the completed strain, the base strain used to create the strain, the plasmids in the strain, and what each plasmid in the strain is composed of.

Table S4: List of oligonucleotides used. Each of the oligonucleotides used in the paper is represented below. From left to right the columns represent; what mutation the oligonucleotides was used for, what the oligonucleotide was used for, the name of the oligonucleotide, and the sequence of the oligonucleotide.

Supplementary Methods. Methods descriptions for the steps involved in *in vitro* selection and sequences of RNA molecules binding to *K. oxytoca* NasR.

In vitro selection for NasR-Binding RNAs

Two separate in vitro selections for RNA aptamers to NasR were performed. One selection utilized filter-binding of the His-MBP-NasR protein to a nitrocellulose filter for separation [1], while the other used binding of the His-tagged protein to HisPur Ni-NTA magnetic beads [2]. Both selections began from the TriLink N30 RNA library for in vitro selection.

For filter-binding, we performed an initial negative selection by passaging approximately 500 µL of 800 nM RNA library in binding buffer (50 mM HEPES, 100 mM KCl, 1mM $MgCl_2$) over a nitrocellulose filter. For positive selection, we then mixed this RNA with 500 µL of 600 nM His-MBP-NasR protein and incubated for 30 minutes. We conducted selection by passing this solution over a 25 mm nitrocellulose filter at approximately 1 drop/second. We then washed the filter with 3 mL of binding buffer in the same fashion. We eluted our RNA and protein by placing the filter in a microcentrifuge tube with 100 µL binding and incubating at 95C for 5 minutes, repeating this step once.

For Ni-NTA selections, we performed an initial negative selection by incubating 100 µL of 5 µM RNA library (500 pmol) and 5 µM His-MBP with 30 µL of HisPur Ni-NTA beads in binding buffer (50 mM HEPES, 100 mM KCl, 1mM $MgCl_2$) for 5 minutes at room temperature, then placing on a magnetic stand to separate the beads and retaining the supernatant. For positive selection, we then mixed the RNA with 10 µL of 50 µM His-MBP-NasR and 30 µL of Ni-NTA magnetic beads and incubated for 10 minutes at room temperature. We then placed the tube on a magnetic stand to separate the beads and remove the supernatant. We then resuspended the beads in 500 µL of binding buffer, and repeated the washing three times. Finally, we transferred the beads to a new tube and placed on a magnetic stand, removing the supernatant. We eluted the bound protein and RNA by incubating the beads with Zymo RNA Clean & Concentrator Binding Buffer with 250 mM imidazole for five minutes, placing the beads on the magnetic stand, and retaining the supernatant.

For each cycle, from this step onward both selections used a similar protocol. We cleaned the eluted RNA using the Zymo RNA Clean & Concentrator kit, eluting in 25 µL water. We then reverse-transcribed this RNA to make cDNA using the standard SuperScript IV (filter-binding) or ProtoScript II (Ni-NTA) protocols with 25 µL reactions and 5 µL of input RNA and the selection reverse primer (TriLink). We then performed cycle-course PCR using 2 µL of this cDNA in 40 µL reactions using Taq DNA polymerase and the selection forward and reverse primers (TriLink), removing 2 µL of PCR reaction every two cycles from rounds 10 to 30. We ran these aliquots on a 2% TAE agarose gel to determine the cycle at which DNA was visibly amplified and the number of cycles required for PCR amplification. We then performed a 400 µL Taq PCR reaction using the number of cycles determined by the cycle-course experiment. We cleaned up these reactions using the Zymo DNA Clean and Concentrator columns. Finally, we transcribed 450 ng of this PCR-amplified template DNA using T7 RNAP in a 25 µL reaction, and again cleaned up this

RNA using the Zymo RNA cleanup kit. This RNA was then used to repeat the next round of selection. Subsequent rounds did not include a negative selection step, and reduced the protein-to-RNA ratio. In total, four selection rounds were performed.

High-throughput sequencing of SELEX RNA pools

For each round of selection, we began from the cDNA prepared during the selection process from each round. We amplified 5 ng of cDNA from each rouch of each selection in standard 50 mL reactions using Q5 DNA Polymerase (Initial denaturation: 98 ºC for 30s, Cycle, 15 rounds: 98 ºC for 10 seconds, 60 ºC for 15 seconds, 72 ºC for 20 seconds, Final extension: 72 ºC for 2 minutes). These PCR products were cleaned using DNA Clean and Concentrator-5 columns (Zymo Research). These reactions were quantified by UV absorbance and normalized. We performed a second PCR reaction with 100 ng total template DNA for each reaction and unique primer pairs from the NEBNext Dual-Index Barcode Set 1 (Initial denaturation: 98 ºC for 30s, Cycle, 4 rounds: 98 ºC for 10 seconds, 65 ºC for 75 seconds, Final extension: 65 ºC for 5 minutes). We again purified these PCR products using DNA Clean and Concentrator-5 columns. We then quantified each one of the 24 libraries using UV absorbance as well as fluorescence using the QuantiFluor dsDNA Dye (Promega) along with a standard curve in a Molecular Device SpectraMax M5 plate reader. We then mixed the individual libraries at an equimolar ratio using the geometric mean of the concentrations determined by the two methods. We then submitted these for sequencing utilizing 5% of a NovaSeq 6000 2x150bp run, resulting in 44,421,626 total read pairs.

Analysis of HT-SELEX sequencing data

Demultiplexed sequencing reads were checked for quality using AfterQC and FastQC [3,4]. No important issues were noted for any samples. For all analyses except AptaSUITE, read pairs were merged using FLASH [5] and the N20- or N30-derived sequences were extracted used custom Pythons scripts which identified the SELEX library adapters in each sequence, then filtered out sequences <16 nt or >35 nt and stored the intervening sequence as FASTA files.

For MEME, random samples of sequence reads were obtained using Python scripts. All MEME searches in this text were performed using the RNA alphabet, background frequencies using a Markov-order of 1 (dinucleotide frequencies), and the differential enrichment objective function with version 5.0.2.

For AptaSUITE, the raw sequence reads were input directly to AptaPLEX, which handled identification of the SELEX library adapters and extraction of sequence. AptaCLUSTER was run with LSHDimension=25, EditDistance=5, LSHIterations=5, KmerSize=3 and KmerCutoffIterations=10000. AptaTRACE was run with KmerLength=6, FilterClusters=True, and Alpha=10.

1. Hall B, Arshad S, Seo K, Bowman C, Corley M, Jhaveri SD, et al. In vitro selection of RNA aptamers to a protein target by filter immobilization. Curr Protoc Mol Biol. 2009;Chapter 24: Unit 24.3.

2. Bouvet P. Identification of nucleic acid high-affinity binding sequences of proteins by SELEX. Methods Mol Biol. 2009;543: 139–150.

3. Andrews S, Others. FastQC: a quality control tool for high throughput sequence data. 2010;

4. Chen S, Huang T, Zhou Y, Han Y, Xu M, Gu J. AfterQC: automatic filtering, trimming, error removing and quality control for fastq data. BMC Bioinformatics. 2017;18: 80.

5. Magoc T, Salzberg SL. FLASH: fast length adjustment of short reads to improve genome assemblies. Bioinformatics. 2011;27: 2957–2963.

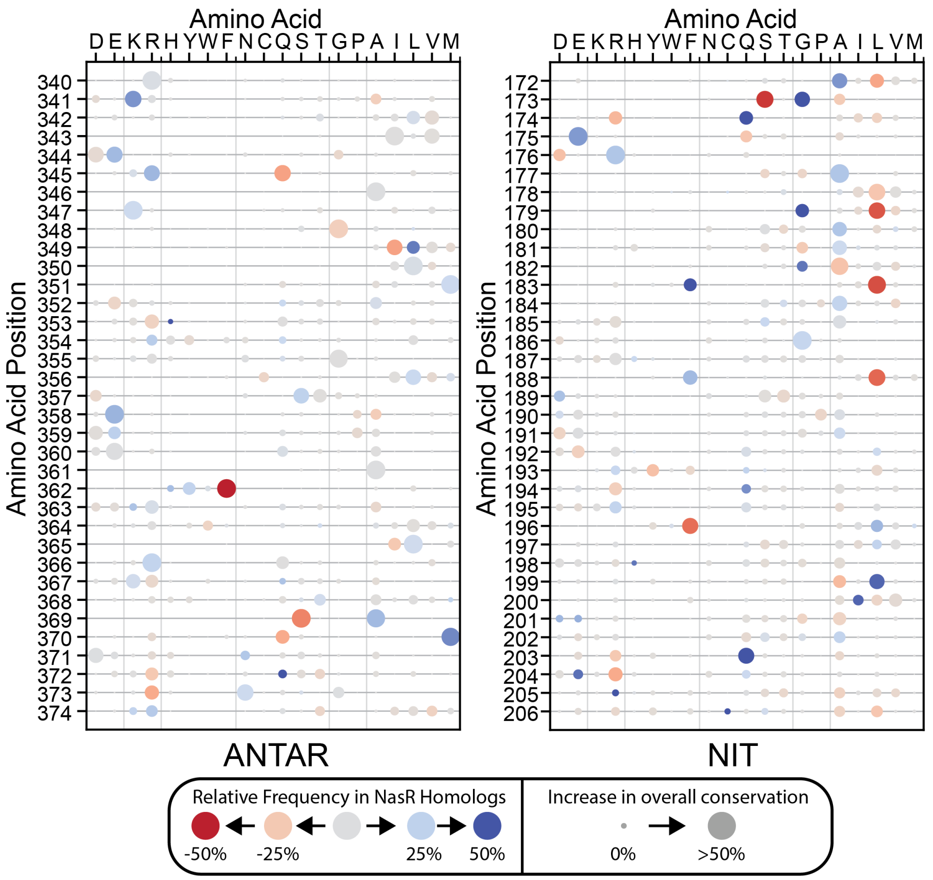

Figure S1: Compositional bias of NIT and ANTAR residues at an interdomain interface. (A) Representations of overall residue conservation and differential conservation between NasR homologs (~230 sequences) and other ANTAR domain-containing proteins (~8200 sequences) or other NIT domain-containing proteins (~3200 sequences). Each circle represents the presence of a particular amino acid at the alignment position represented by the NasR residue at each position along the left. The area of each circle is directly proportional to the fraction of all sequences at that position containing each amino acid, up to maximum size at 50% conservation among all sequences. The color of each circle represents the difference in proportion between NIT-ANTAR pair proteins (NasR homologs) against the overall alignment. Red represents amino acids that are less prevalent in NasR homologs compared to the overall alignment; blue represents those amino acids more prevalent in NasR homologs. Grey circles represent amino acids that are conserved at similar frequencies for NasR-like proteins and the overall alignment.

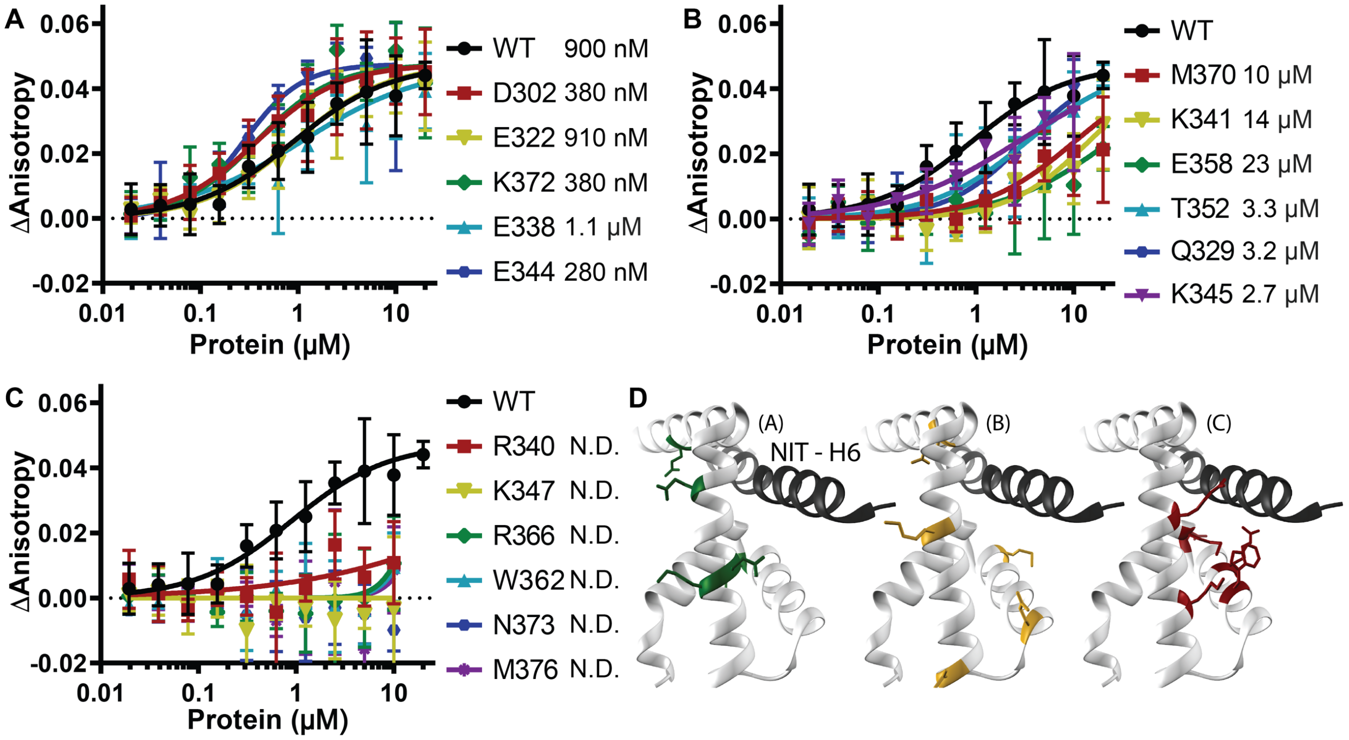

Figure S2: Site-directed mutagenesis of conserved ANTAR residues affects RNA binding. (A-C) Equilibrium saturation binding curves for nineteen alanine substitutions mutations for His10-MBP-NasR in the presence of 1 mM nitrate. Residues were grouped into (A) those with affinity better than or comparable to wild-type NasR, (B) those exhibiting greater than 2-fold reduction in binding affinity, and (C) those with negligible binding activity. For all panels, error bars represent the standard deviation of the anisotropy change of four replicate samples relative to a no-ligand sample. All datasets were fit to a Hill equation model. (D) Ribbon depictions of the ANTAR domain (PDB: 4AKK) with residues at positions corresponding to the alanine substitutions highlighted in color. Green residues correspond to mutations described in (A). Yellow residues correspond to mutations described in (B). Red residues correspond to mutations described in (C).

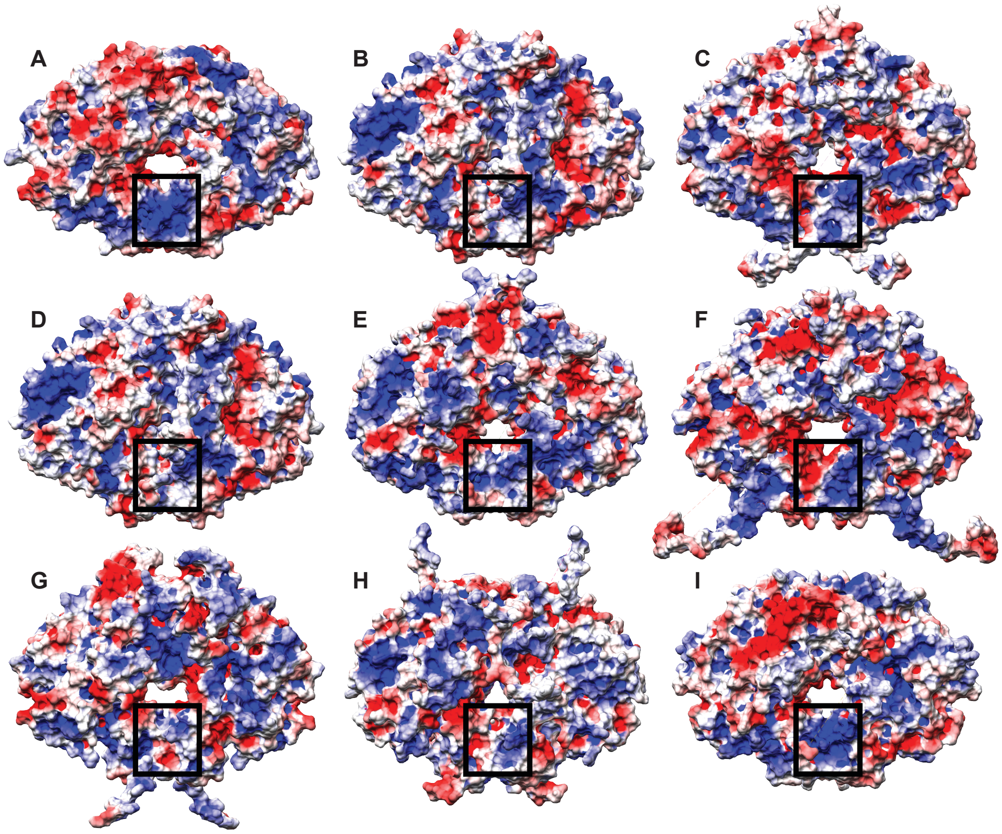

Figure S3: Electrostatic surface potential models of computationally predicted NasR homolog structures. Electrostatic surface potential for NasR (PDB: 4AKK) (A) or NasR homolog sequences (B-I) was predicted for structure models predicted by RaptorX using the CHARMM-GUI suite. The region corresponding to the positive patch of the ANTAR domain in *K. oxytoca* NasR is enclosed in a square for each structure. UniProt accession numbers for sequences used for structure prediction from each genome: (B) *Nitrincola nitratireducens* (W9V209) (C) *Acidovorax temperans* (A0A0D7KAF4) (D) *Halomonas sp. JB380* (A0A1R4HZH8) (E) *Erwinia tasmaniensis* (B2VKH7) (F) *Burkholderia cenocepacia* (B4E5J8) (G) *Pseudomonas stutzeri* (A4VRW3) (H) *Serratia ficaria* (A0A240C4M6) (I) *Klebsiella pneumoniae* (A6TAM9).

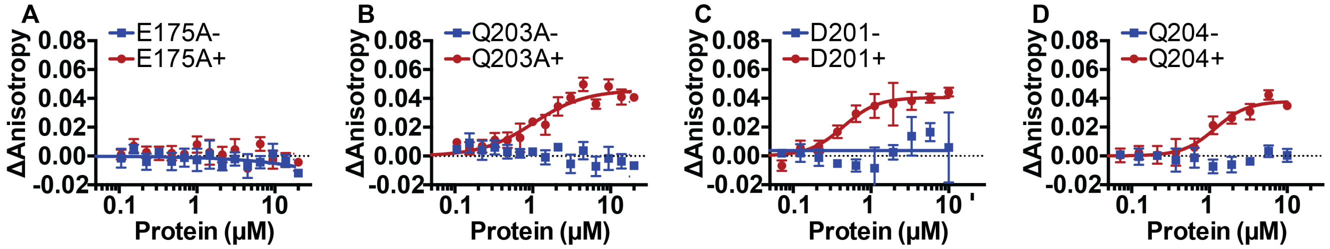

Figure S4: Assessment of helix 5 and helix residues for control of RNA binding activity. Equilibrium saturation binding curves for alanine substitution mutants of residues near the NIT-ANTAR interdomain interface or within helix 5, as described in the text. Blue data points represent binding curves performed in the absence of nitrate, while red data points represent binding curves with 1 mM nitrate. Error bars represent the standard deviation of the anisotropy change of four experiments relative to the no-ligand sample. The fit lines represent a Hill equation model.

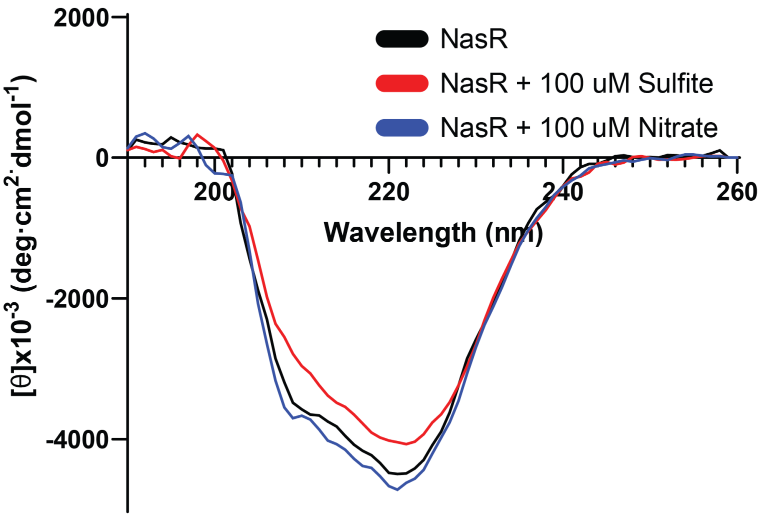

Sample	α-Helix	β-Sheet	Turn	Other
NasR	85.0%	0.0%	0.0%	15.0%
NasR + 100 μM Nitrate	86.5%	0.0%	0.0%	13.5%
NasR + 100 μM Sulfite	89.6%	0.0%	0.0%	10.4%

Figure S5: Circular dichroism spectroscopy of NasR proteins. NasR was purified as described and dialyzed into 10 mM sodium phosphate buffer (pH 7.5). Samples were then supplemented with either 100 μM KNO_3 or Na_2SO_3 at a final concentration of 10 μM and verified spectrophotometrically using extinction coefficients calculated in ProtParam. CD spectra were obtained on a Jasco J-1500 spectropolarimeter at 22˚C in a 1 mm quartz cuvette. Each spectra is an average of two scans taken at 50 nm/min from 260 to 190 nm in intervals of 1 nm. HT values never exceeded 600 V. Scans of samples containing NasR were buffer subtracted and converted to molar ellipticity before having their secondary structure elements analyzed in Spectra Manager's Multivariate SSE program.

Table S1: Relative representation of amino acids in alignments of NIT and ANTAR domains

For each position corresponding to a residue in *K. oxytoca* NasR in the multiple sequence alignment of NIT domains (positions 41-278) or ANTAR domains (positions 331-384), the percentage of sequences with each amino acid at that position is shown. These data were compared with that in Table S2 to generate the data for Figure 4.

Position in NasR	A	C	D	E	F	G	H	I	K	L	M	N	P	Q	R	S	T	V	W	Y
41	7.7%	0.0%	0.0%	0.0%	0.0%	23.1%	0.0%	0.0%	0.0%	7.7%	0.0%	0.0%	15.4%	0.0%	15.4%	0.0%	0.0%	30.8%	0.0%	0.0%
42	11.0%	0.1%	0.0%	0.0%	3.5%	0.0%	0.0%	1.6%	0.0%	58.2%	1.8%	0.0%	0.0%	0.0%	0.0%	0.1%	6.3%	13.7%	0.0%	3.6%
43	23.0%	0.0%	0.1%	0.2%	1.4%	1.1%	0.0%	15.3%	0.0%	7.8%	1.9%	0.3%	0.0%	0.0%	0.2%	3.5%	6.1%	38.5%	0.0%	0.6%
44	9.5%	0.0%	7.4%	2.8%	0.5%	3.0%	36.4%	1.0%	0.6%	3.7%	0.3%	4.1%	0.0%	9.0%	6.7%	3.0%	7.7%	2.2%	0.4%	1.7%
45	32.9%	0.3%	4.0%	21.3%	0.4%	2.8%	1.7%	0.1%	0.5%	2.3%	1.4%	3.7%	0.0%	7.5%	4.6%	8.3%	2.2%	5.1%	0.3%	0.5%
46	0.8%	0.1%	0.0%	0.0%	2.0%	0.1%	0.5%	6.6%	0.0%	77.2%	2.8%	0.0%	0.0%	0.4%	0.0%	0.2%	2.1%	7.1%	0.0%	0.0%
47	9.6%	0.0%	0.2%	3.0%	0.1%	1.9%	1.5%	1.3%	0.1%	2.8%	0.4%	0.1%	0.1%	74.9%	1.0%	1.0%	0.1%	0.3%	0.0%	1.6%
48	9.1%	0.5%	11.4%	8.1%	0.2%	1.1%	1.1%	2.3%	16.5%	3.9%	0.6%	6.3%	0.0%	11.6%	18.4%	1.6%	2.7%	4.4%	0.1%	0.3%
49	0.2%	0.0%	0.1%	96.1%	0.0%	0.1%	3.4%	0.0%	0.0%	0.0%	0.0%	0.0%	0.1%	0.1%	0.0%	0.1%	0.0%	0.0%	0.0%	0.0%
50	0.2%	0.0%	0.0%	0.0%	0.0%	0.3%	0.2%	0.0%	0.0%	0.6%	0.0%	0.0%	0.0%	0.1%	98.4%	0.0%	0.1%	0.0%	0.0%	0.0%
51	5.0%	0.0%	25.9%	0.9%	0.1%	32.2%	0.3%	0.6%	0.4%	2.8%	2.0%	0.5%	0.0%	1.9%	15.2%	5.0%	5.6%	1.0%	0.1%	0.5%
52	18.8%	0.1%	1.1%	5.5%	1.6%	0.9%	0.5%	3.0%	2.1%	30.6%	6.9%	0.5%	0.0%	9.9%	5.2%	2.3%	4.5%	4.1%	0.2%	2.2%
53	15.1%	0.1%	0.6%	1.6%	0.1%	0.8%	0.0%	0.4%	0.0%	1.1%	1.5%	0.2%	0.0%	0.1%	0.1%	44.1%	27.8%	6.4%	0.1%	0.0%
54	41.5%	0.2%	0.2%	0.1%	0.2%	1.8%	0.1%	2.1%	0.0%	10.7%	3.0%	7.6%	0.0%	0.4%	0.1%	5.4%	7.9%	18.6%	0.0%	0.1%
55	18.8%	0.1%	0.9%	5.9%	0.6%	25.3%	0.2%	5.2%	0.4%	7.7%	1.3%	0.4%	0.6%	3.4%	6.1%	4.8%	2.4%	14.3%	1.5%	0.2%
56	4.2%	0.3%	0.2%	0.8%	23.6%	0.4%	4.3%	0.7%	0.3%	9.3%	1.2%	0.4%	0.0%	2.4%	3.4%	1.2%	0.7%	4.9%	4.3%	37.4%
57	4.0%	0.1%	0.0%	0.1%	0.4%	0.3%	0.0%	14.6%	0.1%	63.6%	2.1%	0.1%	0.2%	0.8%	0.3%	0.7%	1.0%	10.5%	0.9%	0.2%
58	41.8%	1.8%	0.7%	0.7%	0.1%	22.1%	0.2%	0.4%	0.3%	8.1%	0.3%	1.4%	0.5%	0.3%	0.5%	10.5%	7.8%	2.5%	0.0%	0.1%
59	15.9%	0.3%	12.7%	3.0%	0.2%	11.0%	3.7%	0.4%	3.3%	1.6%	0.2%	5.9%	0.2%	3.6%	4.2%	28.8%	3.8%	1.0%	0.0%	0.1%
60	5.1%	0.0%	5.6%	4.0%	0.3%	11.2%	1.3%	0.2%	12.9%	0.3%	0.2%	3.9%	31.4%	6.6%	7.7%	4.6%	3.2%	1.2%	0.0%	0.5%
61	6.0%	0.2%	5.5%	3.0%	0.0%	62.2%	0.5%	0.0%	2.1%	0.3%	0.1%	4.6%	1.7%	1.3%	2.5%	6.4%	2.9%	0.3%	0.0%	0.2%
62	12.0%	0.4%	8.6%	11.0%	0.4%	4.8%	1.3%	0.4%	11.2%	2.0%	0.9%	4.4%	1.4%	15.1%	8.3%	5.2%	9.7%	3.1%	0.0%	0.0%
63	5.2%	0.0%	7.6%	4.4%	0.2%	6.0%	1.3%	0.2%	12.5%	2.7%	0.4%	4.6%	1.2%	5.9%	35.9%	6.2%	3.3%	1.7%	0.1%	0.5%
64	4.3%	0.4%	0.6%	0.4%	60.4%	1.3%	0.7%	0.6%	2.6%	5.3%	3.2%	0.5%	4.4%	1.3%	4.2%	1.2%	1.1%	0.2%	1.6%	5.6%
65	16.0%	0.1%	4.3%	4.2%	0.8%	5.7%	0.7%	1.0%	9.6%	14.0%	0.6%	1.2%	4.5%	4.8%	16.4%	4.9%	6.7%	4.0%	0.0%	0.4%
66	23.9%	0.0%	14.5%	6.9%	0.2%	7.3%	0.4%	0.5%	3.9%	1.3%	0.0%	4.4%	6.6%	3.7%	3.0%	10.5%	10.9%	1.8%	0.0%	0.3%
67	22.0%	0.0%	16.0%	17.1%	0.1%	2.3%	0.2%	1.1%	5.0%	2.5%	0.5%	4.5%	5.6%	6.8%	6.3%	3.7%	3.5%	2.7%	0.0%	0.1%
68	0.9%	0.2%	0.3%	0.1%	2.4%	0.3%	0.2%	3.7%	0.1%	55.0%	3.0%	0.0%	0.3%	0.3%	6.0%	3.6%	1.3%	18.2%	0.1%	4.0%
69	12.8%	0.0%	8.8%	11.1%	0.1%	2.5%	1.5%	1.8%	10.0%	4.8%	0.9%	4.2%	4.4%	7.1%	12.8%	3.8%	7.9%	5.5%	0.0%	0.1%
70	22.0%	0.1%	6.9%	13.2%	0.1%	8.1%	0.5%	0.4%	8.3%	2.0%	0.3%	2.6%	1.9%	11.7%	9.4%	5.2%	5.8%	1.3%	0.0%	0.1%
71	15.2%	0.2%	1.3%	2.4%	0.5%	0.6%	1.6%	1.5%	2.1%	6.4%	0.7%	0.2%	2.1%	39.5%	10.4%	4.7%	3.9%	4.0%	0.2%	2.4%
72	5.4%	0.1%	0.1%	1.8%	0.8%	1.3%	1.5%	3.8%	0.8%	1.3%	0.8%	0.8%	0.0%	8.5%	58.5%	5.2%	3.0%	3.4%	0.4%	2.7%
73	26.4%	0.0%	6.2%	9.1%	0.2%	4.2%	0.3%	1.2%	9.8%	2.8%	0.1%	2.4%	1.5%	10.8%	11.5%	4.2%	6.9%	2.3%	0.0%	0.1%
74	22.8%	0.1%	5.0%	9.1%	0.1%	1.4%	0.8%	2.3%	11.2%	6.0%	0.3%	2.6%	0.6%	5.6%	21.4%	2.8%	4.2%	3.6%	0.0%	0.1%
75	4.4%	1.2%	0.0%	0.1%	0.1%	0.3%	0.0%	1.6%	0.0%	1.6%	0.1%	0.3%	0.0%	0.1%	0.1%	7.4%	61.3%	21.3%	0.0%	0.0%

76	1.2%	0.1%	83.5%	2.0%	0.0%	0.5%	0.2%	0.2%	0.4%	0.8%	0.1%	5.2%	0.1%	2.2%	1.0%	1.1%	1.0%	0.5%	0.0%	0.0%
77	18.5%	0.0%	5.7%	12.0%	0.1%	2.2%	1.1%	0.6%	8.7%	2.8%	0.3%	1.6%	0.5%	11.0%	24.8%	3.6%	4.9%	1.3%	0.0%	0.2%
78	54.1%	0.2%	2.1%	3.4%	0.4%	0.9%	1.0%	1.3%	3.9%	4.8%	0.8%	0.5%	0.0%	9.0%	5.0%	5.3%	3.2%	3.7%	0.1%	0.3%
79	12.9%	0.1%	0.1%	4.7%	1.8%	0.5%	0.7%	15.1%	4.2%	18.9%	2.1%	0.3%	0.0%	2.0%	7.2%	1.2%	1.5%	24.5%	0.4%	1.8%
80	29.1%	0.0%	12.2%	10.4%	0.2%	5.8%	0.8%	0.6%	7.2%	1.5%	0.1%	3.4%	0.2%	7.4%	7.2%	5.1%	6.7%	2.2%	0.0%	0.0%
81	22.2%	0.0%	6.9%	18.5%	0.1%	3.2%	1.1%	1.1%	8.4%	1.9%	0.2%	5.8%	2.6%	5.8%	9.6%	4.0%	5.8%	2.8%	0.0%	0.1%
82	2.5%	0.0%	0.0%	0.1%	28.6%	0.0%	0.1%	6.0%	0.1%	36.0%	5.8%	0.0%	0.0%	0.1%	0.4%	0.1%	0.8%	11.3%	0.6%	7.6%
83	6.4%	0.2%	6.3%	7.1%	0.5%	1.5%	1.5%	1.2%	6.2%	4.7%	0.8%	3.2%	0.5%	8.1%	39.6%	4.8%	5.2%	1.6%	0.2%	0.4%
84	29.4%	0.0%	6.9%	10.0%	0.1%	3.9%	0.9%	0.4%	4.1%	5.5%	0.0%	3.0%	1.2%	8.8%	10.6%	8.7%	5.0%	0.9%	0.1%	0.3%
85	24.6%	0.8%	3.5%	4.5%	4.6%	9.5%	1.7%	2.1%	1.5%	7.4%	0.6%	1.0%	0.2%	3.6%	5.2%	7.5%	9.8%	9.2%	0.7%	2.2%
86	17.9%	0.1%	4.3%	2.7%	4.6%	4.5%	0.4%	5.7%	2.9%	28.9%	1.6%	0.5%	0.4%	2.5%	7.5%	3.6%	3.1%	7.0%	0.6%	1.3%
87	19.8%	0.1%	16.6%	7.6%	0.2%	8.8%	1.0%	1.1%	4.5%	2.9%	0.4%	4.4%	3.1%	4.8%	5.2%	12.2%	4.9%	2.5%	0.0%	0.1%
88	11.1%	0.0%	19.5%	13.8%	0.2%	7.9%	1.3%	0.4%	4.9%	1.9%	0.4%	5.5%	1.8%	6.7%	8.3%	9.7%	4.9%	1.2%	0.4%	0.2%
89	9.1%	0.1%	1.7%	1.3%	4.0%	1.9%	0.9%	15.7%	0.6%	22.6%	10.1%	1.2%	0.9%	1.8%	1.1%	3.0%	4.2%	19.0%	0.2%	0.4%
90	6.7%	0.0%	19.9%	9.3%	0.2%	4.9%	3.3%	0.4%	3.2%	2.0%	0.4%	4.8%	5.1%	6.3%	21.2%	6.0%	3.8%	1.6%	0.0%	0.9%
91	19.6%	0.1%	2.5%	4.3%	2.0%	3.7%	1.5%	3.0%	3.8%	14.0%	1.2%	1.2%	7.8%	4.8%	3.6%	9.6%	8.7%	6.3%	0.8%	1.6%
92	12.0%	0.0%	23.8%	9.2%	0.7%	9.2%	3.2%	0.6%	2.4%	2.6%	0.7%	5.7%	5.4%	2.9%	3.4%	10.3%	4.9%	1.8%	0.2%	1.1%
93	9.3%	0.0%	3.4%	1.0%	3.9%	3.9%	1.2%	9.3%	0.4%	23.2%	2.8%	1.6%	5.1%	3.0%	0.2%	4.6%	5.6%	19.6%	0.1%	1.6%
94	19.5%	0.3%	5.3%	8.8%	0.3%	8.1%	1.8%	0.8%	5.9%	3.2%	0.7%	2.9%	2.3%	6.9%	16.9%	7.8%	3.3%	4.3%	0.3%	0.6%
95	16.8%	0.0%	14.2%	11.8%	0.3%	5.9%	0.9%	0.6%	2.4%	0.8%	0.5%	3.8%	2.6%	6.2%	14.0%	10.8%	6.1%	1.9%	0.2%	0.2%
96	9.9%	0.1%	3.1%	3.7%	0.2%	2.3%	1.9%	1.1%	4.6%	4.2%	0.4%	4.0%	1.6%	5.2%	42.6%	7.7%	4.8%	1.5%	0.2%	1.0%
97	4.7%	0.0%	0.2%	0.1%	4.9%	0.7%	0.3%	10.0%	0.5%	50.9%	4.2%	0.7%	0.3%	0.4%	1.2%	0.6%	1.7%	12.8%	1.8%	4.0%
98	2.3%	4.7%	0.4%	1.3%	20.5%	2.1%	0.4%	2.6%	2.6%	32.0%	1.1%	0.4%	6.8%	1.7%	1.9%	3.2%	4.3%	2.8%	0.2%	8.7%
99	11.9%	0.0%	15.9%	8.5%	0.2%	4.1%	0.9%	1.0%	5.0%	2.8%	0.3%	7.8%	0.8%	7.7%	7.8%	9.4%	8.8%	5.0%	0.2%	2.1%
100	18.0%	0.3%	7.7%	9.5%	0.1%	5.8%	1.0%	1.1%	4.6%	6.2%	0.4%	2.7%	1.2%	8.8%	12.5%	9.2%	6.3%	3.7%	0.8%	0.1%
101	13.4%	0.1%	0.4%	0.1%	14.2%	1.5%	0.0%	19.4%	0.2%	23.9%	3.0%	0.2%	0.1%	0.2%	0.1%	1.7%	2.1%	18.6%	0.1%	0.7%
102	13.2%	0.1%	6.5%	5.3%	1.7%	3.2%	0.8%	3.1%	3.6%	18.9%	1.4%	3.6%	0.2%	4.6%	15.6%	4.1%	4.0%	9.6%	0.0%	0.7%
103	12.1%	0.3%	11.4%	9.2%	0.6%	8.8%	1.4%	0.8%	7.5%	2.2%	0.9%	4.3%	0.4%	7.8%	11.7%	8.9%	8.4%	1.5%	0.5%	1.4%
104	48.9%	0.4%	1.1%	0.8%	1.1%	1.9%	0.4%	1.9%	0.0%	1.9%	0.8%	0.0%	0.8%	0.0%	0.0%	3.0%	1.9%	35.3%	0.0%	0.0%
105	1.4%	0.0%	1.8%	1.1%	1.8%	0.7%	0.4%	1.1%	1.4%	59.9%	1.8%	1.1%	0.0%	2.1%	1.4%	3.9%	2.5%	10.6%	6.3%	1.1%
106	2.8%	0.3%	2.4%	1.0%	1.0%	1.4%	30.3%	0.7%	1.4%	2.8%	0.3%	4.1%	4.8%	21.4%	1.4%	2.1%	2.8%	1.0%	3.4%	14.5%
107	20.9%	0.3%	9.3%	7.3%	0.6%	9.3%	3.3%	0.4%	2.7%	4.2%	0.6%	2.5%	0.6%	13.1%	9.3%	8.2%	3.0%	3.3%	0.4%	0.7%
108	4.4%	0.0%	0.1%	7.0%	8.4%	0.4%	0.1%	4.5%	0.3%	58.1%	2.6%	0.0%	0.1%	0.4%	0.5%	0.5%	1.4%	6.4%	3.2%	1.6%
109	10.9%	0.0%	27.7%	12.1%	0.0%	11.2%	1.1%	0.4%	3.5%	0.6%	0.2%	6.1%	6.1%	4.7%	3.1%	7.3%	3.8%	0.7%	0.0%	0.3%
110	10.4%	0.0%	7.4%	7.6%	0.2%	17.7%	1.2%	0.5%	9.2%	1.4%	0.6%	3.7%	0.4%	12.3%	11.6%	7.0%	7.3%	0.9%	0.0%	0.6%
111	0.2%	0.0%	0.0%	0.0%	0.3%	0.0%	0.0%	13.8%	0.8%	76.0%	1.5%	0.1%	0.0%	0.2%	0.7%	0.1%	0.6%	5.4%	0.1%	0.0%
112	16.4%	0.0%	10.5%	6.9%	0.1%	7.4%	0.4%	0.7%	3.4%	0.4%	0.0%	6.0%	23.0%	5.0%	4.6%	7.1%	6.6%	1.4%	0.0%	0.1%
113	22.0%	0.0%	9.3%	10.9%	0.2%	8.0%	0.7%	0.8%	5.4%	1.2%	0.1%	2.8%	3.5%	7.1%	3.3%	14.5%	7.3%	2.1%	0.0%	0.7%
114	2.0%	0.0%	0.1%	0.5%	4.4%	1.3%	2.1%	18.8%	1.3%	42.2%	2.6%	0.5%	0.1%	2.1%	1.4%	0.4%	5.8%	12.5%	0.3%	1.4%
115	0.6%	0.1%	0.0%	0.5%	0.0%	0.1%	0.3%	0.1%	0.5%	0.1%	0.0%	0.1%	0.0%	0.6%	96.1%	0.4%	0.3%	0.1%	0.0%	0.0%
116	15.3%	0.1%	6.1%	9.7%	0.0%	6.0%	1.1%	0.4%	8.8%	1.0%	0.2%	3.8%	1.1%	11.3%	15.6%	11.2%	6.7%	1.2%	0.1%	0.3%
117	17.7%	0.1%	5.6%	5.0%	1.1%	6.5%	0.8%	0.6%	6.8%	2.4%	0.2%	5.1%	0.6%	15.8%	11.5%	11.2%	5.9%	2.9%	0.0%	0.2%
118	26.9%	0.0%	0.3%	0.0%	0.4%	0.2%	0.0%	18.7%	0.1%	4.0%	0.8%	0.2%	0.1%	0.1%	0.2%	0.7%	2.3%	45.0%	0.0%	0.0%
119	5.1%	0.0%	42.5%	10.5%	0.8%	3.8%	0.5%	0.9%	1.4%	12.9%	0.3%	3.1%	1.0%	1.7%	3.9%	5.6%	4.2%	1.5%	0.1%	0.3%
120	17.7%	0.1%	10.6%	6.9%	0.3%	6.9%	0.9%	0.3%	3.6%	1.0%	0.2%	7.7%	1.1%	5.9%	6.5%	12.8%	16.2%	1.0%	0.1%	0.3%
121	2.1%	0.2%	1.7%	1.2%	2.0%	31.6%	4.0%	0.2%	3.4%	15.0%	0.5%	3.2%	3.8%	6.7%	20.8%	2.0%	1.2%	0.5%	0.0%	0.1%

122	10.0%	0.1%	6.8%	5.0%	0.1%	4.3%	1.2%	0.7%	12.0%	1.4%	0.3%	4.5%	1.7%	8.3%	9.2%	18.0%	15.3%	1.1%	0.1%	0.1%
123	10.6%	0.4%	2.0%	1.2%	1.3%	6.0%	0.1%	18.4%	1.0%	19.1%	4.5%	1.5%	1.7%	1.3%	1.2%	7.4%	7.0%	14.5%	0.4%	0.4%
124	5.2%	0.0%	16.2%	3.3%	0.3%	7.2%	0.4%	0.3%	3.3%	0.5%	0.2%	8.5%	11.5%	2.0%	1.6%	18.4%	19.2%	1.0%	0.0%	0.9%
125	21.9%	0.0%	3.0%	1.4%	1.7%	4.0%	0.7%	3.0%	1.1%	4.9%	0.4%	1.3%	14.9%	3.5%	18.8%	4.4%	3.1%	5.1%	6.2%	0.4%
126	14.3%	0.0%	6.2%	9.8%	0.8%	2.0%	0.7%	1.3%	2.5%	5.1%	1.1%	2.5%	4.9%	6.0%	2.2%	9.7%	10.1%	20.2%	0.1%	0.6%
127	12.6%	0.0%	12.7%	23.3%	0.1%	4.4%	0.5%	0.4%	4.2%	1.1%	0.3%	2.0%	2.2%	23.4%	3.9%	3.2%	4.2%	1.2%	0.0%	0.3%
128	37.5%	0.3%	0.3%	1.8%	0.3%	0.9%	0.1%	4.7%	0.1%	2.4%	1.6%	1.9%	1.3%	0.6%	0.3%	7.0%	23.0%	15.4%	0.2%	0.3%
129	5.4%	0.0%	0.3%	7.2%	10.9%	1.5%	5.0%	10.5%	0.4%	19.0%	4.6%	1.0%	0.0%	1.5%	0.6%	1.4%	6.5%	15.6%	0.0%	8.7%
130	15.4%	0.0%	13.2%	18.4%	1.3%	9.4%	0.7%	0.5%	3.7%	2.6%	0.4%	4.9%	0.3%	7.6%	6.7%	5.7%	7.5%	1.5%	0.0%	0.2%
131	20.9%	0.1%	1.9%	2.9%	9.4%	9.8%	1.0%	0.7%	1.6%	5.5%	0.5%	2.0%	0.6%	6.9%	8.6%	5.8%	4.1%	1.6%	2.9%	13.1%
132	0.1%	0.0%	0.0%	0.0%	8.6%	0.0%	3.2%	0.1%	0.0%	0.8%	0.1%	0.0%	0.0%	0.0%	0.0%	0.2%	0.1%	0.1%	0.1%	86.4%
133	2.7%	0.7%	3.1%	0.8%	0.1%	4.5%	4.7%	0.6%	0.2%	0.2%	0.1%	16.3%	0.0%	0.8%	1.8%	23.0%	34.2%	5.9%	0.0%	0.0%
134	12.8%	0.1%	8.3%	11.1%	0.2%	11.3%	1.3%	1.5%	5.8%	2.0%	0.2%	3.7%	1.7%	8.4%	15.1%	8.2%	5.9%	2.2%	0.1%	0.1%
135	8.3%	0.1%	0.1%	0.2%	0.9%	0.2%	0.2%	16.4%	0.1%	36.8%	5.0%	0.3%	0.2%	0.2%	0.8%	1.7%	8.1%	20.4%	0.1%	0.2%
136	3.7%	0.1%	0.6%	1.0%	0.7%	0.1%	0.2%	52.7%	0.0%	7.9%	0.6%	5.8%	0.2%	4.0%	0.0%	1.2%	2.5%	18.5%	0.0%	0.2%
137	14.4%	0.0%	22.5%	9.1%	0.1%	6.3%	7.6%	0.3%	2.4%	0.8%	0.6%	8.3%	0.2%	3.2%	4.9%	7.2%	10.6%	1.2%	0.0%	0.2%
138	15.9%	0.1%	9.8%	5.9%	0.6%	6.8%	3.1%	0.5%	3.8%	2.0%	0.5%	3.7%	13.3%	6.0%	4.1%	14.5%	5.6%	2.5%	0.0%	1.1%
139	8.8%	4.9%	1.0%	0.5%	3.1%	4.3%	0.5%	4.1%	0.2%	58.2%	2.9%	0.3%	0.1%	2.7%	0.2%	1.5%	1.5%	4.2%	0.3%	0.8%
140	1.4%	0.0%	0.6%	0.1%	10.5%	1.5%	5.3%	15.2%	0.1%	40.8%	7.7%	1.0%	0.1%	1.0%	0.4%	1.1%	2.7%	4.9%	0.1%	5.6%
141	17.4%	0.0%	15.3%	10.5%	0.6%	8.1%	4.9%	0.7%	2.1%	1.8%	0.3%	5.7%	0.2%	5.6%	7.6%	12.2%	4.4%	1.7%	0.0%	0.8%
142	5.9%	0.1%	0.6%	0.4%	14.0%	1.7%	0.2%	8.1%	0.1%	37.2%	2.4%	0.9%	0.5%	0.7%	1.0%	1.5%	2.8%	18.1%	1.3%	2.5%
143	12.7%	0.3%	4.7%	1.1%	7.6%	6.7%	1.9%	8.2%	0.3%	11.0%	1.1%	2.2%	0.5%	1.2%	4.3%	12.7%	5.3%	13.4%	0.2%	4.5%
144	1.5%	0.0%	0.4%	1.5%	67.6%	2.5%	0.7%	1.5%	0.0%	4.4%	0.0%	0.4%	5.5%	0.4%	0.4%	2.5%	2.2%	1.8%	0.4%	6.5%
145	9.4%	0.0%	15.4%	15.5%	0.9%	9.5%	0.6%	0.6%	1.1%	1.7%	0.9%	12.3%	1.4%	12.4%	6.0%	6.1%	4.1%	1.2%	0.0%	0.7%
146	11.0%	0.1%	0.8%	1.3%	1.5%	0.9%	0.1%	11.4%	0.1%	44.1%	10.9%	0.4%	0.1%	2.6%	0.2%	2.0%	3.1%	9.3%	0.0%	0.2%
147	38.3%	0.0%	1.8%	1.5%	1.1%	13.4%	3.7%	2.9%	0.2%	2.8%	0.5%	2.2%	2.3%	1.1%	1.7%	11.0%	9.5%	5.4%	0.3%	0.2%
148	14.7%	0.1%	13.1%	10.5%	0.5%	15.5%	2.9%	0.8%	3.8%	1.9%	0.3%	3.8%	0.7%	10.8%	7.1%	7.1%	4.5%	1.4%	0.0%	0.3%
149	12.8%	0.0%	3.2%	3.8%	1.0%	8.5%	2.5%	6.0%	2.4%	13.2%	1.6%	2.9%	1.2%	5.9%	5.4%	10.4%	9.4%	8.7%	0.0%	1.1%
150	15.0%	0.2%	1.0%	1.1%	1.6%	2.2%	0.4%	8.6%	0.5%	12.2%	3.1%	1.3%	1.6%	1.0%	1.0%	12.8%	21.9%	14.3%	0.0%	0.2%
151	5.8%	0.1%	17.8%	5.6%	0.7%	7.6%	1.3%	1.0%	1.8%	2.6%	0.2%	5.4%	15.1%	6.8%	2.8%	14.6%	7.0%	3.1%	0.0%	0.6%
152	3.7%	0.1%	49.2%	4.8%	0.3%	2.3%	1.7%	0.2%	1.0%	0.7%	0.1%	18.7%	4.9%	1.5%	0.9%	6.0%	2.9%	0.6%	0.0%	0.3%
153	15.3%	0.0%	3.3%	3.9%	0.0%	15.9%	1.2%	1.0%	2.0%	1.9%	0.2%	1.8%	21.5%	2.4%	9.2%	8.2%	4.8%	7.1%	0.0%	0.1%
154	13.7%	0.0%	15.5%	27.6%	0.1%	6.5%	0.9%	0.4%	1.9%	0.8%	0.1%	8.3%	1.7%	6.3%	4.7%	5.5%	4.1%	1.6%	0.1%	0.1%
155	4.5%	0.0%	0.5%	0.5%	0.8%	3.8%	0.0%	23.5%	0.1%	34.0%	11.4%	0.6%	0.2%	0.9%	0.4%	1.6%	2.6%	14.4%	0.0%	0.2%
156	19.7%	0.0%	7.9%	1.4%	0.9%	7.8%	0.4%	8.2%	1.1%	2.9%	0.6%	3.2%	0.7%	1.3%	2.7%	16.5%	18.0%	4.7%	0.1%	1.8%
157	7.3%	0.0%	5.8%	2.8%	0.4%	8.8%	1.4%	0.7%	10.7%	1.4%	0.2%	3.4%	1.7%	9.8%	22.8%	17.2%	3.6%	1.3%	0.1%	0.8%
158	13.0%	0.2%	9.5%	9.3%	0.3%	6.1%	1.3%	0.9%	1.7%	10.1%	1.5%	2.4%	1.2%	19.3%	9.5%	6.0%	5.7%	1.4%	0.2%	0.4%
159	18.7%	0.0%	0.1%	0.4%	0.9%	15.2%	0.6%	7.0%	0.2%	22.8%	3.8%	0.6%	0.5%	0.7%	2.5%	5.1%	12.4%	6.1%	0.4%	2.2%
160	6.8%	0.0%	2.8%	2.0%	0.7%	4.2%	2.3%	4.0%	0.9%	6.9%	1.5%	3.2%	0.3%	5.3%	37.2%	4.6%	5.4%	9.4%	0.0%	2.4%
161	57.6%	0.4%	0.5%	0.7%	0.2%	4.7%	0.2%	0.8%	0.1%	1.1%	1.1%	1.2%	3.8%	0.6%	0.1%	7.6%	11.6%	7.1%	0.0%	0.4%
162	6.5%	0.0%	0.1%	0.1%	5.6%	1.0%	0.7%	3.1%	0.3%	46.5%	3.9%	0.3%	1.1%	0.3%	0.6%	1.4%	1.9%	14.6%	0.1%	11.8%
163	17.3%	0.1%	1.7%	1.6%	6.0%	2.0%	3.1%	5.9%	2.7%	10.4%	0.9%	2.8%	1.0%	3.6%	2.0%	4.4%	7.1%	15.8%	0.0%	11.5%
164	33.2%	0.0%	6.1%	11.9%	0.6%	5.0%	3.5%	0.4%	0.5%	2.2%	1.0%	14.1%	1.3%	4.2%	1.4%	9.3%	3.4%	0.6%	0.0%	1.2%
165	1.9%	0.0%	0.0%	0.0%	23.6%	0.3%	0.0%	11.4%	0.0%	46.7%	3.6%	0.1%	0.1%	0.2%	0.0%	0.4%	0.8%	9.9%	0.1%	0.8%
166	14.5%	0.0%	4.4%	3.7%	4.9%	7.4%	0.1%	3.6%	0.1%	16.2%	7.8%	1.2%	2.2%	1.8%	0.3%	20.6%	4.1%	5.7%	0.1%	1.2%
167	3.6%	0.3%	1.9%	7.4%	0.4%	1.3%	4.3%	0.5%	3.0%	2.7%	1.5%	4.3%	0.3%	18.4%	35.9%	5.3%	3.7%	1.5%	2.0%	1.9%

168	58.7%	0.1%	0.1%	0.7%	0.4%	10.0%	0.1%	3.0%	0.1%	4.5%	2.6%	0.2%	0.0%	0.9%	0.1%	6.7%	5.1%	6.2%	0.1%	0.3%
169	8.0%	0.1%	1.1%	1.4%	0.0%	3.9%	0.2%	1.4%	50.0%	0.7%	1.0%	1.3%	0.0%	2.8%	15.1%	2.4%	3.9%	6.6%	0.1%	0.1%
170	7.5%	0.0%	7.5%	77.8%	0.1%	1.3%	0.6%	0.1%	0.1%	0.2%	0.1%	0.6%	0.0%	3.2%	0.1%	0.7%	0.2%	0.1%	0.0%	0.0%
171	17.5%	0.9%	0.6%	4.4%	5.9%	1.6%	1.5%	0.8%	1.3%	17.1%	5.4%	2.0%	0.0%	12.2%	7.9%	5.8%	1.4%	0.9%	1.2%	11.5%
172	32.9%	0.6%	0.1%	3.5%	1.4%	0.7%	0.2%	5.2%	0.2%	27.7%	5.5%	1.0%	0.0%	1.2%	0.1%	4.6%	3.8%	10.6%	0.0%	0.8%
173	18.0%	0.0%	0.8%	0.6%	0.0%	33.5%	0.3%	0.1%	0.0%	0.9%	0.0%	1.2%	0.0%	0.7%	0.6%	41.2%	1.2%	0.2%	0.1%	0.5%
174	6.4%	0.0%	0.3%	2.4%	0.2%	0.5%	0.2%	12.1%	1.6%	14.9%	1.5%	0.3%	0.0%	26.4%	25.2%	0.7%	0.9%	5.2%	0.1%	1.1%
175	9.7%	0.5%	0.2%	50.7%	0.0%	0.8%	0.1%	2.2%	1.2%	1.4%	0.7%	0.1%	0.0%	20.2%	1.6%	3.2%	5.0%	2.1%	0.0%	0.3%
176	1.5%	0.0%	21.5%	1.3%	0.1%	0.6%	1.7%	0.1%	0.4%	0.1%	0.1%	2.3%	0.0%	4.9%	62.5%	0.9%	1.4%	0.1%	0.2%	0.2%
177	62.8%	0.0%	1.2%	0.6%	0.2%	12.6%	0.0%	0.7%	0.0%	0.6%	0.5%	0.2%	0.2%	0.5%	0.5%	12.3%	3.6%	3.4%	0.0%	0.1%
178	5.0%	1.1%	0.3%	0.7%	2.1%	0.2%	0.3%	17.1%	0.6%	39.3%	2.9%	0.4%	0.1%	1.7%	1.4%	1.3%	4.8%	18.4%	0.9%	1.4%
179	5.1%	0.0%	0.0%	0.4%	1.1%	25.6%	0.1%	9.5%	0.0%	38.7%	3.7%	0.1%	0.0%	0.0%	0.0%	0.3%	0.5%	12.7%	0.0%	2.3%
180	30.0%	0.1%	0.7%	0.9%	1.0%	7.1%	1.4%	1.3%	0.4%	10.5%	4.6%	5.9%	0.1%	1.5%	1.9%	15.1%	12.0%	4.4%	0.0%	1.3%
181	30.3%	0.0%	0.7%	1.4%	0.5%	19.1%	8.0%	2.9%	0.6%	4.4%	0.9%	4.3%	0.3%	2.7%	3.8%	11.6%	3.6%	3.3%	0.1%	1.6%
182	43.2%	0.1%	0.0%	0.1%	1.7%	16.6%	0.0%	4.3%	0.0%	10.8%	1.8%	0.2%	0.1%	0.1%	0.1%	3.6%	4.1%	11.7%	0.1%	1.5%
183	4.3%	0.1%	0.1%	0.3%	23.6%	1.8%	0.4%	7.0%	0.0%	45.2%	0.9%	0.4%	0.2%	1.2%	4.8%	0.6%	0.5%	3.8%	2.5%	2.3%
184	33.2%	0.1%	1.0%	1.0%	0.4%	8.0%	0.2%	4.3%	0.8%	4.3%	0.4%	2.7%	9.1%	1.3%	1.7%	10.5%	7.9%	12.9%	0.0%	0.2%
185	25.1%	0.1%	2.5%	4.0%	0.1%	4.5%	0.9%	0.5%	7.9%	1.8%	0.3%	3.7%	0.6%	5.3%	19.5%	12.5%	6.8%	3.7%	0.0%	0.2%
186	2.9%	0.1%	10.0%	1.1%	0.1%	60.0%	0.7%	0.1%	4.4%	0.2%	0.1%	5.6%	1.7%	1.8%	5.6%	4.5%	1.0%	0.1%	0.0%	0.1%
187	9.8%	0.0%	4.1%	9.2%	0.6%	8.3%	5.1%	0.7%	8.7%	1.0%	0.1%	3.4%	1.7%	7.3%	21.4%	8.1%	7.0%	1.6%	0.3%	1.7%
188	2.4%	1.0%	0.2%	0.3%	29.6%	0.7%	0.0%	6.3%	0.1%	40.0%	6.7%	0.3%	1.1%	0.6%	0.4%	2.2%	1.1%	4.6%	0.4%	1.9%
189	6.9%	0.0%	17.5%	4.3%	0.3%	6.5%	0.4%	0.1%	2.9%	1.4%	0.1%	5.0%	2.8%	1.7%	1.8%	22.9%	23.8%	1.1%	0.2%	0.1%
190	16.5%	0.0%	9.1%	13.8%	0.7%	7.3%	1.1%	0.5%	2.8%	2.9%	0.4%	2.5%	19.7%	4.1%	5.6%	5.9%	4.9%	1.7%	0.1%	0.6%
191	18.5%	0.0%	21.3%	17.2%	0.2%	11.3%	0.9%	0.7%	2.9%	1.0%	0.2%	2.2%	3.8%	4.7%	2.7%	5.0%	4.9%	2.3%	0.1%	0.2%
192	4.9%	0.1%	10.7%	23.2%	0.7%	2.6%	1.5%	1.4%	0.8%	9.8%	2.8%	2.7%	0.4%	14.1%	14.1%	3.8%	3.6%	2.0%	0.2%	0.4%
193	2.6%	0.4%	0.0%	1.1%	11.7%	0.4%	6.6%	4.1%	2.2%	16.8%	1.4%	0.3%	0.2%	6.0%	12.8%	1.9%	3.1%	4.2%	2.4%	21.8%
194	13.9%	0.0%	4.6%	6.8%	0.6%	3.0%	1.7%	3.5%	2.4%	3.8%	0.6%	2.9%	0.3%	12.6%	24.1%	7.1%	6.1%	5.8%	0.1%	0.3%
195	11.6%	0.0%	7.7%	9.4%	0.3%	1.7%	2.1%	0.8%	8.6%	7.9%	1.1%	2.0%	0.4%	14.3%	21.0%	4.3%	5.0%	1.3%	0.6%	0.3%
196	5.6%	0.0%	0.2%	0.2%	36.7%	4.4%	0.8%	8.8%	0.1%	21.3%	3.0%	0.0%	0.0%	0.2%	0.1%	0.0%	0.8%	7.1%	2.8%	7.9%
197	11.4%	0.3%	0.9%	3.3%	0.8%	2.4%	0.9%	11.2%	1.6%	13.3%	2.0%	2.0%	0.2%	4.7%	4.8%	13.4%	11.7%	14.4%	0.1%	0.8%
198	17.6%	0.0%	11.9%	8.4%	0.8%	10.2%	4.0%	0.4%	3.2%	1.6%	0.4%	4.9%	0.7%	8.1%	8.4%	11.0%	6.2%	1.2%	0.1%	1.0%
199	23.4%	0.5%	2.7%	3.1%	2.2%	0.7%	0.9%	2.9%	0.8%	32.6%	1.3%	2.7%	0.1%	3.5%	4.4%	4.7%	5.5%	2.8%	0.6%	4.5%
200	12.8%	0.1%	1.0%	1.7%	5.7%	1.9%	0.9%	15.9%	1.2%	17.1%	2.5%	1.6%	0.0%	2.7%	4.1%	1.9%	2.1%	24.9%	0.4%	1.6%
201	24.7%	0.0%	8.0%	8.2%	0.6%	15.0%	1.7%	0.7%	2.0%	2.4%	0.4%	3.3%	0.2%	9.7%	5.3%	7.3%	7.2%	2.8%	0.0%	0.4%
202	19.6%	0.0%	3.7%	8.8%	0.7%	8.2%	0.6%	1.1%	5.0%	2.3%	1.1%	4.9%	0.1%	13.8%	6.3%	12.9%	8.6%	2.1%	0.0%	0.2%
203	11.7%	0.1%	0.2%	9.2%	1.6%	1.7%	1.3%	1.6%	1.0%	3.3%	0.8%	0.4%	0.1%	36.4%	18.9%	3.4%	1.5%	1.4%	0.2%	5.5%
204	4.3%	0.0%	10.0%	13.6%	0.1%	2.6%	3.2%	0.8%	3.9%	3.8%	0.2%	6.6%	0.1%	10.7%	28.4%	4.0%	5.4%	1.7%	0.2%	0.2%
205	17.7%	0.1%	1.3%	3.6%	4.3%	2.7%	1.6%	3.0%	1.2%	11.1%	0.9%	2.3%	0.3%	4.8%	6.8%	10.0%	11.7%	11.1%	2.1%	3.4%
206	17.3%	5.4%	1.4%	7.1%	5.7%	1.9%	1.1%	1.0%	0.4%	19.5%	1.7%	1.3%	0.0%	3.6%	11.0%	6.8%	3.4%	1.4%	0.9%	9.0%
207	4.2%	0.1%	0.6%	6.8%	15.7%	0.7%	1.0%	4.8%	1.3%	32.1%	2.0%	0.5%	0.0%	2.9%	4.1%	3.8%	4.4%	3.8%	0.3%	10.9%
208	23.4%	0.0%	8.2%	7.7%	0.4%	6.4%	1.1%	0.7%	4.3%	2.1%	0.2%	4.7%	10.4%	4.1%	6.9%	10.6%	6.4%	2.0%	0.1%	0.3%
209	8.7%	0.0%	16.8%	8.1%	1.2%	3.1%	1.0%	3.3%	1.4%	6.1%	0.5%	4.1%	0.3%	5.9%	5.0%	17.2%	7.9%	7.4%	0.1%	2.1%
210	5.9%	0.1%	0.1%	0.1%	47.3%	0.6%	0.0%	1.3%	0.0%	28.8%	2.1%	0.1%	0.1%	0.1%	0.0%	1.0%	0.6%	1.8%	0.0%	10.0%
211	13.6%	0.6%	3.9%	7.9%	1.1%	3.0%	2.4%	1.5%	6.2%	7.5%	1.0%	7.0%	0.8%	5.5%	20.6%	8.0%	5.7%	2.4%	0.0%	1.3%
212	17.5%	0.0%	6.4%	10.9%	0.9%	2.2%	1.6%	1.2%	4.6%	5.2%	0.8%	3.6%	0.8%	8.9%	7.5%	8.7%	5.4%	2.3%	0.0%	11.7%
213	10.1%	0.3%	1.5%	2.0%	16.3%	2.1%	1.2%	4.7%	0.6%	26.5%	2.6%	3.2%	0.0%	1.3%	1.3%	4.6%	9.7%	6.0%	0.8%	5.3%

214	66.3%	0.4%	0.4%	0.7%	0.9%	8.1%	0.6%	1.3%	0.5%	5.4%	1.3%	0.8%	0.3%	0.5%	0.2%	5.9%	2.1%	4.2%	0.1%	0.2%
215	3.9%	0.1%	11.9%	11.9%	0.0%	4.6%	0.6%	0.7%	1.0%	1.7%	0.0%	4.3%	25.4%	1.0%	4.1%	11.9%	16.1%	0.5%	0.0%	0.3%
216	18.9%	0.0%	7.5%	7.1%	0.1%	8.7%	0.5%	0.5%	5.3%	2.2%	0.5%	2.5%	21.4%	6.0%	3.0%	8.7%	4.6%	2.3%	0.1%	0.2%
217	27.3%	0.0%	13.2%	18.9%	0.3%	5.0%	0.6%	0.4%	4.5%	0.4%	0.1%	1.8%	5.3%	4.9%	2.7%	8.3%	4.7%	1.2%	0.1%	0.3%
218	13.4%	0.2%	10.3%	16.8%	1.7%	2.0%	1.8%	1.8%	0.5%	8.4%	1.6%	2.5%	0.7%	19.8%	1.4%	5.3%	3.0%	5.7%	2.3%	0.7%
219	7.2%	0.1%	0.6%	2.0%	0.5%	1.6%	0.8%	5.9%	7.5%	11.9%	0.9%	1.2%	0.4%	10.2%	37.5%	2.3%	1.3%	7.1%	0.3%	0.7%
220	23.9%	0.0%	10.1%	12.4%	0.1%	6.8%	0.9%	0.5%	4.8%	1.1%	0.4%	2.7%	1.1%	11.0%	7.2%	6.1%	8.6%	1.9%	0.1%	0.2%
221	16.9%	0.2%	3.0%	11.6%	5.7%	1.2%	1.1%	3.6%	2.9%	13.9%	3.1%	0.7%	2.3%	4.5%	14.1%	7.0%	3.3%	2.2%	1.1%	1.3%
222	1.3%	0.1%	0.2%	0.2%	16.1%	0.2%	1.8%	1.6%	0.3%	15.2%	0.9%	0.1%	0.2%	0.5%	0.8%	0.2%	0.2%	4.3%	11.2%	44.6%
223	8.1%	0.0%	20.9%	18.8%	0.3%	2.2%	1.7%	0.3%	4.7%	1.8%	0.2%	5.7%	0.3%	14.5%	9.7%	4.4%	4.2%	1.5%	0.2%	0.7%
224	15.1%	0.0%	9.8%	8.9%	0.1%	2.4%	1.1%	0.2%	7.6%	1.1%	0.1%	5.8%	0.6%	13.7%	15.8%	10.6%	5.4%	0.9%	0.1%	0.6%
225	9.0%	0.2%	0.3%	0.9%	7.3%	1.0%	0.7%	10.7%	1.5%	23.4%	3.6%	0.9%	0.6%	2.1%	1.5%	2.5%	20.7%	8.8%	0.1%	4.2%
226	7.8%	0.7%	1.1%	4.4%	4.0%	1.7%	0.4%	5.1%	2.0%	17.3%	4.0%	1.9%	0.5%	5.9%	2.8%	2.5%	11.1%	25.0%	0.3%	1.4%
227	15.0%	0.0%	6.1%	6.9%	0.5%	10.0%	0.7%	0.9%	6.8%	1.7%	0.3%	6.2%	0.6%	6.4%	5.8%	16.1%	14.5%	1.0%	0.0%	0.5%
228	6.4%	0.2%	5.3%	1.4%	0.2%	44.0%	1.2%	0.2%	1.0%	0.7%	0.2%	5.7%	1.0%	2.7%	1.2%	21.7%	6.3%	0.4%	0.0%	0.2%
229	16.5%	0.2%	0.8%	1.5%	7.7%	2.5%	1.4%	4.1%	0.4%	4.6%	1.8%	1.6%	1.1%	1.3%	1.0%	5.3%	6.6%	22.3%	9.9%	9.7%
230	18.0%	0.0%	10.2%	9.6%	0.1%	4.3%	1.2%	1.0%	8.5%	3.4%	0.3%	4.4%	0.6%	11.4%	11.2%	5.1%	7.0%	3.5%	0.0%	0.1%
231	11.8%	0.0%	6.4%	19.0%	0.4%	2.3%	0.5%	1.0%	6.3%	3.5%	0.3%	3.4%	4.7%	7.6%	14.9%	5.6%	10.4%	1.8%	0.1%	0.2%
232	15.9%	0.0%	0.1%	0.1%	4.1%	0.9%	0.2%	7.0%	2.0%	40.2%	2.5%	0.1%	0.0%	0.5%	4.4%	0.7%	2.9%	17.0%	0.1%	1.3%
233	10.1%	0.2%	9.7%	20.6%	0.3%	2.7%	1.2%	0.7%	6.8%	3.7%	0.8%	5.3%	0.6%	8.3%	12.8%	3.4%	10.0%	2.7%	0.0%	0.3%
234	16.5%	0.6%	6.1%	5.0%	0.9%	3.7%	0.6%	0.9%	8.1%	3.1%	1.0%	2.8%	5.0%	6.5%	19.3%	6.5%	8.1%	4.9%	0.0%	0.4%
235	11.2%	0.1%	0.3%	1.2%	3.4%	0.9%	0.2%	12.3%	1.0%	25.6%	16.4%	0.2%	0.1%	1.9%	1.3%	1.3%	2.8%	9.3%	1.1%	9.6%
236	3.3%	0.0%	4.2%	33.5%	0.2%	1.9%	0.5%	1.2%	3.5%	7.9%	0.3%	1.2%	0.0%	7.7%	27.0%	3.0%	1.7%	2.7%	0.0%	0.2%
237	11.6%	0.0%	21.0%	13.6%	0.2%	3.9%	0.8%	0.3%	7.6%	1.1%	0.3%	8.3%	0.2%	10.9%	9.1%	4.6%	5.5%	0.5%	0.1%	0.4%
238	18.6%	0.0%	3.5%	5.6%	0.6%	2.6%	0.4%	7.9%	6.3%	6.4%	2.0%	0.9%	0.7%	8.8%	14.1%	6.5%	8.3%	5.7%	0.7%	0.5%
239	24.0%	0.0%	0.1%	0.1%	2.9%	1.7%	0.0%	21.5%	0.1%	24.6%	1.5%	0.1%	0.3%	0.1%	0.1%	1.1%	1.2%	19.8%	0.0%	0.8%
240	4.8%	4.5%	1.0%	1.4%	3.7%	0.8%	0.5%	15.1%	1.1%	32.4%	3.6%	1.0%	0.1%	2.4%	3.0%	3.5%	10.1%	9.8%	0.0%	1.1%
241	39.4%	0.0%	3.4%	1.4%	0.2%	12.5%	2.0%	0.7%	1.2%	1.2%	0.4%	2.6%	1.4%	3.4%	2.8%	12.2%	12.7%	2.1%	0.1%	0.3%
242	12.0%	0.1%	20.3%	6.2%	0.3%	9.4%	0.8%	0.8%	5.7%	3.1%	0.7%	5.5%	5.8%	7.3%	4.6%	7.7%	6.5%	2.6%	0.0%	0.5%
243	10.3%	0.0%	6.7%	5.9%	0.2%	31.8%	0.8%	0.6%	3.3%	2.4%	0.7%	3.1%	3.0%	8.3%	6.3%	7.6%	6.8%	1.7%	0.0%	0.5%
244	12.4%	0.0%	3.0%	2.4%	0.7%	5.6%	0.5%	0.3%	1.6%	1.5%	0.1%	1.0%	54.3%	2.4%	3.6%	5.9%	2.5%	1.9%	0.0%	0.2%
245	13.3%	0.2%	7.9%	3.6%	1.2%	31.7%	0.8%	0.4%	2.3%	0.3%	0.2%	4.4%	7.2%	2.9%	2.8%	12.9%	5.5%	1.6%	0.0%	0.7%
246	17.1%	0.1%	5.9%	9.0%	0.2%	22.3%	0.8%	1.0%	4.8%	1.4%	0.2%	2.2%	4.9%	7.7%	5.5%	5.9%	8.5%	2.3%	0.0%	0.1%
247	5.5%	0.1%	1.1%	1.2%	3.9%	1.2%	0.4%	17.4%	0.2%	23.3%	2.6%	0.5%	1.0%	1.1%	4.1%	1.0%	7.5%	26.3%	0.0%	1.4%
248	7.0%	0.0%	27.8%	6.8%	0.0%	4.5%	0.2%	0.2%	5.5%	1.3%	0.1%	6.4%	8.4%	4.9%	2.5%	12.0%	11.6%	0.6%	0.0%	0.1%
249	11.7%	0.3%	3.2%	2.6%	0.3%	9.7%	1.6%	4.9%	2.3%	11.0%	1.3%	1.9%	17.5%	2.6%	4.2%	10.7%	7.1%	6.5%	0.3%	0.0%
250	16.3%	0.0%	9.7%	21.0%	0.0%	11.0%	1.3%	1.3%	1.7%	1.0%	1.0%	3.0%	1.3%	7.7%	2.0%	10.7%	7.0%	1.3%	0.3%	2.3%
251	5.4%	0.3%	1.7%	2.0%	2.3%	3.0%	0.7%	5.4%	5.0%	41.8%	5.7%	0.3%	1.0%	2.0%	4.0%	4.3%	9.0%	4.0%	0.0%	2.0%
252	31.7%	0.1%	1.3%	3.1%	0.3%	6.4%	0.5%	0.7%	0.8%	2.4%	0.2%	0.6%	15.3%	1.7%	3.3%	15.4%	11.7%	4.2%	0.0%	0.2%
253	14.8%	0.0%	10.6%	17.3%	0.1%	5.1%	0.4%	0.4%	9.8%	4.1%	1.3%	2.1%	4.4%	10.6%	6.2%	5.0%	5.7%	2.1%	0.0%	0.1%
254	11.2%	0.0%	18.9%	10.0%	0.2%	2.6%	1.7%	1.3%	6.6%	2.5%	0.3%	5.0%	0.7%	9.3%	7.9%	7.7%	8.7%	3.5%	0.0%	1.7%
255	1.7%	0.1%	0.0%	0.0%	1.6%	0.1%	0.1%	0.5%	0.0%	4.5%	0.0%	0.0%	0.0%	0.0%	0.0%	0.1%	0.2%	3.5%	81.4%	6.2%
256	2.5%	0.0%	11.7%	4.2%	30.5%	1.3%	0.9%	1.1%	0.9%	3.1%	2.1%	4.1%	1.0%	5.7%	7.1%	2.6%	3.5%	3.2%	6.6%	7.8%
257	27.1%	0.1%	18.7%	10.0%	0.2%	4.7%	0.5%	0.8%	5.2%	0.1%	0.1%	3.3%	1.3%	5.2%	3.5%	7.7%	9.9%	1.5%	0.1%	0.1%
258	41.4%	1.1%	5.7%	1.1%	0.2%	0.9%	0.2%	2.6%	1.1%	8.7%	1.5%	2.0%	0.2%	4.9%	1.0%	7.7%	10.9%	8.1%	0.5%	0.1%
259	25.6%	2.3%	2.3%	0.2%	1.1%	1.6%	3.5%	6.7%	0.1%	9.2%	8.2%	2.8%	0.0%	3.3%	0.1%	17.2%	8.2%	5.1%	0.0%	2.6%

260	8.5%	0.0%	7.5%	3.7%	0.1%	7.8%	0.7%	0.3%	1.0%	0.3%	0.1%	2.4%	0.5%	2.2%	2.3%	18.2%	42.9%	1.3%	0.0%	0.3%
261	22.9%	0.1%	5.4%	7.1%	0.2%	5.2%	1.0%	1.1%	5.1%	6.0%	0.4%	3.7%	4.0%	9.3%	7.6%	6.1%	9.8%	3.8%	0.1%	1.2%
262	7.7%	0.2%	0.1%	0.9%	1.0%	0.4%	1.0%	1.4%	19.8%	4.6%	0.8%	1.1%	0.4%	2.2%	25.9%	2.5%	6.6%	20.1%	1.4%	1.8%
263	5.1%	0.0%	0.7%	0.2%	5.8%	2.0%	0.8%	38.3%	0.1%	16.7%	2.7%	2.0%	0.0%	4.2%	1.8%	3.2%	2.6%	9.8%	0.5%	3.3%
264	6.0%	0.0%	41.3%	12.5%	0.0%	5.7%	0.5%	0.1%	3.8%	0.6%	0.1%	11.6%	1.4%	5.0%	2.7%	3.5%	4.3%	0.8%	0.0%	0.0%
265	20.0%	0.0%	7.1%	9.5%	0.4%	9.1%	0.8%	1.4%	2.9%	17.3%	1.4%	1.1%	0.3%	10.8%	7.7%	4.8%	2.8%	2.1%	0.1%	0.3%
266	0.9%	0.1%	0.0%	0.5%	2.5%	0.1%	0.1%	6.4%	0.0%	44.5%	27.9%	0.2%	0.0%	1.0%	0.0%	0.2%	1.3%	4.8%	0.5%	9.2%
267	6.6%	0.0%	3.6%	2.6%	0.6%	5.3%	3.5%	0.9%	18.1%	2.4%	1.2%	4.3%	0.0%	5.0%	34.4%	4.4%	3.4%	1.2%	0.9%	1.5%
268	10.6%	0.0%	8.6%	11.2%	0.1%	11.0%	0.6%	1.2%	10.5%	1.7%	0.4%	4.0%	0.4%	12.4%	5.8%	6.7%	11.9%	1.6%	1.2%	0.1%
269	8.9%	0.1%	0.8%	1.3%	3.1%	0.4%	0.1%	22.2%	0.1%	20.9%	3.4%	0.1%	0.0%	2.5%	0.5%	0.8%	2.1%	32.1%	0.0%	0.5%
270	4.4%	0.7%	3.6%	55.5%	0.7%	3.7%	0.3%	1.2%	0.4%	2.2%	0.6%	3.1%	0.0%	9.9%	2.4%	2.7%	3.6%	3.7%	0.7%	0.6%
271	8.5%	0.3%	20.5%	6.9%	0.2%	2.6%	2.0%	3.0%	6.6%	5.6%	0.6%	5.0%	0.1%	6.0%	6.7%	11.9%	9.6%	4.0%	0.1%	0.1%
272	15.2%	0.1%	10.5%	13.1%	0.7%	4.6%	2.1%	0.8%	5.9%	2.8%	0.5%	2.6%	0.1%	10.4%	10.5%	7.9%	9.2%	1.7%	0.1%	1.3%
273	14.1%	0.1%	0.0%	0.6%	1.4%	1.5%	0.4%	6.6%	0.3%	52.1%	3.7%	0.3%	0.1%	4.7%	2.0%	1.7%	2.6%	7.5%	0.1%	0.4%
274	25.3%	0.1%	5.1%	3.4%	0.9%	13.5%	0.7%	3.6%	0.4%	13.5%	0.4%	2.4%	0.0%	2.9%	3.6%	9.1%	6.3%	5.7%	0.2%	2.8%
275	12.9%	0.0%	26.0%	12.1%	0.1%	6.3%	1.6%	0.4%	3.3%	1.5%	0.3%	4.9%	0.2%	5.6%	8.3%	7.0%	7.4%	1.7%	0.1%	0.2%
276	11.1%	0.3%	17.2%	12.3%	0.2%	6.1%	3.2%	1.2%	7.1%	1.6%	0.7%	4.0%	0.0%	9.6%	12.8%	5.8%	4.8%	1.4%	0.0%	0.4%
277	14.6%	0.0%	0.1%	0.2%	2.7%	0.4%	0.3%	13.6%	0.1%	30.9%	9.3%	0.3%	0.0%	0.7%	2.2%	1.5%	2.0%	16.5%	0.0%	4.7%
278	12.2%	0.0%	4.1%	10.3%	0.3%	2.2%	2.0%	4.6%	3.5%	9.9%	2.1%	2.5%	0.0%	11.5%	8.1%	3.6%	5.1%	17.8%	0.0%	0.2%

331	1.6%	0.0%	6.3%	3.2%	0.0%	0.0%	30.2%	0.0%	0.0%	0.0%	0.0%	1.6%	0.0%	50.8%	6.3%	0.0%	0.0%	0.0%	0.0%	0.0%
332	8.4%	0.0%	18.1%	9.9%	0.1%	4.0%	4.4%	0.0%	6.8%	0.5%	0.1%	5.1%	0.8%	26.8%	5.5%	4.0%	4.6%	0.6%	0.1%	0.0%
333	12.0%	0.0%	0.1%	0.2%	2.7%	0.0%	0.2%	2.7%	0.7%	61.1%	5.5%	0.1%	0.2%	0.2%	2.1%	0.6%	6.3%	5.2%	0.1%	0.2%
334	5.0%	0.0%	2.8%	13.2%	0.0%	1.9%	2.3%	0.3%	12.5%	1.5%	0.3%	1.9%	1.3%	16.6%	25.3%	5.9%	6.7%	2.2%	0.1%	0.2%
335	13.6%	0.1%	8.2%	19.3%	0.2%	3.4%	3.1%	0.6%	3.5%	2.7%	0.3%	3.2%	0.2%	9.6%	13.0%	8.8%	8.3%	1.3%	0.0%	0.5%
336	43.5%	0.0%	1.3%	5.7%	0.4%	2.2%	0.3%	1.5%	7.0%	1.8%	0.5%	1.2%	0.2%	5.6%	17.9%	4.4%	5.0%	1.9%	0.0%	0.0%
337	5.3%	0.0%	0.5%	1.3%	5.5%	0.4%	0.2%	5.0%	0.3%	62.1%	8.0%	0.2%	1.0%	0.6%	1.1%	0.8%	1.1%	6.2%	0.2%	0.3%
338	19.4%	0.0%	7.9%	35.6%	0.2%	1.5%	1.7%	0.4%	2.8%	3.2%	0.2%	3.8%	0.3%	4.2%	6.2%	5.4%	4.6%	2.1%	0.0%	0.3%
339	6.4%	0.0%	8.7%	16.6%	0.1%	4.9%	2.0%	0.5%	0.6%	0.7%	0.3%	3.5%	0.1%	1.4%	1.5%	28.3%	22.9%	0.7%	0.0%	0.7%
340	0.9%	0.1%	0.5%	0.8%	0.1%	0.8%	3.6%	1.4%	0.9%	0.9%	0.6%	1.7%	0.1%	2.5%	82.4%	1.3%	0.5%	0.6%	0.1%	0.2%
341	16.5%	0.0%	8.8%	1.4%	0.0%	1.9%	1.5%	2.3%	38.6%	0.4%	0.1%	0.6%	3.9%	2.4%	9.1%	2.7%	4.5%	5.0%	0.0%	0.0%
342	6.0%	0.2%	1.8%	4.7%	0.3%	0.6%	0.8%	8.5%	1.1%	25.6%	1.0%	0.2%	0.8%	2.0%	5.1%	2.0%	8.7%	28.7%	1.2%	0.7%
343	0.1%	0.0%	0.0%	0.0%	0.1%	0.0%	0.0%	61.2%	0.0%	3.9%	0.2%	0.0%	0.0%	0.2%	0.0%	0.0%	1.2%	32.9%	0.0%	0.0%
344	2.3%	0.2%	35.1%	36.8%	0.4%	12.9%	2.7%	0.3%	0.2%	0.3%	0.1%	2.8%	0.1%	0.9%	0.4%	2.5%	0.4%	0.8%	0.0%	0.9%
345	1.1%	0.0%	0.4%	2.4%	0.0%	0.0%	0.6%	1.2%	8.5%	5.2%	2.4%	0.4%	0.0%	38.2%	35.8%	0.2%	1.9%	1.3%	0.0%	0.0%
346	98.7%	0.1%	0.0%	0.0%	0.0%	0.2%	0.0%	0.0%	0.0%	0.0%	0.0%	0.0%	0.0%	0.0%	0.0%	0.1%	0.2%	0.7%	0.0%	0.0%
347	0.8%	1.4%	0.0%	0.6%	0.0%	0.1%	0.0%	6.4%	69.5%	1.4%	1.7%	0.0%	0.0%	2.3%	2.6%	0.5%	5.1%	7.5%	0.0%	0.0%
348	4.1%	1.1%	0.3%	1.2%	0.2%	79.3%	0.2%	0.4%	0.4%	1.7%	0.2%	0.3%	0.0%	0.3%	2.3%	5.7%	0.6%	0.5%	0.8%	0.1%
349	4.8%	0.3%	0.0%	0.3%	1.1%	0.0%	0.1%	34.7%	1.1%	23.5%	12.5%	0.0%	0.0%	0.4%	1.0%	0.2%	0.4%	18.8%	0.2%	0.7%
350	0.1%	0.0%	0.0%	0.0%	0.3%	0.0%	0.0%	11.8%	0.0%	76.9%	0.9%	0.0%	0.0%	0.1%	0.0%	0.0%	0.2%	9.6%	0.0%	0.1%
351	7.9%	0.6%	0.0%	0.2%	0.0%	0.1%	0.0%	6.3%	0.1%	1.0%	64.7%	1.5%	0.0%	6.9%	1.3%	1.9%	1.6%	5.7%	0.0%	0.0%
352	20.2%	0.2%	4.4%	23.5%	0.4%	5.0%	2.2%	0.3%	10.0%	3.5%	0.5%	1.1%	0.0%	6.8%	4.0%	4.3%	8.8%	4.2%	0.0%	0.7%
353	7.6%	0.2%	1.1%	4.7%	0.2%	0.6%	3.8%	2.0%	9.0%	6.2%	2.8%	1.8%	0.0%	14.4%	28.9%	4.6%	5.4%	5.2%	0.3%	1.0%
354	1.9%	0.2%	1.0%	2.6%	5.7%	3.2%	11.7%	0.2%	5.4%	13.4%	4.8%	5.9%	0.0%	8.1%	17.1%	1.7%	2.0%	0.3%	0.7%	14.0%
355	2.7%	0.1%	6.0%	1.3%	0.0%	46.4%	3.9%	0.0%	6.0%	0.2%	0.1%	8.1%	0.6%	4.6%	14.3%	4.8%	0.6%	0.1%	0.0%	0.1%
356	0.6%	14.7%	0.0%	0.1%	1.9%	0.7%	0.1%	18.4%	0.4%	34.2%	10.1%	0.0%	0.0%	0.1%	0.4%	0.4%	1.9%	14.5%	0.6%	0.8%

357	1.8%	0.1%	19.2%	1.3%	0.0%	7.0%	0.3%	0.0%	0.6%	0.2%	0.0%	3.7%	3.5%	0.3%	1.1%	34.7%	26.1%	0.0%	0.0%	0.0%
358	17.0%	0.0%	3.5%	51.1%	0.0%	1.0%	0.4%	0.9%	0.1%	1.4%	2.6%	0.2%	10.6%	2.4%	1.6%	2.8%	0.9%	2.9%	0.0%	0.4%
359	9.7%	0.0%	27.7%	22.7%	0.1%	2.6%	1.3%	0.4%	0.9%	0.5%	0.3%	2.1%	16.0%	6.1%	2.0%	2.8%	1.9%	2.7%	0.0%	0.1%
360	15.2%	0.0%	11.0%	42.0%	0.0%	1.7%	0.8%	0.2%	1.1%	0.3%	0.1%	0.2%	0.2%	16.8%	4.1%	1.6%	3.4%	1.2%	0.0%	0.0%
361	96.4%	0.0%	0.0%	0.0%	0.0%	1.9%	0.0%	0.0%	0.0%	0.0%	0.0%	0.0%	0.0%	0.0%	0.0%	1.4%	0.1%	0.2%	0.0%	0.0%
362	2.1%	0.0%	0.1%	0.2%	53.6%	0.3%	6.7%	0.3%	0.0%	5.3%	1.5%	0.0%	0.0%	0.2%	0.8%	0.2%	0.2%	0.6%	4.5%	23.2%
363	16.1%	0.0%	11.8%	11.7%	0.0%	3.1%	2.1%	0.4%	8.2%	1.7%	0.1%	2.1%	0.0%	6.3%	26.1%	2.9%	5.2%	2.0%	0.0%	0.1%
364	5.2%	0.1%	0.2%	3.0%	2.1%	0.1%	1.3%	6.4%	2.9%	24.3%	4.9%	0.2%	0.0%	3.8%	10.9%	1.2%	2.9%	11.1%	14.4%	4.8%
365	0.0%	0.0%	0.0%	0.0%	0.3%	0.0%	0.0%	23.0%	0.0%	69.3%	7.0%	0.0%	0.0%	0.0%	0.0%	0.0%	0.0%	0.3%	0.0%	0.0%
366	3.9%	0.1%	0.3%	2.4%	0.1%	0.2%	0.5%	1.0%	3.7%	1.7%	0.4%	0.1%	0.0%	23.1%	50.7%	1.6%	2.9%	7.2%	0.0%	0.0%
367	8.2%	0.0%	3.4%	6.6%	0.1%	3.3%	1.4%	0.2%	29.9%	0.6%	0.3%	1.4%	0.0%	6.1%	23.5%	6.2%	4.6%	0.9%	3.3%	0.0%
368	16.8%	1.2%	0.6%	2.4%	0.6%	0.2%	5.7%	3.1%	1.6%	12.6%	3.7%	1.0%	0.0%	7.9%	7.7%	3.0%	19.3%	5.8%	0.3%	6.5%
369	47.0%	0.2%	0.0%	0.0%	0.0%	0.2%	0.0%	0.0%	0.0%	0.0%	0.0%	0.0%	0.0%	0.0%	0.0%	51.8%	0.4%	0.4%	0.0%	0.0%
370	1.0%	0.0%	0.4%	1.7%	2.5%	0.4%	0.8%	0.0%	0.6%	0.3%	47.1%	1.5%	0.0%	26.9%	10.5%	3.5%	0.9%	0.2%	0.1%	1.6%
371	8.0%	0.2%	32.8%	8.0%	0.0%	1.3%	7.0%	0.1%	2.0%	0.8%	0.3%	12.7%	0.0%	5.5%	9.8%	8.1%	2.6%	0.6%	0.0%	0.1%
372	5.2%	0.5%	0.8%	6.1%	0.8%	1.5%	7.5%	0.1%	6.7%	4.6%	1.1%	3.4%	0.0%	10.7%	23.9%	10.6%	14.8%	0.3%	0.7%	0.5%
373	0.5%	0.9%	1.6%	0.4%	0.0%	18.3%	2.1%	0.0%	2.4%	0.0%	0.0%	38.6%	0.1%	4.0%	28.6%	2.4%	0.1%	0.0%	0.0%	0.0%
374	1.3%	1.2%	0.1%	1.3%	0.0%	0.2%	0.3%	10.8%	8.7%	13.5%	6.1%	0.3%	0.0%	5.0%	19.3%	1.3%	14.0%	16.5%	0.0%	0.0%
375	1.2%	0.0%	0.9%	0.4%	0.0%	1.5%	0.5%	0.0%	38.1%	0.5%	0.1%	1.4%	9.9%	0.4%	13.3%	15.6%	15.9%	0.2%	0.0%	0.0%
376	1.1%	0.0%	0.0%	0.1%	0.3%	0.0%	0.0%	10.4%	1.4%	48.3%	22.9%	0.0%	1.4%	0.5%	1.3%	0.1%	0.5%	11.5%	0.0%	0.0%
377	14.4%	0.1%	1.4%	3.7%	0.8%	4.6%	5.1%	3.5%	7.2%	4.3%	0.4%	0.8%	1.0%	4.1%	28.4%	5.4%	3.9%	10.3%	0.1%	0.7%
378	8.1%	0.0%	30.6%	32.9%	0.0%	0.5%	1.1%	0.6%	1.8%	1.4%	0.2%	1.0%	0.2%	8.5%	3.6%	2.0%	3.7%	3.6%	0.0%	0.1%
379	2.6%	0.0%	0.0%	0.5%	1.0%	0.0%	0.1%	17.8%	0.0%	17.9%	1.6%	0.0%	0.0%	0.2%	0.1%	0.3%	3.0%	54.5%	0.0%	0.3%
380	93.9%	2.4%	0.0%	0.0%	0.0%	0.1%	0.0%	0.0%	0.0%	0.0%	0.0%	0.0%	0.0%	0.0%	0.0%	3.0%	0.1%	0.5%	0.0%	0.0%
381	13.3%	0.1%	5.9%	28.0%	0.1%	2.1%	1.9%	0.3%	4.2%	1.4%	0.6%	0.8%	0.0%	15.0%	17.4%	2.6%	3.8%	2.2%	0.3%	0.2%
382	26.9%	0.1%	9.4%	9.4%	0.2%	3.4%	1.6%	1.0%	1.9%	2.8%	3.1%	2.4%	0.0%	9.8%	11.9%	7.8%	4.5%	2.7%	0.7%	0.2%
383	0.2%	0.0%	0.0%	0.0%	2.3%	0.0%	0.0%	31.8%	0.0%	26.6%	1.8%	0.0%	0.0%	0.0%	0.0%	0.0%	0.4%	36.8%	0.0%	0.0%
384	0.0%	0.0%	0.0%	0.0%	0.1%	0.0%	0.0%	35.4%	0.0%	22.9%	1.2%	0.0%	0.0%	0.0%	0.0%	0.0%	0.1%	40.2%	0.0%	0.0%

Table S2: Relative representation of amino acids in alignments of NasR homologs.
For each position corresponding to a residue in *K. oxytoca* NasR in the multiple sequence alignment of NIT domains (positions 41-278) or ANTAR domains (positions 331-384) for proteins containing both NIT and ANTAR domains, the percentage of sequences with each amino acid at that position is shown. These data were compared with that in Table S1 to generate the data for Figure 4.

Position in NasR	A	C	D	E	F	G	H	I	K	L	M	N	P	Q	R	S	T	V	W	Y
42	0.0%	1.3%	0.0%	0.0%	10.9%	0.0%	0.0%	0.4%	0.0%	80.8%	3.1%	0.0%	0.0%	0.0%	0.0%	0.0%	0.0%	3.1%	0.4%	0.0%
43	0.9%	0.0%	0.0%	0.0%	0.0%	0.0%	0.0%	40.3%	0.0%	0.4%	0.9%	0.0%	0.0%	0.0%	0.0%	0.0%	0.9%	56.7%	0.0%	0.0%
44	0.0%	0.0%	0.9%	0.4%	0.0%	0.0%	96.5%	0.0%	0.0%	0.0%	0.0%	0.0%	0.0%	2.2%	0.0%	0.0%	0.0%	0.0%	0.0%	0.0%
45	39.0%	1.7%	0.4%	4.8%	0.4%	8.2%	0.9%	0.0%	0.0%	3.5%	11.7%	0.4%	0.0%	12.6%	3.5%	3.5%	0.4%	8.7%	0.0%	0.4%
46	0.0%	0.0%	0.0%	0.0%	0.4%	0.0%	0.0%	1.3%	0.0%	97.4%	0.9%	0.0%	0.0%	0.0%	0.0%	0.0%	0.0%	0.0%	0.0%	0.0%
47	0.0%	0.0%	0.0%	0.0%	0.0%	0.0%	0.0%	0.0%	0.0%	0.0%	0.0%	0.0%	0.0%	100.0%	0.0%	0.0%	0.0%	0.0%	0.0%	0.0%
48	3.5%	6.5%	0.0%	0.0%	0.0%	0.4%	1.3%	0.0%	22.9%	0.9%	0.0%	0.0%	0.0%	1.7%	61.5%	0.4%	0.4%	0.0%	0.0%	0.4%
49	0.0%	0.0%	0.0%	100.0%	0.0%	0.0%	0.0%	0.0%	0.0%	0.0%	0.0%	0.0%	0.0%	0.0%	0.0%	0.0%	0.0%	0.0%	0.0%	0.0%
50	0.0%	0.0%	0.0%	0.0%	0.0%	0.0%	0.0%	0.0%	0.0%	0.0%	0.0%	0.0%	0.0%	0.0%	100.0%	0.0%	0.0%	0.0%	0.0%	0.0%
51	0.0%	0.0%	0.0%	0.0%	0.0%	100.0%	0.0%	0.0%	0.0%	0.0%	0.0%	0.0%	0.0%	0.0%	0.0%	0.0%	0.0%	0.0%	0.0%	0.0%
52	43.7%	0.4%	0.0%	0.0%	0.9%	0.0%	0.0%	6.5%	0.0%	19.9%	6.5%	0.0%	0.0%	0.4%	0.4%	2.6%	7.8%	2.6%	0.9%	7.4%
53	6.1%	0.4%	0.0%	0.0%	0.0%	0.4%	0.0%	0.4%	0.0%	0.0%	0.0%	0.4%	0.0%	0.0%	0.0%	82.7%	7.4%	2.2%	0.0%	0.0%
54	0.4%	2.2%	0.0%	0.0%	0.0%	1.3%	0.0%	0.9%	0.0%	0.0%	0.4%	78.8%	0.0%	0.0%	0.0%	10.4%	5.6%	0.0%	0.0%	0.0%
55	0.9%	0.0%	0.0%	0.0%	0.4%	0.9%	0.0%	36.8%	0.0%	29.9%	2.6%	0.0%	0.0%	0.0%	0.9%	0.4%	0.0%	27.3%	0.0%	0.0%
56	0.0%	0.4%	0.0%	0.0%	33.9%	0.0%	2.6%	0.0%	0.0%	5.2%	0.0%	0.0%	0.0%	0.0%	0.0%	0.4%	1.3%	1.7%	12.6%	41.7%
57	0.0%	0.0%	0.0%	0.0%	0.0%	0.0%	0.0%	5.2%	0.0%	93.0%	0.0%	0.0%	0.0%	0.0%	0.0%	0.0%	0.0%	1.7%	0.0%	0.0%
58	36.1%	25.2%	0.0%	0.0%	0.0%	27.0%	0.0%	0.4%	0.4%	0.0%	0.0%	0.0%	0.0%	0.0%	0.4%	4.3%	1.3%	4.8%	0.0%	0.0%
59	1.7%	0.0%	1.3%	0.0%	0.0%	3.5%	1.3%	0.0%	0.0%	0.9%	0.0%	2.2%	0.0%	0.4%	1.7%	85.7%	1.3%	0.0%	0.0%	0.0%
60	8.2%	0.0%	6.8%	4.8%	0.5%	25.6%	5.3%	0.0%	6.3%	0.0%	0.0%	3.9%	1.0%	19.8%	12.1%	3.9%	0.0%	1.9%	0.0%	0.0%
61	4.8%	3.0%	1.7%	0.4%	0.0%	82.7%	0.9%	0.0%	0.4%	0.0%	0.0%	0.9%	0.0%	1.3%	1.3%	1.3%	1.3%	0.0%	0.0%	0.0%
62	11.7%	0.9%	5.7%	12.2%	0.0%	3.0%	2.2%	0.0%	7.0%	2.2%	1.3%	0.4%	2.2%	22.2%	12.6%	4.3%	9.6%	2.6%	0.0%	0.0%
63	1.7%	0.0%	1.3%	1.7%	0.4%	2.2%	6.5%	0.0%	2.6%	17.4%	1.3%	0.0%	0.4%	4.3%	53.9%	1.3%	3.9%	0.4%	0.0%	0.4%
64	3.1%	1.3%	0.4%	0.0%	64.9%	1.8%	1.8%	0.0%	0.0%	5.3%	0.0%	0.0%	0.0%	1.8%	0.4%	0.0%	1.3%	0.4%	4.0%	13.3%
65	38.6%	1.0%	2.4%	2.9%	0.5%	17.4%	1.0%	0.0%	4.3%	6.3%	0.0%	0.0%	0.0%	3.9%	7.2%	3.9%	4.8%	5.8%	0.0%	0.0%
66	17.3%	0.0%	19.2%	15.9%	0.0%	3.8%	2.4%	0.0%	1.4%	1.0%	0.0%	1.0%	8.7%	11.1%	5.8%	4.8%	7.2%	0.5%	0.0%	0.0%
67	6.1%	0.4%	2.2%	33.8%	0.0%	0.0%	1.3%	0.9%	1.3%	1.7%	1.7%	0.0%	2.2%	27.7%	12.6%	0.9%	3.5%	3.9%	0.0%	0.0%
68	0.0%	3.0%	0.4%	0.0%	0.0%	0.0%	0.4%	1.3%	0.0%	33.8%	0.4%	0.0%	0.0%	0.0%	57.1%	0.0%	0.0%	1.7%	1.7%	0.0%
69	20.0%	0.0%	11.7%	6.5%	0.0%	4.8%	1.7%	0.4%	2.6%	10.4%	1.3%	0.4%	2.6%	12.2%	13.0%	5.7%	5.7%	0.4%	0.0%	0.4%
70	31.7%	0.0%	3.9%	14.3%	0.4%	2.6%	2.2%	0.0%	0.4%	4.8%	0.4%	0.9%	3.5%	15.7%	4.8%	3.5%	8.3%	2.6%	0.0%	0.0%
71	5.6%	3.0%	0.0%	0.9%	1.3%	2.6%	4.8%	6.1%	0.0%	9.5%	4.8%	0.0%	0.0%	30.3%	18.2%	3.9%	0.9%	0.9%	0.9%	6.5%
72	12.6%	0.0%	0.0%	6.1%	0.0%	3.0%	0.4%	24.2%	0.0%	8.7%	1.7%	0.4%	0.0%	2.6%	8.7%	6.1%	4.3%	20.8%	0.4%	0.0%
73	31.6%	0.0%	6.1%	10.8%	0.0%	3.0%	0.9%	0.9%	3.9%	5.2%	0.0%	0.9%	1.3%	18.2%	8.2%	5.2%	2.6%	1.3%	0.0%	0.0%
74	12.1%	0.4%	19.5%	27.7%	0.0%	1.7%	5.6%	0.9%	1.7%	7.8%	0.4%	2.2%	0.0%	12.6%	1.7%	2.6%	1.7%	1.3%	0.0%	0.0%
75	15.6%	15.6%	0.0%	0.0%	0.4%	2.6%	0.0%	0.4%	0.0%	0.0%	0.0%	3.9%	0.0%	0.4%	0.0%	31.6%	8.7%	20.8%	0.0%	0.0%
76	5.2%	1.3%	21.2%	12.1%	0.0%	1.7%	1.3%	2.2%	2.6%	9.5%	0.4%	3.9%	0.0%	19.5%	6.5%	5.2%	3.0%	4.3%	0.0%	0.0%
77	22.9%	0.0%	4.3%	13.0%	0.4%	1.3%	3.9%	0.9%	0.9%	6.1%	1.3%	0.4%	4.3%	17.3%	15.6%	3.5%	2.6%	1.3%	0.0%	0.0%
78	33.8%	1.7%	0.4%	3.0%	0.0%	2.2%	1.7%	5.6%	0.4%	18.6%	2.2%	0.9%	0.4%	7.4%	3.9%	6.1%	3.5%	8.2%	0.0%	0.0%
79	7.9%	0.9%	0.4%	56.8%	0.0%	0.0%	1.3%	2.2%	0.0%	9.2%	1.3%	0.0%	0.0%	11.8%	3.5%	0.9%	2.2%	1.3%	0.0%	0.4%

80	30.6%	0.0%	5.7%	6.6%	0.0%	1.3%	1.3%	1.3%	2.6%	4.4%	0.0%	0.9%	0.0%	20.1%	10.5%	4.8%	7.4%	2.6%	0.0%	0.0%
81	19.7%	0.0%	6.1%	15.4%	0.9%	1.8%	2.2%	3.9%	1.3%	7.9%	0.4%	1.3%	1.8%	11.4%	12.7%	2.6%	6.1%	4.4%	0.0%	0.0%
82	3.1%	0.0%	0.0%	0.0%	25.0%	0.0%	0.0%	0.4%	0.0%	33.3%	8.3%	0.0%	0.0%	0.0%	0.0%	0.0%	0.0%	28.1%	1.8%	0.0%
83	2.6%	1.3%	3.5%	3.5%	0.4%	0.4%	5.2%	0.9%	1.7%	5.7%	3.9%	0.0%	0.0%	4.4%	60.7%	1.3%	0.0%	0.4%	0.0%	3.9%
84	24.0%	0.0%	7.4%	10.9%	0.0%	3.5%	1.7%	0.9%	2.2%	2.2%	0.0%	2.2%	0.9%	20.5%	10.0%	7.4%	5.2%	0.0%	0.0%	0.9%
85	18.9%	5.8%	1.9%	2.9%	5.8%	6.3%	5.8%	0.0%	0.5%	8.7%	1.5%	0.0%	0.5%	9.2%	17.0%	4.4%	1.5%	3.4%	3.9%	1.9%
86	0.0%	0.4%	0.0%	0.0%	23.9%	0.0%	0.4%	0.9%	0.0%	71.3%	0.4%	0.0%	0.0%	0.4%	0.0%	0.0%	0.0%	1.3%	0.9%	0.0%
87	18.7%	0.0%	27.8%	20.0%	0.4%	2.6%	0.9%	0.0%	5.7%	1.3%	1.3%	0.9%	5.2%	7.0%	0.4%	3.0%	3.9%	0.9%	0.0%	0.0%
88	17.8%	0.0%	3.0%	12.2%	0.4%	3.5%	3.0%	0.9%	0.4%	2.6%	0.4%	1.7%	5.7%	12.2%	14.8%	11.7%	6.1%	1.7%	1.3%	0.4%
89	6.1%	0.0%	0.4%	1.3%	1.3%	0.0%	3.9%	10.4%	0.4%	50.2%	5.2%	0.9%	2.6%	6.1%	0.9%	0.0%	1.7%	6.5%	1.3%	0.9%
90	7.1%	0.0%	29.9%	17.4%	0.4%	3.1%	5.4%	0.0%	1.8%	2.7%	0.4%	4.0%	1.8%	7.1%	3.6%	4.9%	2.2%	1.8%	0.0%	6.3%
91	16.4%	0.0%	1.8%	6.8%	0.0%	2.7%	3.2%	1.4%	1.4%	16.4%	1.8%	0.5%	15.9%	8.2%	2.3%	4.5%	13.2%	3.2%	0.0%	0.5%
92	12.6%	0.0%	15.0%	22.9%	0.9%	6.1%	2.3%	0.9%	1.9%	2.8%	3.3%	5.6%	3.7%	6.1%	3.3%	7.5%	3.7%	1.4%	0.0%	0.0%
93	16.9%	0.0%	7.4%	0.4%	0.9%	19.5%	7.4%	2.2%	0.0%	2.2%	3.5%	13.0%	0.9%	9.1%	0.9%	6.5%	2.6%	6.1%	0.0%	0.9%
94	16.9%	0.4%	1.3%	0.4%	0.9%	24.7%	2.6%	0.0%	4.8%	10.0%	0.0%	7.4%	0.9%	0.0%	7.8%	17.7%	1.7%	0.4%	0.0%	2.2%
95	40.7%	0.4%	0.0%	0.0%	0.4%	1.7%	0.4%	1.3%	0.0%	0.0%	4.3%	0.4%	3.9%	0.0%	0.0%	34.6%	6.9%	4.8%	0.0%	0.0%
96	4.8%	0.0%	0.0%	0.0%	0.0%	2.2%	1.7%	0.0%	0.0%	9.1%	0.4%	0.4%	1.3%	2.6%	74.3%	2.6%	0.4%	0.0%	0.0%	0.0%
97	0.4%	0.4%	0.0%	0.0%	6.5%	0.0%	0.0%	3.5%	0.0%	77.8%	6.5%	0.0%	0.0%	0.9%	0.0%	0.0%	0.0%	3.0%	0.9%	0.0%
98	1.4%	10.6%	0.0%	0.0%	43.0%	0.0%	0.0%	0.0%	0.0%	33.3%	1.0%	0.0%	0.0%	0.0%	0.0%	0.0%	0.0%	0.5%	0.0%	10.1%
99	8.7%	0.0%	0.4%	0.9%	0.0%	5.2%	3.9%	0.9%	0.9%	1.3%	0.9%	22.2%	0.4%	0.9%	5.7%	36.1%	8.3%	1.3%	2.2%	0.0%
100	2.6%	3.9%	1.3%	0.0%	0.0%	1.7%	4.3%	0.0%	0.4%	3.5%	0.0%	1.3%	0.0%	3.0%	58.3%	9.6%	0.4%	0.0%	9.6%	0.0%
101	5.6%	0.0%	0.0%	0.0%	0.0%	0.0%	0.0%	70.1%	0.0%	8.2%	0.4%	0.0%	0.0%	0.0%	0.0%	0.4%	0.9%	14.3%	0.0%	0.0%
102	81.8%	0.4%	0.0%	0.0%	0.0%	1.3%	0.0%	0.0%	0.0%	9.5%	0.4%	0.0%	0.0%	0.4%	0.0%	0.9%	5.2%	0.0%	0.0%	0.0%
103	10.0%	4.3%	0.0%	0.0%	6.9%	3.9%	6.9%	2.2%	0.0%	15.6%	1.3%	2.2%	0.0%	0.4%	3.9%	10.0%	3.0%	3.0%	7.4%	19.0%
104	52.4%	0.5%	0.0%	0.0%	1.0%	1.5%	0.0%	0.0%	0.0%	0.5%	0.5%	0.0%	0.0%	0.0%	0.0%	1.5%	0.5%	41.7%	0.0%	0.0%
105	0.0%	0.0%	0.0%	0.0%	1.0%	0.0%	0.5%	1.0%	0.0%	75.2%	1.5%	0.0%	0.0%	0.0%	0.0%	0.5%	0.0%	12.1%	8.3%	0.0%
106	1.0%	0.5%	1.5%	0.5%	1.5%	1.5%	39.8%	0.0%	0.0%	2.9%	0.5%	1.5%	6.8%	24.3%	0.0%	0.0%	1.0%	0.0%	4.9%	12.1%
107	26.0%	3.9%	4.3%	1.7%	0.4%	40.7%	0.9%	0.4%	0.0%	0.4%	0.0%	0.9%	0.0%	1.7%	5.6%	9.1%	0.9%	0.4%	0.0%	2.6%
108	0.0%	0.0%	0.0%	0.0%	3.9%	0.0%	0.0%	2.2%	0.0%	88.3%	5.2%	0.0%	0.0%	0.0%	0.0%	0.0%	0.4%	0.0%	0.0%	0.0%
109	4.3%	0.0%	60.9%	18.7%	0.0%	2.6%	0.0%	0.0%	0.4%	0.0%	0.0%	1.7%	0.0%	1.3%	0.0%	7.8%	2.2%	0.0%	0.0%	0.0%
110	25.5%	0.0%	5.2%	15.6%	0.0%	16.0%	2.6%	0.9%	0.0%	3.5%	0.4%	2.2%	0.4%	10.4%	2.2%	7.8%	5.2%	0.9%	0.0%	1.3%
111	0.0%	0.0%	0.0%	0.0%	0.0%	0.0%	0.0%	1.3%	0.0%	95.2%	1.7%	0.0%	0.0%	0.4%	0.0%	0.0%	0.0%	1.3%	0.0%	0.0%
112	6.5%	0.0%	6.1%	3.5%	0.0%	5.2%	0.4%	0.0%	2.2%	0.4%	0.0%	0.4%	64.9%	1.3%	1.7%	4.3%	2.2%	0.9%	0.0%	0.0%
113	37.7%	0.0%	4.8%	14.3%	0.0%	10.8%	0.0%	2.2%	0.0%	4.8%	0.0%	1.3%	1.7%	3.0%	3.0%	5.6%	6.1%	4.3%	0.4%	0.0%
114	0.0%	0.0%	0.0%	0.0%	1.3%	0.0%	0.0%	3.0%	0.0%	89.6%	1.7%	0.0%	0.0%	1.7%	0.0%	0.0%	0.9%	1.7%	0.0%	0.0%
115	0.0%	0.0%	0.0%	0.0%	0.0%	0.0%	0.0%	0.0%	0.0%	0.0%	0.0%	0.0%	0.0%	0.0%	100.0%	0.0%	0.0%	0.0%	0.0%	0.0%
116	15.2%	0.9%	3.9%	15.6%	0.0%	2.2%	6.5%	0.4%	1.7%	2.2%	0.0%	1.7%	1.3%	17.3%	20.8%	5.2%	2.2%	1.7%	0.9%	0.4%
117	10.8%	2.2%	3.9%	4.8%	0.0%	1.3%	1.3%	0.0%	8.7%	0.0%	0.0%	0.4%	0.0%	29.9%	32.5%	3.5%	0.4%	0.4%	0.0%	0.0%
118	1.3%	0.0%	0.0%	0.0%	0.0%	0.0%	0.0%	51.1%	0.0%	3.0%	1.3%	0.4%	0.0%	0.0%	0.9%	0.0%	0.9%	41.1%	0.0%	0.0%
119	16.5%	0.0%	13.9%	10.4%	0.0%	6.9%	2.2%	1.7%	1.7%	3.5%	0.0%	1.7%	0.0%	5.6%	19.9%	10.0%	4.8%	1.3%	0.0%	0.0%
120	26.4%	1.3%	5.2%	8.2%	0.0%	8.7%	1.3%	0.0%	2.2%	5.2%	0.4%	3.0%	0.0%	13.0%	8.2%	5.2%	10.4%	1.3%	0.0%	0.0%
121	0.4%	0.4%	0.0%	0.9%	4.8%	2.6%	5.6%	0.0%	0.9%	25.5%	0.9%	0.4%	0.4%	30.7%	23.8%	0.9%	0.0%	1.3%	0.0%	0.4%
122	23.4%	0.9%	4.3%	10.0%	0.0%	1.3%	2.6%	0.9%	9.5%	3.0%	0.4%	2.6%	0.0%	14.7%	11.3%	5.2%	9.1%	0.9%	0.0%	0.0%
123	2.6%	3.9%	0.0%	0.0%	0.4%	0.0%	0.0%	30.0%	0.0%	37.0%	2.6%	0.0%	1.3%	0.4%	0.9%	0.9%	3.0%	13.0%	3.9%	0.0%
124	8.7%	0.0%	8.7%	0.9%	0.0%	4.8%	0.0%	0.0%	2.6%	0.0%	0.0%	3.0%	7.4%	4.8%	1.7%	34.8%	21.7%	0.9%	0.0%	0.0%
125	39.6%	0.0%	0.5%	0.0%	0.0%	0.5%	4.8%	0.5%	0.0%	1.9%	1.4%	0.0%	33.3%	7.7%	0.5%	1.0%	4.3%	3.9%	0.0%	0.0%

126	20.8%	0.0%	15.0%	18.4%	0.0%	1.4%	1.0%	0.5%	1.0%	7.7%	0.5%	0.5%	10.6%	6.8%	5.8%	4.8%	3.4%	1.9%	0.0%	0.0%
127	14.3%	0.0%	22.9%	28.1%	0.0%	1.7%	1.7%	0.4%	0.4%	0.9%	0.0%	1.3%	0.0%	17.7%	2.2%	2.6%	3.9%	1.3%	0.4%	0.0%
128	72.3%	3.0%	0.0%	0.0%	0.0%	0.0%	0.0%	0.4%	0.0%	1.3%	0.0%	1.7%	0.0%	0.0%	0.0%	17.3%	1.7%	2.2%	0.0%	0.0%
129	0.4%	0.0%	0.9%	0.4%	0.4%	1.3%	0.0%	8.7%	0.0%	1.3%	17.3%	0.0%	0.0%	0.0%	0.0%	5.2%	54.1%	9.5%	0.0%	0.4%
130	29.0%	0.0%	6.5%	15.2%	0.0%	3.0%	1.7%	0.0%	3.9%	2.6%	0.9%	3.0%	0.0%	20.3%	6.1%	3.0%	4.3%	0.4%	0.0%	0.0%
131	52.8%	1.3%	2.2%	0.4%	2.6%	2.6%	2.6%	0.9%	0.0%	3.0%	0.4%	0.0%	0.0%	6.9%	8.2%	6.1%	5.6%	2.6%	1.7%	0.0%
132	0.0%	0.0%	0.0%	0.0%	55.0%	0.0%	0.0%	1.3%	0.0%	3.5%	0.9%	0.0%	0.0%	0.0%	0.0%	0.0%	0.0%	0.4%	0.4%	38.5%
133	3.9%	8.7%	0.0%	0.0%	0.0%	0.9%	0.0%	4.8%	0.9%	0.0%	0.0%	22.1%	0.0%	0.0%	0.0%	33.3%	14.7%	10.8%	0.0%	0.0%
134	3.9%	0.9%	8.2%	8.7%	0.0%	3.0%	8.7%	0.4%	8.2%	0.0%	0.0%	0.9%	0.0%	10.0%	41.6%	1.7%	3.0%	0.4%	0.0%	0.4%
135	2.2%	0.4%	0.0%	0.0%	0.9%	0.0%	0.4%	11.3%	0.0%	62.3%	2.2%	0.0%	0.0%	0.0%	3.0%	1.3%	4.3%	11.7%	0.0%	0.0%
136	0.0%	0.0%	0.0%	0.0%	0.0%	0.0%	0.0%	76.2%	0.0%	3.5%	1.3%	0.0%	0.0%	0.0%	0.0%	0.0%	1.7%	17.3%	0.0%	0.0%
137	36.4%	0.0%	0.0%	0.9%	0.0%	15.6%	6.5%	0.0%	0.0%	0.0%	0.0%	1.3%	0.0%	4.3%	19.9%	13.0%	2.2%	0.0%	0.0%	0.0%
138	9.5%	1.3%	1.3%	0.4%	0.0%	43.7%	12.6%	0.0%	0.9%	0.0%	0.0%	4.3%	0.4%	3.0%	1.3%	14.7%	5.2%	0.4%	0.0%	0.9%
139	0.4%	0.9%	0.0%	0.0%	0.0%	0.0%	2.6%	0.0%	0.0%	93.5%	0.9%	0.0%	0.0%	0.4%	0.0%	0.0%	0.0%	0.4%	0.9%	0.0%
140	0.0%	0.0%	0.0%	0.0%	0.4%	0.0%	0.0%	11.3%	0.0%	81.4%	2.6%	0.0%	0.0%	0.0%	0.0%	0.0%	0.0%	4.3%	0.0%	0.0%
141	48.5%	0.4%	1.7%	6.5%	0.0%	3.5%	0.4%	0.0%	0.0%	0.9%	0.0%	10.0%	0.0%	1.7%	0.4%	19.0%	6.5%	0.4%	0.0%	0.0%
142	0.4%	0.0%	0.0%	0.0%	0.4%	0.0%	0.0%	16.0%	0.0%	28.1%	0.9%	0.0%	0.0%	0.0%	0.0%	0.0%	0.0%	54.1%	0.0%	0.0%
143	1.3%	0.0%	0.0%	0.0%	0.0%	0.0%	0.0%	16.5%	0.0%	1.7%	0.0%	0.0%	0.0%	0.0%	0.0%	0.4%	0.4%	79.7%	0.0%	0.0%
144	0.0%	0.0%	0.0%	0.0%	86.5%	0.0%	0.0%	1.0%	0.0%	2.9%	0.0%	0.0%	7.2%	0.0%	0.0%	0.0%	0.0%	1.4%	0.0%	1.0%
145	0.9%	0.0%	4.3%	79.7%	8.7%	0.0%	0.0%	0.0%	0.0%	0.0%	0.0%	0.0%	0.0%	4.8%	0.0%	1.3%	0.0%	0.4%	0.0%	0.0%
146	73.6%	0.0%	0.0%	0.0%	0.0%	0.4%	0.0%	3.0%	0.0%	9.5%	0.9%	0.0%	0.0%	0.0%	0.0%	0.4%	1.7%	10.4%	0.0%	0.0%
147	83.5%	0.0%	0.0%	0.0%	0.0%	0.0%	0.0%	0.0%	0.0%	0.9%	0.4%	6.5%	0.0%	0.0%	0.0%	4.3%	3.5%	0.9%	0.0%	0.0%
148	0.0%	0.0%	94.4%	4.3%	0.0%	0.4%	0.0%	0.0%	0.0%	0.0%	0.0%	0.4%	0.0%	0.0%	0.0%	0.0%	0.0%	0.0%	0.0%	0.4%
149	11.7%	0.0%	0.0%	0.0%	0.0%	10.0%	0.9%	8.7%	0.0%	0.4%	0.9%	3.5%	0.0%	0.4%	0.4%	19.0%	35.9%	8.2%	0.0%	0.0%
150	68.4%	2.2%	0.4%	0.0%	0.0%	0.0%	0.4%	4.8%	0.0%	0.9%	0.0%	0.4%	0.9%	0.0%	0.0%	14.3%	2.6%	4.8%	0.0%	0.0%
151	18.2%	1.7%	9.5%	0.4%	0.0%	5.6%	0.4%	3.5%	0.0%	7.8%	1.3%	1.3%	2.2%	0.9%	0.4%	17.3%	13.4%	16.0%	0.0%	0.0%
152	0.4%	0.4%	77.5%	8.7%	0.0%	0.0%	4.8%	0.0%	0.0%	2.6%	0.0%	3.0%	0.0%	0.4%	0.0%	0.0%	0.0%	1.3%	0.0%	0.9%
153	6.5%	0.0%	0.0%	0.4%	0.0%	0.4%	0.0%	0.0%	0.0%	0.4%	0.0%	0.4%	89.1%	0.0%	0.0%	1.7%	0.4%	0.4%	0.0%	0.0%
154	13.4%	0.0%	19.5%	20.8%	0.0%	9.1%	2.2%	0.4%	2.2%	1.3%	0.0%	0.4%	1.3%	5.6%	3.5%	10.8%	5.2%	4.3%	0.0%	0.0%
155	0.4%	0.0%	0.0%	0.0%	0.0%	0.0%	0.0%	71.0%	0.0%	3.5%	3.0%	0.0%	0.0%	0.0%	0.0%	0.0%	1.3%	20.8%	0.0%	0.0%
156	19.9%	0.0%	0.0%	0.0%	0.0%	0.0%	0.0%	0.0%	0.0%	0.9%	0.4%	0.0%	0.0%	0.0%	0.0%	45.9%	32.0%	0.9%	0.0%	0.0%
157	0.0%	0.0%	0.0%	0.0%	0.0%	12.6%	1.3%	0.0%	1.7%	0.9%	0.0%	0.0%	0.4%	1.7%	76.6%	2.6%	1.7%	0.4%	0.0%	0.0%
158	32.9%	2.2%	0.0%	0.0%	0.0%	2.2%	0.9%	2.2%	0.0%	31.6%	2.2%	0.0%	2.2%	4.3%	6.1%	3.5%	3.9%	4.8%	0.4%	0.9%
159	0.0%	0.0%	0.0%	0.0%	0.4%	0.0%	0.0%	0.0%	0.0%	95.2%	4.3%	0.0%	0.0%	0.0%	0.0%	0.0%	0.0%	0.0%	0.0%	0.0%
160	2.2%	0.0%	0.0%	0.0%	0.0%	0.0%	0.0%	10.8%	0.0%	6.5%	0.0%	0.0%	0.0%	0.0%	0.0%	0.4%	1.7%	78.4%	0.0%	0.0%
161	94.8%	0.4%	0.0%	0.0%	0.0%	0.9%	0.0%	0.0%	0.0%	0.0%	0.0%	0.0%	0.0%	0.0%	0.0%	2.2%	1.7%	0.0%	0.0%	0.0%
162	0.0%	0.0%	0.0%	0.0%	2.6%	0.0%	0.0%	6.9%	0.4%	55.8%	32.5%	0.0%	0.0%	0.0%	0.4%	0.0%	0.0%	0.0%	0.0%	1.3%
163	0.0%	0.0%	0.0%	0.0%	70.6%	0.0%	0.0%	0.0%	0.0%	10.4%	0.0%	0.0%	0.0%	0.0%	0.0%	0.0%	0.0%	10.0%	0.0%	9.1%
164	0.0%	0.0%	0.0%	0.0%	0.0%	0.0%	27.7%	0.0%	0.0%	0.0%	0.0%	50.6%	0.0%	0.0%	0.0%	20.8%	0.4%	0.0%	0.0%	0.4%
165	0.4%	0.0%	0.0%	0.0%	64.9%	0.0%	0.0%	1.7%	0.0%	29.0%	0.0%	0.0%	0.0%	0.0%	0.0%	0.0%	0.0%	3.9%	0.0%	0.0%
166	0.0%	0.0%	0.0%	0.0%	0.0%	0.0%	0.0%	6.1%	0.0%	2.2%	78.8%	0.0%	0.0%	0.0%	0.0%	0.0%	0.0%	13.0%	0.0%	0.0%
167	0.0%	0.0%	0.0%	0.4%	0.0%	0.0%	0.4%	0.0%	0.0%	0.0%	0.0%	6.1%	0.0%	92.6%	0.4%	0.0%	0.0%	0.0%	0.0%	0.0%
168	6.9%	0.4%	0.0%	0.0%	0.0%	91.3%	0.0%	0.0%	0.0%	0.0%	0.0%	0.0%	0.0%	0.0%	0.0%	0.4%	0.9%	0.0%	0.0%	0.0%
169	0.0%	0.0%	0.0%	0.0%	0.0%	0.0%	0.0%	0.0%	97.4%	0.0%	0.0%	0.0%	0.0%	2.2%	0.0%	0.0%	0.4%	0.0%	0.0%	0.0%
170	0.0%	0.0%	0.0%	100.0%	0.0%	0.0%	0.0%	0.0%	0.0%	0.0%	0.0%	0.0%	0.0%	0.0%	0.0%	0.0%	0.0%	0.0%	0.0%	0.0%
171	3.9%	0.0%	0.0%	2.2%	25.5%	0.0%	1.3%	0.0%	0.0%	52.8%	0.4%	0.0%	0.0%	0.0%	0.0%	0.4%	0.0%	0.0%	0.9%	12.6%

172	77.5%	7.8%	0.0%	0.0%	0.0%	0.0%	0.0%	0.0%	0.0%	0.0%	0.0%	0.0%	0.0%	0.0%	0.0%	6.9%	3.0%	4.8%	0.0%	0.0%
173	0.0%	0.0%	0.0%	0.0%	0.0%	100.0%	0.0%	0.0%	0.0%	0.0%	0.0%	0.0%	0.0%	0.0%	0.0%	0.0%	0.0%	0.0%	0.0%	0.0%
174	0.0%	0.0%	0.0%	0.0%	0.0%	0.0%	0.0%	0.0%	0.0%	0.0%	0.0%	0.0%	0.0%	98.7%	1.3%	0.0%	0.0%	0.0%	0.0%	0.0%
175	0.0%	0.0%	0.0%	100.0%	0.0%	0.0%	0.0%	0.0%	0.0%	0.0%	0.0%	0.0%	0.0%	0.0%	0.0%	0.0%	0.0%	0.0%	0.0%	0.0%
176	0.0%	0.0%	0.0%	0.0%	0.0%	0.0%	0.0%	0.0%	0.0%	0.0%	0.0%	0.0%	0.0%	0.0%	100.0%	0.0%	0.0%	0.0%	0.0%	0.0%
177	100.0%	0.0%	0.0%	0.0%	0.0%	0.0%	0.0%	0.0%	0.0%	0.0%	0.0%	0.0%	0.0%	0.0%	0.0%	0.0%	0.0%	0.0%	0.0%	0.0%
178	13.0%	16.1%	0.0%	0.0%	4.8%	0.0%	0.9%	7.0%	0.0%	16.1%	0.9%	0.0%	0.0%	0.4%	0.0%	0.9%	12.2%	14.3%	10.4%	3.0%
179	4.3%	0.0%	0.0%	0.0%	0.0%	94.8%	0.0%	0.0%	0.0%	0.0%	0.0%	0.0%	0.0%	0.0%	0.0%	0.0%	0.4%	0.4%	0.0%	0.0%
180	60.2%	1.3%	0.0%	0.0%	0.0%	2.6%	0.0%	0.0%	0.0%	0.4%	0.0%	0.0%	0.0%	0.0%	0.0%	18.6%	0.4%	16.5%	0.0%	0.0%
181	48.1%	0.0%	0.0%	0.9%	4.8%	0.9%	2.2%	12.1%	0.0%	9.1%	5.6%	0.0%	0.0%	3.0%	2.6%	2.6%	1.3%	3.9%	0.0%	3.0%
182	18.6%	0.9%	0.0%	0.0%	0.0%	61.9%	0.0%	0.9%	0.0%	10.8%	2.6%	0.0%	0.0%	0.0%	0.0%	1.7%	1.3%	1.3%	0.0%	0.0%
183	0.0%	0.0%	0.0%	0.0%	88.3%	0.0%	0.0%	0.4%	0.0%	4.3%	0.0%	0.0%	0.0%	0.0%	0.0%	0.0%	0.0%	0.0%	0.0%	6.9%
184	55.8%	0.0%	0.0%	0.0%	0.0%	5.6%	0.0%	0.0%	0.0%	1.7%	0.9%	0.0%	0.0%	0.0%	0.4%	14.3%	20.8%	0.4%	0.0%	0.0%
185	32.0%	0.9%	0.9%	4.8%	0.0%	1.7%	3.0%	0.4%	0.9%	6.9%	0.9%	0.9%	0.0%	3.5%	10.4%	30.3%	1.3%	1.3%	0.0%	0.0%
186	0.9%	0.4%	0.0%	0.9%	0.0%	85.7%	0.4%	0.0%	0.0%	0.4%	0.4%	0.0%	0.0%	0.9%	2.6%	3.9%	3.5%	0.0%	0.0%	0.0%
187	6.1%	0.4%	0.9%	9.6%	1.7%	5.2%	20.9%	0.4%	0.9%	1.7%	0.0%	0.4%	0.0%	10.0%	18.7%	5.7%	3.0%	4.8%	0.9%	8.7%
188	6.1%	4.8%	0.0%	0.0%	63.5%	3.0%	0.0%	3.0%	0.0%	2.2%	0.9%	3.0%	0.9%	0.0%	0.0%	6.1%	1.7%	4.8%	0.0%	0.0%
189	1.3%	0.0%	46.3%	6.1%	0.0%	3.0%	0.9%	0.0%	0.9%	0.0%	0.0%	7.4%	0.4%	3.9%	1.3%	16.9%	11.3%	0.4%	0.0%	0.0%
190	23.9%	0.0%	21.7%	13.5%	0.0%	5.7%	0.9%	0.0%	3.5%	2.2%	0.0%	2.6%	6.5%	3.0%	2.2%	7.8%	3.5%	3.0%	0.0%	0.0%
191	34.3%	0.0%	6.5%	19.6%	0.0%	4.3%	6.1%	0.0%	0.0%	0.4%	0.4%	1.3%	6.1%	6.1%	3.0%	5.2%	4.8%	1.7%	0.0%	0.0%
192	4.8%	0.0%	3.5%	6.5%	0.0%	3.5%	9.6%	1.7%	0.0%	24.8%	2.6%	0.4%	0.9%	23.5%	12.6%	0.9%	3.5%	0.9%	0.0%	0.4%
193	2.9%	4.3%	0.0%	1.0%	0.0%	1.4%	5.8%	1.0%	7.7%	5.3%	0.0%	0.5%	0.0%	25.6%	30.4%	12.6%	1.0%	0.5%	0.0%	0.0%
194	9.1%	0.0%	9.1%	12.1%	0.0%	1.3%	1.7%	0.0%	0.9%	3.0%	0.0%	0.4%	0.4%	51.5%	6.5%	1.7%	0.9%	1.3%	0.0%	0.0%
195	1.3%	0.0%	0.9%	0.9%	0.0%	0.4%	4.3%	0.0%	8.7%	10.0%	0.9%	0.0%	0.0%	23.4%	42.0%	1.7%	5.6%	0.0%	0.0%	0.0%
196	0.0%	0.0%	0.0%	0.0%	0.4%	0.0%	0.0%	11.7%	0.0%	54.3%	16.5%	0.0%	0.0%	0.4%	0.0%	0.0%	0.0%	3.9%	12.6%	0.0%
197	8.2%	0.9%	0.9%	3.5%	0.0%	1.3%	2.6%	2.2%	0.9%	33.3%	5.2%	3.5%	0.4%	10.0%	5.2%	3.0%	4.3%	14.7%	0.0%	0.0%
198	8.7%	0.0%	12.1%	7.4%	0.9%	2.2%	44.2%	0.0%	0.4%	2.2%	0.4%	1.3%	0.0%	12.6%	0.4%	3.0%	2.6%	0.0%	0.0%	1.7%
199	0.0%	0.0%	0.0%	0.0%	2.2%	0.0%	0.4%	0.0%	0.0%	87.9%	0.4%	0.0%	0.0%	0.4%	8.7%	0.0%	0.0%	0.0%	0.0%	0.0%
200	4.3%	0.9%	0.4%	0.0%	0.0%	0.0%	0.0%	64.1%	0.4%	3.0%	0.9%	0.0%	0.0%	7.4%	1.3%	0.9%	1.7%	14.7%	0.0%	0.0%
201	9.5%	0.0%	37.2%	39.8%	0.4%	0.9%	1.7%	0.4%	0.4%	0.0%	0.0%	1.3%	0.0%	4.8%	0.9%	0.0%	0.9%	1.3%	0.0%	0.4%
202	40.3%	0.4%	4.3%	0.0%	0.0%	16.5%	0.9%	0.0%	0.0%	2.6%	1.3%	1.3%	0.0%	3.0%	4.8%	22.9%	1.3%	0.4%	0.0%	0.0%
203	0.0%	0.0%	0.0%	0.4%	0.0%	0.0%	0.0%	0.0%	0.0%	0.0%	0.0%	0.0%	0.0%	99.6%	0.0%	0.0%	0.0%	0.0%	0.0%	0.0%
204	3.9%	0.0%	4.3%	58.0%	0.0%	0.0%	0.9%	0.0%	0.9%	0.9%	0.0%	1.7%	0.0%	26.0%	1.7%	0.4%	0.9%	0.4%	0.0%	0.0%
205	2.2%	0.0%	0.9%	0.9%	0.0%	0.9%	6.5%	0.0%	0.4%	1.7%	0.0%	0.4%	3.5%	7.8%	74.0%	0.9%	0.0%	0.0%	0.0%	0.0%
206	2.6%	70.6%	0.0%	0.0%	0.0%	0.4%	4.3%	0.4%	0.0%	0.0%	0.0%	1.3%	0.0%	0.0%	0.0%	19.5%	0.0%	0.0%	0.0%	0.9%
207	0.4%	0.0%	0.0%	0.0%	81.4%	0.0%	0.0%	0.9%	0.0%	16.5%	0.0%	0.0%	0.0%	0.0%	0.0%	0.0%	0.0%	0.9%	0.0%	0.0%
208	7.8%	0.0%	22.1%	22.5%	0.0%	3.0%	3.0%	0.4%	1.3%	0.0%	0.4%	1.7%	0.0%	25.1%	6.5%	3.0%	1.7%	0.9%	0.0%	0.4%
209	3.0%	0.0%	0.0%	0.0%	0.4%	1.3%	0.0%	10.8%	0.0%	7.8%	0.9%	0.0%	0.0%	0.9%	3.0%	4.3%	31.2%	35.9%	0.0%	0.4%
210	1.3%	0.9%	0.0%	0.0%	94.8%	0.0%	0.0%	0.0%	0.0%	0.0%	0.0%	0.0%	0.0%	0.0%	0.0%	0.4%	0.0%	1.3%	0.0%	1.3%
211	19.9%	4.8%	0.4%	12.1%	0.4%	3.9%	0.9%	4.3%	1.7%	11.3%	2.6%	2.2%	0.0%	3.9%	2.2%	10.8%	10.4%	8.2%	0.0%	0.0%
212	7.4%	0.0%	13.9%	39.0%	0.0%	2.2%	0.9%	0.0%	0.9%	0.0%	0.0%	4.3%	0.0%	14.7%	3.5%	12.6%	0.9%	0.0%	0.0%	0.0%
213	0.0%	2.2%	0.0%	0.0%	79.2%	0.0%	3.5%	0.0%	0.0%	8.2%	0.4%	0.0%	0.0%	0.0%	0.0%	0.4%	0.4%	0.4%	0.0%	5.2%
214	74.0%	2.6%	0.0%	0.0%	0.0%	4.8%	0.0%	0.4%	0.0%	0.0%	0.0%	0.0%	0.0%	0.0%	0.0%	13.9%	3.9%	0.4%	0.0%	0.0%
215	3.9%	0.4%	36.7%	7.0%	0.0%	9.6%	0.4%	0.0%	0.4%	0.4%	0.0%	3.9%	8.3%	1.7%	0.4%	17.5%	8.7%	0.4%	0.0%	0.0%
216	24.2%	0.0%	13.0%	13.9%	0.0%	0.4%	0.9%	0.9%	2.6%	0.9%	0.0%	0.4%	26.8%	3.5%	1.3%	5.6%	4.8%	0.9%	0.0%	0.0%
217	21.2%	0.4%	14.7%	18.6%	0.0%	5.2%	1.7%	0.4%	1.3%	0.4%	0.4%	1.7%	5.2%	10.0%	4.8%	5.6%	6.1%	1.7%	0.0%	0.4%

218	19.6%	2.2%	1.7%	3.5%	0.4%	1.3%	2.6%	2.6%	0.0%	23.5%	0.9%	4.3%	3.5%	7.0%	0.4%	15.2%	1.7%	9.6%	0.0%	0.0%
219	8.7%	1.7%	0.9%	0.4%	0.0%	2.2%	0.4%	0.9%	3.0%	35.5%	3.0%	1.7%	0.0%	8.7%	22.5%	0.4%	3.5%	5.6%	0.9%	0.0%
220	32.6%	0.0%	4.8%	8.3%	0.0%	3.9%	3.0%	1.7%	1.7%	3.0%	1.3%	0.9%	0.9%	17.4%	3.9%	6.5%	6.5%	2.6%	0.9%	0.0%
221	20.4%	2.6%	0.9%	3.0%	0.4%	0.4%	2.6%	2.2%	0.4%	30.4%	2.2%	0.4%	0.9%	7.8%	15.2%	3.0%	2.6%	2.6%	1.3%	0.4%
222	0.0%	0.9%	0.9%	0.0%	7.4%	0.0%	0.0%	0.0%	0.0%	2.6%	0.0%	0.0%	0.0%	0.4%	0.0%	0.0%	0.0%	0.0%	86.6%	1.3%
223	6.9%	0.0%	4.8%	9.5%	0.0%	0.0%	4.8%	0.4%	1.3%	4.3%	0.0%	1.7%	0.0%	38.1%	21.6%	3.0%	2.6%	0.4%	0.0%	0.4%
224	23.8%	0.0%	1.7%	12.6%	0.0%	3.5%	2.6%	0.0%	3.0%	2.6%	0.4%	1.3%	1.7%	27.3%	4.3%	7.4%	7.4%	0.4%	0.0%	0.0%
225	11.3%	1.7%	0.0%	3.9%	0.0%	2.2%	3.9%	7.4%	0.0%	30.7%	5.6%	1.7%	0.4%	11.7%	1.7%	9.1%	2.2%	5.6%	0.0%	0.9%
226	6.4%	9.1%	1.8%	8.6%	0.0%	3.2%	1.8%	0.9%	1.4%	10.9%	2.7%	1.4%	1.4%	31.8%	10.0%	1.4%	4.5%	2.7%	0.0%	0.0%
227	17.4%	0.0%	8.7%	7.8%	0.5%	6.4%	2.3%	0.0%	2.3%	5.9%	0.5%	2.7%	5.0%	10.0%	4.6%	16.4%	8.7%	0.9%	0.0%	0.0%
228	23.5%	0.9%	10.9%	1.8%	0.5%	6.3%	9.0%	0.0%	0.5%	1.4%	0.5%	2.7%	10.0%	6.8%	2.3%	14.9%	7.2%	0.9%	0.0%	0.0%
229	6.1%	0.9%	7.4%	9.6%	0.4%	4.8%	3.5%	2.6%	0.4%	21.4%	5.7%	3.1%	0.4%	5.7%	2.6%	9.2%	6.6%	7.9%	0.9%	0.9%
230	39.8%	0.4%	3.0%	3.9%	0.0%	3.5%	2.2%	3.9%	2.2%	6.1%	0.0%	0.4%	0.9%	4.8%	8.7%	11.7%	6.1%	2.6%	0.0%	0.0%
231	6.1%	0.0%	9.1%	46.8%	0.0%	7.8%	0.9%	0.0%	0.9%	1.3%	0.0%	0.9%	2.6%	11.3%	4.3%	0.9%	5.6%	1.7%	0.0%	0.0%
232	1.3%	0.0%	0.0%	0.0%	11.7%	0.0%	1.3%	30.7%	0.0%	37.2%	1.3%	0.0%	0.0%	3.0%	0.4%	0.0%	2.2%	10.4%	0.0%	0.4%
233	3.9%	2.6%	2.2%	64.1%	0.0%	2.6%	2.2%	0.4%	0.9%	3.0%	0.4%	2.6%	0.0%	9.5%	2.2%	0.9%	1.3%	1.3%	0.0%	0.0%
234	1.3%	0.0%	0.0%	0.4%	0.0%	0.9%	1.7%	0.0%	4.3%	0.0%	0.0%	0.4%	0.4%	19.5%	68.0%	1.3%	1.7%	0.0%	0.0%	0.0%
235	0.0%	0.0%	0.0%	0.0%	3.5%	0.0%	0.0%	0.4%	0.0%	76.2%	16.5%	0.0%	0.0%	0.9%	0.0%	0.0%	0.0%	0.0%	0.9%	1.7%
236	0.0%	0.0%	0.0%	0.0%	0.0%	0.0%	0.0%	0.0%	0.0%	0.0%	0.0%	0.0%	0.0%	0.0%	100.0%	0.0%	0.0%	0.0%	0.0%	0.0%
237	3.0%	0.0%	8.7%	5.2%	0.0%	5.7%	1.3%	0.0%	3.5%	0.0%	0.0%	1.3%	0.0%	10.9%	57.0%	0.9%	1.7%	0.9%	0.0%	0.0%
238	2.2%	0.0%	0.0%	0.9%	0.4%	0.0%	0.4%	31.7%	4.8%	15.7%	16.1%	0.0%	0.0%	2.2%	3.9%	0.0%	5.7%	15.7%	0.0%	0.4%
239	50.4%	0.0%	0.0%	0.0%	0.4%	13.0%	0.0%	9.6%	0.0%	20.9%	0.0%	0.0%	0.0%	0.0%	0.0%	1.3%	0.4%	3.9%	0.0%	0.0%
240	9.1%	61.6%	1.0%	0.5%	0.5%	1.0%	0.5%	0.5%	0.5%	7.1%	1.0%	0.0%	0.0%	2.5%	2.5%	1.5%	6.1%	3.0%	0.5%	0.5%
241	15.2%	0.0%	1.8%	0.9%	0.0%	8.5%	0.9%	1.3%	1.3%	1.8%	0.4%	0.4%	0.0%	4.9%	4.9%	11.6%	45.5%	0.4%	0.0%	0.0%
242	23.5%	0.5%	4.5%	0.9%	0.9%	5.0%	1.8%	0.9%	0.0%	10.9%	0.5%	0.5%	3.6%	5.0%	14.9%	13.6%	5.9%	5.9%	0.0%	1.4%
243	22.3%	0.0%	2.3%	3.6%	0.0%	15.0%	1.4%	0.5%	0.9%	5.5%	0.5%	0.9%	13.6%	6.8%	5.5%	10.9%	5.5%	5.0%	0.0%	0.0%
244	18.6%	0.0%	9.5%	6.0%	0.0%	2.0%	5.0%	0.0%	3.0%	2.0%	0.0%	2.0%	20.1%	6.5%	11.6%	7.0%	5.5%	1.0%	0.0%	0.0%
245	11.5%	1.5%	7.0%	3.5%	3.5%	30.0%	3.5%	1.0%	0.5%	0.5%	0.0%	0.0%	9.0%	7.0%	2.5%	7.0%	6.0%	1.0%	0.0%	5.0%
246	18.1%	0.5%	7.0%	10.1%	0.0%	7.5%	1.5%	2.5%	2.0%	3.5%	0.5%	2.5%	16.6%	6.5%	9.0%	4.0%	6.5%	1.5%	0.0%	0.0%
247	4.1%	1.8%	11.0%	1.8%	0.5%	2.7%	0.9%	5.9%	0.0%	34.2%	0.9%	5.5%	3.7%	2.7%	1.4%	3.7%	4.1%	15.1%	0.0%	0.0%
248	5.8%	0.0%	38.8%	4.9%	0.0%	12.1%	0.4%	0.0%	0.9%	8.9%	0.0%	4.0%	11.6%	0.4%	0.9%	8.0%	1.8%	0.9%	0.0%	0.4%
249	16.7%	0.5%	4.7%	3.6%	0.0%	10.9%	1.0%	0.0%	2.6%	2.6%	0.5%	2.6%	24.5%	1.6%	5.2%	13.5%	7.8%	1.6%	0.0%	0.0%
250	16.5%	0.0%	12.4%	25.8%	0.0%	9.3%	1.0%	1.0%	0.5%	0.5%	0.5%	4.6%	1.5%	8.8%	2.1%	11.3%	3.6%	0.5%	0.0%	0.0%
251	3.6%	0.0%	0.5%	0.0%	2.5%	3.6%	0.5%	7.6%	0.0%	56.9%	4.6%	0.0%	1.5%	3.0%	1.5%	3.6%	7.1%	3.0%	0.0%	0.5%
252	33.5%	2.2%	0.9%	0.0%	0.0%	13.0%	0.0%	0.0%	0.0%	0.0%	0.0%	0.0%	0.9%	0.0%	0.0%	45.2%	1.3%	3.0%	0.0%	0.0%
253	3.0%	0.0%	16.9%	36.8%	0.0%	2.2%	0.4%	0.0%	0.0%	18.2%	0.4%	0.0%	1.3%	16.5%	0.9%	0.9%	1.3%	0.9%	0.0%	0.4%
254	10.4%	0.0%	2.6%	2.6%	0.4%	1.7%	3.9%	7.8%	0.9%	8.7%	0.0%	0.9%	4.8%	4.8%	20.8%	2.2%	15.6%	12.1%	0.0%	0.0%
255	0.0%	0.0%	0.0%	0.0%	0.0%	0.0%	0.0%	0.0%	0.0%	0.0%	0.0%	0.0%	0.0%	0.0%	0.0%	0.0%	0.0%	0.0%	100.0%	0.0%
256	0.0%	0.0%	0.0%	0.0%	76.6%	0.0%	0.0%	0.0%	0.0%	0.4%	0.0%	0.0%	0.0%	0.0%	0.0%	0.0%	0.0%	0.4%	0.0%	22.5%
257	15.6%	0.9%	36.8%	26.8%	0.0%	2.2%	0.9%	0.4%	0.4%	0.4%	0.4%	1.7%	0.0%	3.9%	1.3%	3.0%	3.9%	0.4%	0.4%	0.4%
258	10.0%	15.2%	0.0%	0.4%	0.4%	0.9%	0.9%	8.3%	0.4%	42.2%	0.4%	0.4%	0.0%	1.3%	0.4%	0.9%	0.4%	16.1%	1.3%	0.0%
259	22.5%	32.0%	0.0%	0.0%	0.0%	0.0%	2.2%	0.0%	0.0%	5.2%	8.7%	2.2%	0.0%	7.4%	0.0%	2.6%	16.9%	0.0%	0.0%	0.4%
260	0.0%	0.0%	0.0%	0.0%	0.0%	0.0%	0.0%	0.0%	0.0%	0.0%	0.0%	0.0%	0.0%	0.0%	0.0%	27.7%	72.3%	0.0%	0.0%	0.0%
261	23.8%	0.9%	1.7%	15.2%	0.0%	1.3%	1.7%	0.0%	0.9%	8.7%	0.4%	0.4%	0.0%	19.9%	17.3%	2.2%	3.9%	1.3%	0.4%	0.0%
262	0.0%	0.4%	0.0%	0.0%	0.0%	0.0%	0.4%	0.0%	0.0%	0.4%	0.0%	0.0%	0.0%	0.4%	97.0%	0.0%	0.0%	1.3%	0.0%	0.0%
263	0.0%	0.0%	0.0%	0.0%	0.0%	0.0%	0.0%	68.7%	0.0%	17.0%	13.0%	0.0%	0.0%	0.9%	0.0%	0.0%	0.0%	0.4%	0.0%	0.0%

264	0.4%	0.0%	83.5%	6.1%	0.0%	0.9%	0.0%	0.0%	0.0%	0.0%	0.0%	3.0%	0.0%	0.0%	0.0%	1.3%	4.8%	0.0%	0.0%	0.0%
265	43.5%	0.0%	5.7%	9.1%	0.0%	10.9%	1.7%	0.4%	1.7%	4.8%	0.4%	0.4%	0.0%	9.6%	3.0%	3.5%	4.3%	0.9%	0.0%	0.0%
266	0.0%	0.0%	0.0%	0.0%	0.9%	0.0%	0.0%	8.7%	0.0%	26.4%	64.1%	0.0%	0.0%	0.0%	0.0%	0.0%	0.0%	0.0%	0.0%	0.0%
267	0.4%	0.0%	0.0%	0.0%	0.0%	1.7%	12.1%	0.0%	45.0%	0.4%	0.4%	0.0%	0.0%	13.0%	16.5%	0.0%	0.0%	0.0%	9.1%	1.3%
268	10.8%	0.0%	3.0%	11.7%	0.0%	6.1%	3.5%	1.7%	3.0%	4.3%	1.3%	0.9%	2.2%	19.9%	7.4%	9.1%	12.6%	1.7%	0.4%	0.4%
269	0.4%	0.4%	0.0%	0.4%	0.4%	0.0%	0.0%	30.3%	0.0%	20.3%	2.2%	0.0%	0.0%	0.4%	0.4%	0.0%	1.3%	43.3%	0.0%	0.0%
270	0.4%	0.0%	0.0%	90.0%	0.0%	0.0%	0.0%	0.0%	0.0%	0.0%	0.4%	0.0%	0.0%	8.7%	0.0%	0.0%	0.0%	0.4%	0.0%	0.0%
271	6.1%	3.9%	42.4%	17.7%	0.0%	0.9%	0.4%	3.0%	1.3%	0.9%	0.4%	4.3%	0.0%	3.9%	3.0%	3.0%	3.0%	5.6%	0.0%	0.0%
272	16.0%	1.7%	3.0%	4.3%	1.3%	2.6%	6.9%	0.9%	1.7%	6.1%	1.7%	1.7%	0.0%	15.2%	21.2%	6.9%	4.8%	1.7%	0.4%	1.7%
273	1.7%	0.0%	0.0%	0.0%	0.4%	0.0%	0.0%	2.2%	0.0%	87.9%	2.2%	0.0%	0.0%	0.0%	0.0%	0.4%	0.0%	5.2%	0.0%	0.0%
274	32.9%	0.9%	0.4%	18.6%	0.0%	0.4%	0.4%	11.7%	0.0%	0.9%	0.4%	3.9%	0.0%	0.9%	0.4%	3.9%	16.0%	8.2%	0.0%	0.0%
275	22.6%	0.0%	15.7%	12.2%	0.0%	7.0%	1.3%	0.4%	3.5%	3.9%	0.9%	3.5%	0.0%	13.0%	3.9%	6.1%	4.8%	1.3%	0.0%	0.0%
276	14.8%	3.9%	18.3%	11.3%	0.9%	5.2%	5.2%	0.4%	1.3%	1.3%	0.4%	0.9%	0.0%	10.0%	10.4%	3.5%	7.8%	3.0%	0.0%	1.3%
277	0.0%	0.0%	0.0%	0.0%	4.5%	0.0%	0.0%	1.3%	0.0%	88.4%	2.2%	0.0%	0.0%	0.0%	0.0%	0.0%	0.0%	3.6%	0.0%	0.0%
278	7.0%	0.0%	0.0%	1.9%	0.0%	0.5%	6.5%	2.8%	1.4%	15.9%	16.4%	1.4%	0.0%	9.3%	22.9%	3.3%	7.9%	2.8%	0.0%	0.0%
332	20.0%	0.0%	4.0%	12.9%	0.0%	3.6%	0.9%	0.0%	1.3%	0.9%	0.9%	6.2%	0.0%	5.8%	1.3%	19.1%	23.1%	0.0%	0.0%	0.0%
333	43.9%	0.0%	0.0%	0.0%	0.0%	0.0%	0.0%	0.0%	0.0%	13.9%	6.1%	0.0%	0.0%	0.0%	0.0%	0.4%	13.9%	21.7%	0.0%	0.0%
334	0.0%	0.0%	0.0%	0.4%	0.0%	0.0%	0.9%	0.0%	16.0%	0.0%	0.0%	0.0%	0.0%	4.8%	77.9%	0.0%	0.0%	0.0%	0.0%	0.0%
335	31.2%	0.0%	5.6%	13.0%	0.0%	3.9%	0.9%	0.0%	10.0%	0.4%	0.0%	0.0%	0.0%	13.0%	13.4%	3.0%	4.3%	1.3%	0.0%	0.0%
336	54.5%	0.0%	0.0%	0.4%	0.0%	0.4%	0.0%	0.0%	0.0%	0.4%	1.3%	0.4%	0.0%	0.0%	0.0%	20.8%	19.9%	1.7%	0.0%	0.0%
337	0.0%	0.0%	0.0%	0.0%	0.4%	0.0%	0.0%	4.8%	0.0%	91.8%	0.4%	0.0%	0.0%	0.9%	0.4%	0.0%	0.0%	1.3%	0.0%	0.0%
338	6.9%	0.0%	11.3%	21.2%	0.4%	0.4%	5.2%	0.0%	1.3%	0.4%	0.4%	26.8%	0.0%	3.0%	9.1%	5.2%	6.9%	1.3%	0.0%	0.0%
339	0.9%	0.0%	11.7%	85.7%	0.0%	0.0%	0.0%	0.0%	0.9%	0.0%	0.0%	0.0%	0.0%	0.9%	0.0%	0.0%	0.0%	0.0%	0.0%	0.0%
340	0.0%	0.0%	0.0%	0.0%	0.0%	0.0%	0.9%	0.0%	0.0%	0.0%	0.0%	0.0%	0.0%	5.6%	93.5%	0.0%	0.0%	0.0%	0.0%	0.0%
341	0.0%	0.0%	0.0%	0.0%	0.0%	0.4%	0.0%	0.4%	85.7%	0.0%	0.0%	0.4%	0.0%	0.4%	11.7%	0.0%	0.9%	0.0%	0.0%	0.0%
342	1.7%	0.4%	0.4%	1.7%	0.4%	0.0%	2.6%	7.4%	0.0%	38.5%	0.0%	0.0%	0.0%	7.8%	5.2%	0.4%	14.3%	18.6%	0.0%	0.4%
343	0.0%	0.0%	0.0%	0.0%	0.0%	0.0%	0.0%	60.6%	0.0%	5.6%	0.9%	0.0%	0.0%	6.1%	0.0%	0.0%	0.4%	26.4%	0.0%	0.0%
344	0.4%	0.0%	23.8%	72.7%	0.0%	0.9%	0.4%	0.0%	0.0%	0.0%	0.0%	0.9%	0.0%	0.9%	0.0%	0.0%	0.0%	0.0%	0.0%	0.0%
345	0.0%	0.0%	0.0%	0.0%	0.0%	0.0%	0.0%	0.0%	17.7%	0.0%	0.0%	0.0%	0.0%	7.8%	73.6%	0.9%	0.0%	0.0%	0.0%	0.0%
346	99.6%	0.0%	0.0%	0.0%	0.0%	0.0%	0.0%	0.0%	0.0%	0.0%	0.0%	0.0%	0.0%	0.0%	0.0%	0.0%	0.0%	0.4%	0.0%	0.0%
347	0.4%	0.0%	0.0%	0.0%	0.0%	0.0%	0.0%	0.0%	92.6%	0.0%	0.0%	0.0%	0.0%	0.0%	0.4%	0.0%	0.0%	6.5%	0.0%	0.0%
348	6.9%	0.0%	0.0%	0.4%	0.0%	54.1%	1.3%	0.0%	1.7%	13.4%	0.0%	1.3%	0.0%	2.6%	1.7%	9.1%	0.9%	0.4%	5.2%	0.9%
349	2.6%	0.0%	0.0%	1.3%	0.0%	0.0%	0.0%	5.2%	0.0%	70.1%	3.0%	0.0%	0.0%	0.9%	0.9%	0.0%	0.0%	11.7%	4.3%	0.0%
350	0.0%	0.0%	0.0%	0.0%	0.0%	0.0%	0.0%	8.7%	0.0%	90.0%	0.9%	0.0%	0.0%	0.0%	0.0%	0.0%	0.0%	0.4%	0.0%	0.0%
351	0.9%	0.0%	0.0%	0.0%	0.0%	0.0%	0.0%	6.9%	0.4%	0.0%	87.9%	0.0%	0.0%	0.0%	0.0%	0.0%	0.0%	3.9%	0.0%	0.0%
352	31.2%	0.0%	2.2%	8.7%	0.0%	0.9%	0.9%	0.4%	3.9%	0.0%	0.9%	4.8%	0.0%	22.9%	5.2%	10.4%	7.4%	0.4%	0.0%	0.0%
353	0.9%	0.4%	0.9%	2.2%	0.0%	0.0%	53.7%	0.0%	3.0%	0.9%	0.0%	0.9%	0.0%	13.0%	12.6%	4.8%	3.0%	0.0%	0.0%	3.9%
354	0.4%	0.0%	0.0%	0.0%	2.6%	2.6%	11.7%	0.0%	0.9%	10.0%	1.7%	0.0%	0.0%	23.8%	40.7%	0.4%	0.0%	0.0%	0.9%	4.3%
355	1.7%	0.0%	2.6%	2.2%	0.0%	42.0%	2.2%	0.0%	9.5%	0.0%	0.0%	9.1%	0.0%	15.6%	13.0%	1.7%	0.4%	0.0%	0.0%	0.0%
356	0.0%	1.3%	0.0%	0.0%	1.7%	0.0%	0.4%	13.4%	0.0%	54.1%	21.6%	0.0%	0.0%	0.0%	0.0%	0.0%	0.0%	3.9%	0.0%	3.5%
357	0.4%	0.0%	6.9%	0.0%	0.0%	1.7%	0.0%	0.0%	0.0%	0.0%	0.0%	6.9%	0.0%	1.7%	0.9%	59.7%	21.6%	0.0%	0.0%	0.0%
358	0.0%	0.0%	0.4%	93.5%	0.0%	0.0%	0.0%	0.0%	0.0%	0.0%	0.0%	6.1%	0.0%	0.0%	0.0%	0.0%	0.0%	0.0%	0.0%	0.0%
359	6.1%	0.0%	19.9%	46.8%	0.0%	2.6%	0.9%	0.0%	0.9%	0.0%	1.3%	3.0%	5.2%	10.0%	0.0%	2.2%	0.4%	0.9%	0.0%	0.0%
360	14.7%	0.0%	9.5%	43.7%	0.0%	0.9%	0.0%	0.4%	0.9%	0.4%	0.0%	0.4%	0.0%	25.1%	0.4%	1.7%	0.0%	1.7%	0.0%	0.0%
361	100.0%	0.0%	0.0%	0.0%	0.0%	0.0%	0.0%	0.0%	0.0%	0.0%	0.0%	0.0%	0.0%	0.0%	0.0%	0.0%	0.0%	0.0%	0.0%	0.0%

362	0.0%	0.0%	0.0%	0.0%	7.4%	0.0%	34.6%	0.0%	0.0%	0.0%	0.0%	0.0%	0.0%	0.4%	0.0%	0.0%	0.0%	0.0%	12.1%	45.5%
363	4.3%	0.0%	2.6%	4.3%	0.0%	0.9%	2.6%	0.0%	33.8%	0.0%	0.0%	0.4%	0.0%	12.6%	36.8%	0.4%	1.3%	0.0%	0.0%	0.0%
364	10.4%	1.3%	0.0%	0.4%	8.3%	0.0%	0.9%	2.2%	4.8%	18.7%	12.6%	0.4%	0.0%	8.7%	8.7%	1.7%	16.1%	4.3%	0.4%	0.0%
365	0.0%	0.0%	0.0%	0.0%	0.0%	0.0%	0.0%	3.5%	0.0%	90.4%	6.1%	0.0%	0.0%	0.0%	0.0%	0.0%	0.0%	0.0%	0.0%	0.0%
366	0.0%	0.0%	0.0%	0.0%	0.0%	0.0%	0.0%	0.0%	0.0%	1.3%	0.0%	0.0%	0.0%	22.2%	76.5%	0.0%	0.0%	0.0%	0.0%	0.0%
367	0.0%	0.0%	2.2%	2.2%	0.0%	0.0%	0.4%	0.0%	44.8%	0.0%	0.0%	2.2%	0.0%	31.3%	10.4%	4.3%	2.2%	0.0%	0.0%	0.0%
368	12.6%	0.0%	0.0%	0.4%	0.0%	0.9%	0.9%	0.0%	0.4%	10.0%	23.5%	0.0%	0.0%	3.0%	2.2%	10.9%	33.5%	1.7%	0.0%	0.0%
369	86.1%	0.0%	0.0%	0.0%	0.0%	0.0%	0.0%	0.0%	0.0%	0.0%	0.0%	0.0%	0.0%	0.0%	0.0%	13.9%	0.0%	0.0%	0.0%	0.0%
370	0.0%	0.0%	0.0%	0.0%	0.0%	0.0%	0.0%	0.0%	0.0%	0.0%	100.0%	0.0%	0.0%	0.0%	0.0%	0.0%	0.0%	0.0%	0.0%	0.0%
371	1.7%	0.0%	38.3%	11.3%	0.0%	0.0%	0.0%	0.0%	1.3%	0.0%	0.0%	37.8%	0.0%	1.7%	0.9%	6.1%	0.9%	0.0%	0.0%	0.0%
372	0.4%	0.4%	0.0%	1.3%	0.0%	3.0%	2.6%	0.0%	6.1%	0.0%	0.4%	2.2%	0.0%	73.9%	4.3%	3.5%	1.7%	0.0%	0.0%	0.0%
373	0.0%	0.0%	0.0%	0.0%	0.0%	22.6%	2.2%	0.0%	3.9%	0.0%	0.0%	57.4%	0.0%	0.9%	1.3%	11.7%	0.0%	0.0%	0.0%	0.0%
374	0.4%	2.6%	0.0%	0.0%	0.0%	0.0%	0.0%	2.2%	28.7%	6.1%	4.3%	0.0%	0.0%	8.7%	44.3%	0.0%	1.3%	1.3%	0.0%	0.0%
375	0.0%	0.0%	0.0%	0.0%	0.0%	0.0%	0.0%	0.0%	19.1%	0.0%	0.0%	0.0%	4.8%	0.0%	62.2%	11.3%	2.6%	0.0%	0.0%	0.0%
376	0.0%	0.0%	0.0%	0.0%	0.0%	0.0%	0.0%	11.7%	0.0%	65.2%	18.7%	0.0%	0.0%	0.4%	0.0%	0.0%	0.0%	3.9%	0.0%	0.0%
377	21.3%	0.0%	0.4%	1.3%	0.9%	2.2%	0.9%	17.0%	0.4%	10.4%	0.0%	0.0%	7.0%	0.9%	0.0%	3.9%	3.0%	30.0%	0.0%	0.4%
378	1.3%	0.0%	53.9%	41.3%	0.0%	0.0%	0.0%	0.4%	0.0%	0.0%	0.4%	0.4%	0.0%	0.9%	0.0%	0.4%	0.4%	0.4%	0.0%	0.0%
379	0.0%	0.0%	0.0%	0.0%	0.4%	0.0%	0.0%	26.5%	0.0%	3.5%	7.0%	0.0%	0.0%	0.0%	0.0%	0.0%	0.0%	62.6%	0.0%	0.0%
380	99.1%	0.4%	0.0%	0.0%	0.0%	0.0%	0.0%	0.0%	0.0%	0.0%	0.0%	0.0%	0.0%	0.0%	0.0%	0.4%	0.0%	0.0%	0.0%	0.0%
381	5.2%	0.0%	3.5%	35.2%	0.0%	0.9%	1.3%	0.0%	0.9%	0.4%	0.9%	2.6%	0.0%	20.4%	25.2%	1.3%	2.2%	0.0%	0.0%	0.0%
382	54.3%	0.0%	0.4%	1.3%	0.0%	0.4%	1.3%	0.4%	2.6%	2.2%	0.0%	5.2%	0.0%	4.3%	7.0%	15.2%	5.2%	0.0%	0.0%	0.0%
383	0.9%	0.0%	0.0%	0.0%	0.0%	0.0%	0.0%	21.8%	0.0%	21.4%	13.1%	0.0%	0.0%	0.0%	0.0%	0.0%	9.6%	33.2%	0.0%	0.0%
384	0.0%	0.0%	0.0%	0.0%	0.0%	0.0%	0.0%	14.5%	0.0%	71.1%	0.9%	0.0%	0.0%	0.0%	0.0%	0.0%	0.0%	13.6%	0.0%	0.0%

Table S3: List of Strains Used

Each of the strains used in the paper is represented in table S3. From left to right the columns represent the name of the completed strain, the base strain used to create the strain, the plasmids in the strain, and what each plasmid in the strain is composed of.

Strain	Base Strain	Plasmid used	Integrated Elements
CZ020	T7 Express	pMG1130	His10_MBP_NasR (from Koxytoca)
CZ021	T7 Express	pCZ019	His10_MBP_NasR (from Koxytoca) M376A
CZ022	T7 Express	PCZ012	His10_MBP_NasR (from Koxytoca) E358A
CZ023	T7 Express	pCZ005	His10_MBP_NasR (from Koxytoca) Q329A
CZ024	T7 Express	pCZ002	His10_MBP_NasR (from Koxytoca) D302A
CZ025	T7 Express	pCZ021	His10_MBP_NasR (from Koxytoca) K372A
CZ026	T7 Express	pCZ018	His10_MBP_NasR (from Koxytoca) M373A
CZ027	T7 Express	pCZ013	His10_MBP_NasR (from Koxytoca) W362A
CZ028	T7 Express	pCZ010	His10_MBP_NasR (from Koxytoca) K347A
CZ030	T7 Express	pCZ006	His10_MBP_NasR (from Koxytoca) E338A
CZ031	T7 Express	pCZ017	His10_MBP_NasR (from Koxytoca) D371A
CZ032	T7 Express	pCZ004	His10_MBP_NasR (from Koxytoca) E322A
CZ033	T7 Express	pCZ020	His10_MBP_NasR (from Koxytoca) E344A
CZ034	T7 Express	pCZ008	His10_MBP_NasR (from Koxytoca) K341A
CZ035	T7 Express	pCZ009	His10_MBP_NasR (from Koxytoca) K345A
CZ036	T7 Express	pCZ016	His10_MBP_NasR (from Koxytoca) M370A
CZ037	T7 Express	pCZ007	His10_MBP_NasR (from Koxytoca) R340A
CZ038	T7 Express	pCZ011	His10_MBP_NasR (from Koxytoca) T352A
CZ039	T7 Express	pCZ015	His10_MBP_NasR (from Koxytoca) R366A
CZ040	T7 Express	pCZ030	His10_MBP_NasR (from Koxytoca) K347R
CZ041	T7 Express	pCZ029	His10_MBP_NasR (from Koxytoca) K345R
CZ042	T7 Express	pCZ028	His10_MBP_NasR (from Koxytoca) R340K
CZ049	T7 Express	pCZ023	His10_MBP_NasR (from Koxytoca) E191A
CZ050	T7 Express	pCZ024	His10_MBP_NasR (from Koxytoca) R193A
CZ067	T7 Express	pCZ035	His10_MBP_NasR (from Koxytoca) Q194A
CZ068	T7 Express	pCZ036	His10_MBP_NasR (from Koxytoca) E359A
CZ069	T7 Express	pCZ037	His10_MBP_NasR (from Koxytoca) E175A
CZ070	T7 Express	pCZ038	His10_MBP_NasR (from Koxytoca) R176A
CZ072	T7 Express	pCZ040	His10_MBP_NasR (from Koxytoca) Q203A
MG5304 #1	B. subtilis 168	pMG1127	amyE:: Pconst-P1P2T (from NasF leader region)-YFP
CZ074	T7 Express	pCZ041	His10_MBP_NasR (from Koxytoca) W362F

Table S4: List of Oligonucleotides used

Each of the oligonucleotides used in the paper is represented below. From left to right the columns represent; what mutation the oligonucleotides was used for, what the oligonucleotide was used for, the name of the oligonucleotide, and the sequence of the oligonucleotide.

Mutation Name	Oligonucleotide Use	Oligo Name	Oligonucleotide sequence
W299A	Forward Primer	CZ001	TCCAGCAGACgcgCAAGAGGATAGTATTGC
W299A	Reverse Primer	CZ002	GGAAGCTGAGCCTCTGGC
D302A	Forward Primer	CZ003	CTGGCAAGAGgcgAGTATTGCACTTAG
D302A	Reverse Primer	CZ004	TCTGCTGGAGGAAGCTGA
L315A	Forward Primer	CZ005	GCTCCTTCCAgcgGTTCGCCAGC
L315A	Reverse Primer	CZ006	TGTTTATCGAGTCTAAGTG
E322A	Forward Primer	CZ007	GCAGGCGCATgcgTTACAACAGCTGTC
E322A	Reverse Primer	CZ008	TGGCGAACGAGTGGAAGG
Q329A	Forward Primer	CZ009	GCTGTCAGGCgcgCTGGCTAGTTTG
Q329A	Reverse Primer	CZ010	TGTTGTAATTCATGCGCC
E338A	Forward Primer	CZ011	GGATGCTCTGgcgGAACGTAAATTG
E338A	Reverse Primer	CZ012	TTCAAACTAGCCAGTTGG
R340A	Forward Primer	CZ013	TCTGGAAGAAgcgAAATTGATCGAGAAAGCC
R340A	Reverse Primer	CZ014	GCATCCTTCAAACTAGCC
K341A	Forward Primer	CZ015	GGAAGAACGTgcgTTGATCGAGAAAGC
K341A	Reverse Primer	CZ016	AGAGCATCCTTCAAACTAG
K345A	Forward Primer	CZ017	ATTGATCGAGgcgGCCAAATCCGTC
K345A	Reverse Primer	CZ018	TTACGTTCTTCCAGAGCATC
K347A	Forward Primer	CZ019	CGAGAAAGCCgcgTCCGTCCTTATG
K347A	Reverse Primer	CZ020	ATCAATTTACGTTCTTCCAG
T352A	Forward Primer	CZ021	CGTCCTTATGgcgTACCAGGGGA
T352A	Forward Primer	CZ022	GATTTGGCTTTCTCGATCAATTTACGTTC
E358A	Reverse Primer	CZ023	GGGGATGCAGgcgGAACAAGCGT
E358A	Forward Primer	CZ024	TGGTACGTCATAAGGACGGATTTG
W362A	Reverse Primer	CZ025	GGAACAAGCGgcgCAGGCGCTTC
W362A	Forward Primer	CZ026	TCCTGCATCCCCTGGTAC
Q363A	Reverse Primer	CZ027	ACAAGCGTGGgcgGCGCTTCGGAAAATG
Q363A	Forward Primer	CZ028	TCCTCCTGCATCCCCTGG
R366A	Reverse Primer	CZ029	GCAGGCGCTTgcgAAAATGGCAATG
R366A	Forward Primer	CZ030	CACGCTTGTTCCTCCTGC
M370A	Reverse Primer	CZ031	GAAAATGGCAgcgGATAAAAATCAAAGAATGG TCGAGATTGCACGG
M370A	Forward Primer	CZ032	CGAAGCGCCTGCCACGCT
D371A	Reverse Primer	CZ033	AATGGCAATGgcgAAAAATCAAAGAATGGTCG AGATTGC
D371A	Forward Primer	CZ034	TTCCGAAGCGCCTGCCAC

N373A	Reverse Primer	CZ035	AATGGATAAAgcgCAAAGAATGGTCGAGATTG CACG
N373A	Forward Primer	CZ036	GCCATTTTCCGAAGCGCC
M376A	Reverse Primer	CZ037	AAATCAAAGAgcgGTCGAGATTGCAC
M376A	Forward Primer	CZ038	TTATCCATTGCCATTTTCC
E344A	Reverse Primer	CZ039	TAAATTGATCgcgAAAGCCAAATCC
E344A	Forward Primer	CZ040	CGTTCTTCCAGAGCATCC
K372A	Reverse Primer	CZ041	GGCAATGGATgcgAATCAAAGAATGGTCGAG
K372A	Forward Primer	CZ042	ATTTTCCGAAGCGCCTGC
R340K	Forward Primer	CZ043	TCTGGAAGAAaaaAAATTGATCGAGAAAGC
R340K	Reverse Primer	CZ044	GCATCCTTCAAACTAGCC
K345R	Forward Primer	CZ045	ATTGATCGAGcgtGCCAAATCCGTC
K345R	Reverse Primer	CZ046	TTACGTTCTTCCAGAGCATC
K347R	Forward Primer	CZ047	CGAGAAAGCCcgtTCCGTCCTTATG
K347R	Reverse Primer	CZ048	ATCAATTTACGTTCTTCCAG
E191A	Forward Primer	CZ065	ATTCTCCGACgcaCTTAGACAGCAGCTGGTAGA TAGAATTGATG
E191A	Reverse Primer	CZ066	TGCCCGCGCGCAAAGCCC
R193A	Forward Primer	CZ067	CGACGAACTTgcaCAGCAGCTGGTAGATAG
R193A	Reverse Primer	CZ068	GAGAATTGCCCGCGCGCA
E191A R193A	Forward Primer	CZ073	tgcaCAGCAGCTGGTAGATAGAATTGATG
E191A R193A	Reverse Primer	CZ074	agtgcGTCGGAGAATTGCCCGCG
	Used to make the NasF leader region for Transcription Antitermination	JRG105	AAAGATATCCTTACAGCACAAGAGC
Q194A	Forward Primer	JRG664	CGAACTTAGAgcaCAGCTGGTAGATAGAATTG ATGGACAACAGCC
Q194A	Reverse Primer	JRG665	TCGGAGAATTGCCCGCGC
E359A	Forward Primer	JRG666	GATGCAGGAGgcaCAAGCGTGGC
E359A	Reverse Primer	JRG667	CCCTGGTACGTCATAAGGACG
E175A	Forward Primer	JRG668	GGCTGGGCAAgcaCGTGCTTTGG
E175A	Reverse Primer	JRG669	AGCTCTTTTCCCTGCATGAAACTG
R176A	Forward Primer	JRG670	TGGGCAAGAAgcaGCTTTGGGAGCTTTGG
R176A	Reverse Primer	JRG671	GCCAGCTCTTTTCCCTGC
R50A	Forward Primer	JRG672	GCAATGCGAAgcaGGGGCTTCTAACATTTGG
R50A	Reverse Primer	JRG673	AGCATGTGCACGAGAGCA
Q203A	Forward Primer	JRG674	AATTGATGGAgcaCAGCCTTGTTTTGAC
Q203A	Reverse Primer	JRG675	CTATCTACCAGCTGCTGTC
	Used to make the NasF leader region for Transcription Antitermination	MG327	TTCGTTTCATCGCTACCTCCAT
W362F	Forward Primer	JRG676	GGAACAAGCGttcCAGGCGCTTC
